# Solvent Dependence of the Monomer–Dimer Equilibrium of Ketone‐Substituted Triscatecholate Titanium(IV) Complexes

**DOI:** 10.1002/chem.202001053

**Published:** 2020-07-20

**Authors:** A. Carel N. Kwamen, Judith Jenniches, Iris M. Oppel, Markus Albrecht

**Affiliations:** ^1^ Institut für Organische Chemie RWTH Aachen University Landoltweg 1 Aachen 52074 Germany; ^2^ Institut für Anorganische Chemie RWTH Aachen University Landoltweg 1 Aachen 52074 Germany

**Keywords:** helicates, intermolecular interactions, self assembly, solvent effect, thermodynamics

## Abstract

Hierarchical helicates based on ketone‐substituted titanium(IV)triscatecholates show different monomer‐dimer behavior depending on different solvents. The dimerization constants of a whole series of differently alkyl‐substituted complexes is analyzed to show that the solvent has a very strong influence on the dimerization. Hereby, effects like solvophobicity/philicity, sterics, electronics of the substituents and weak side‐chain—side‐chain interactions seem to act in concert.

## Graphical abstract

**The behavior of the monomer–dimer equilibrium** of hierarchical helicates formed from keto‐catechol ligands strongly depends on the solvent that is used. Based on the respective solvents, different side‐chain—side‐chain interaction modes such as sterics, solvophobicity/philicity and probably even London dispersion become highly influential for the stabilization or destabilization of the dimers.

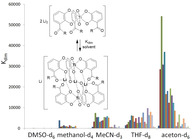

## Introduction

Over the last 50 years supramolecular chemistry has evolved to an important independent branch of chemistry combining principles of the traditional disciplines (inorganic, organic, physical chemistry) and connecting those to biochemistry, material science or nanotechnology.^1^

**Scheme 1:**
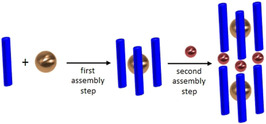
Hierarchical formation of helicate type complexes by incorporation of a metal ion into the spacer of the helicating ligand.

More than 30 years ago Lehn introduced the helicates as coordination compounds in which two or more linear ligand strands wrap around two or more metal ions.^2^ If the helicating ligands are not covalently linked but contain a non‐covalent connecting point (e.g. a metal ion or a hydrogen bond), helicate type coordination compounds may be formed in a hierarchical process (Scheme 1).^3^ Several “hierarchical” helicates as well as closely related cluster helicates have been described in the literature.^4^

**Scheme 2:**
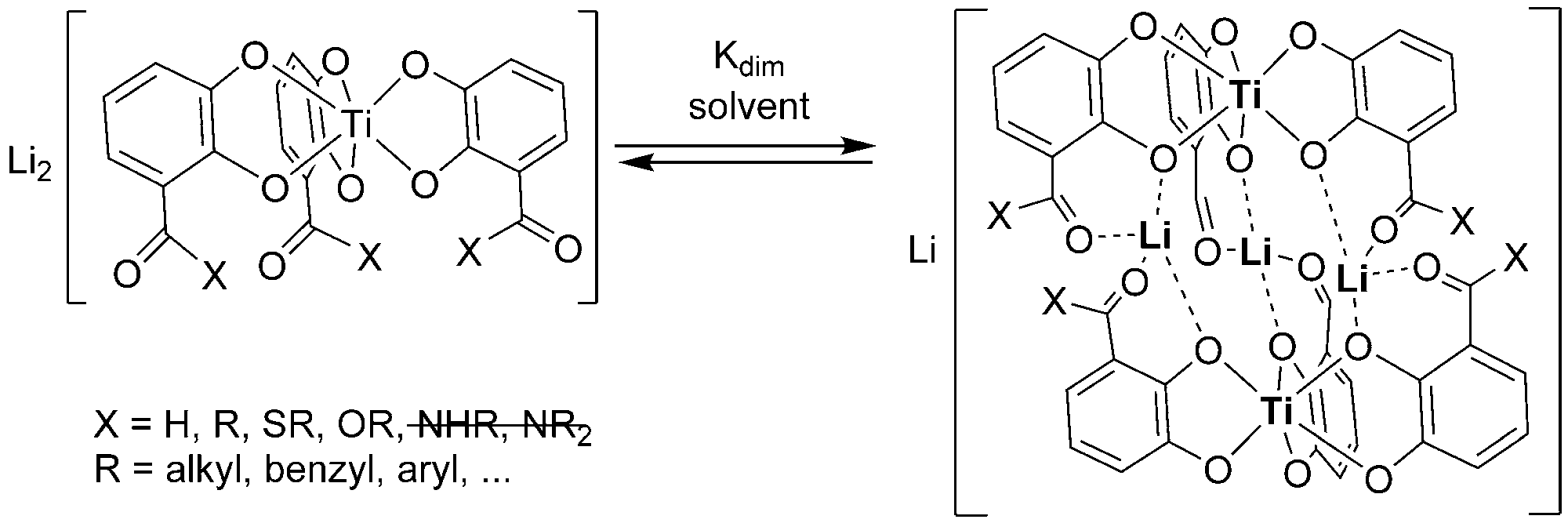
The lithium dependent monomer‐dimer equilibrium based on carbonyl‐substituted titanium(IV) triscatecholates.

In 2005 we described a hierarchical helicate based on 3‐carbonyl‐substituted catecholate ligands forming initially a mononuclear triscatecholate titanium(IV) complex which in the presence of lithium counter cations dimerizes to a triple‐lithium bridged coordination compound. The carbonyl may be an aldehyde, ketone, thioester or ester.^5^ For several reasons no dimer formation has been observed for amide derivatives yet: with secondary amides an NH⋅⋅⋅O_catecholate_ hydrogen bond is blocking the lithium binding site, while tertiary amides are sterically too demanding for dimer formation (Scheme 2).

The hierarchically formed triscatecholate titanium(IV) helicates are exceptional in comparison to other hierarchical helicates. In the solid, the dimeric helicates are present while in solution the lithium bridged systems slowly reach the thermodynamic equilibrium between monomer and dimer.^6^

The equilibrium ratio between monomer and dimer depends on the strength of lithium binding in the dimer or the ease of lithium removal, respectively. Thus, the kinds of carbonyl donors as well as of the solvents are highly influential on the equilibrium state. In addition, weak side‐chain interactions significantly can contribute to dimer stabilization or destabilization.^6^

**Figure 1:**
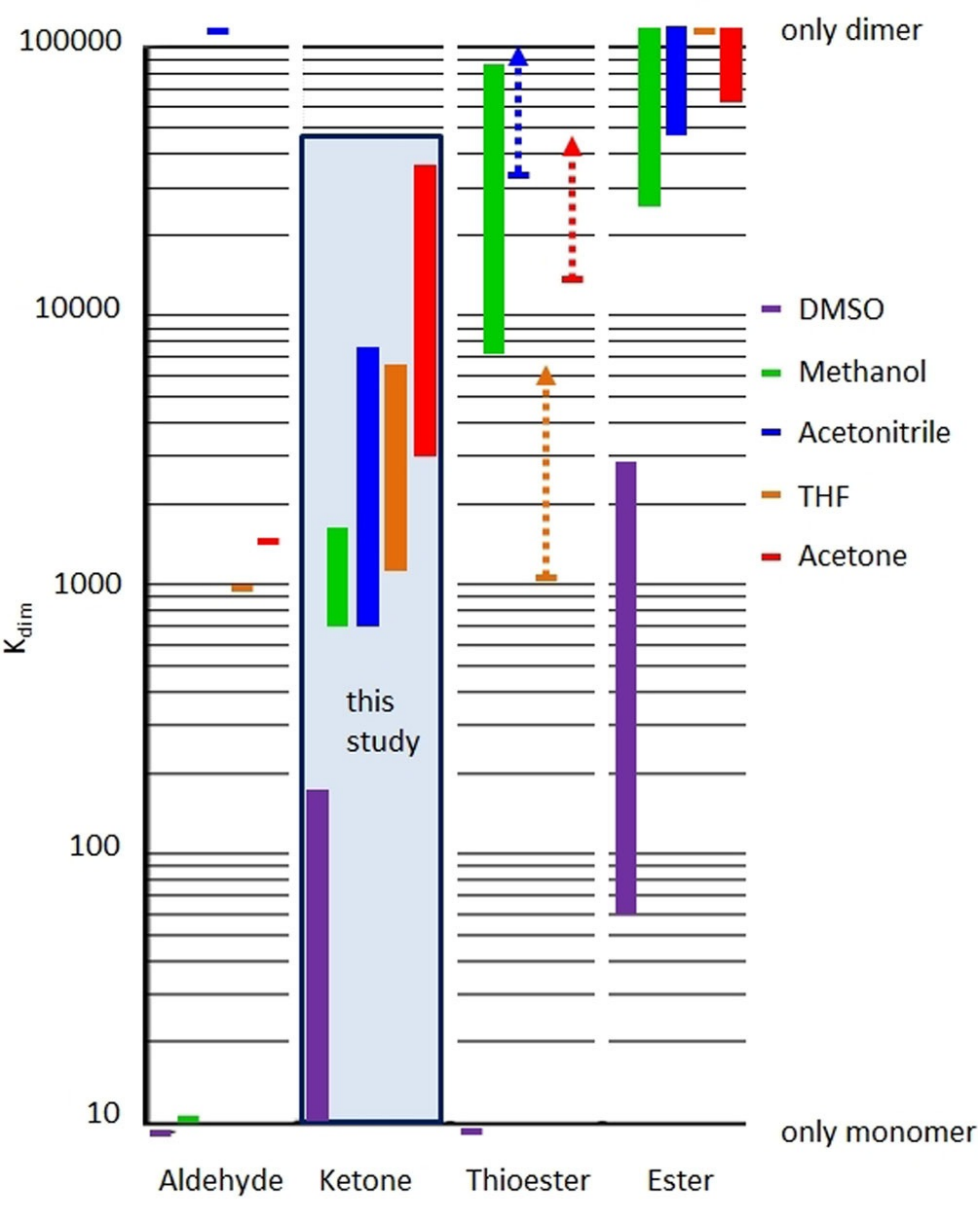
Stability domains in which the lithium dependent monomer dimer equilibrium of carbonyl substituted triscatecholate titanium(IV) complexes can be observed depending on the carbonyl moiety as well as on the solvent. The results of the present study on ketone derivatives are highlighted in the box.

To illustrate the solvent dependence: the complexes of 2,3‐dihydroxybenzaldehyde as ligand at room temperature show dimerization constants of *K*_dim_=10 ([D_4_]MeOH), 950 ([D_8_]THF) or 1330 ([D_6_]acetone). In [D_6_]DMSO or D_2_O only monomer and in [D_3_]acetonitrile only dimer is observed.^5^ Thus depending on the solvent, the whole spectrum from monomer to dimer can be detected by NMR spectroscopy at ambient conditions (Figure 1).

Intense recent studies were focusing on the ester or thioester derivatives in [D_6_]DMSO or [D_4_]MeOH solution, respectively. Those solvents provide dimer stability windows which make a comparative investigation within the oxo‐6 or thioester^5^ series depending on different side‐chains possible. However, it would be of major interest to perform related studies which simultaneously allow the evaluation of the influence of the side chain as well as of different solvents.

Therefore, our focus now was shifted back to the ketone based catecholate ligands **1**‐H_2_ and found out that they are ideal candidates for the systematic investigation of the dimer stability with variation of the substituents as well as of the solvents. Some of the ketone derivatives were already studied in [D_4_]MeOH.5a Some new complexes are added in here and significantly different dimerization behavior is observed depending on the solvents [D_6_]DMSO, [D_4_]MeOH, [D_3_]acetonitrile, [D_8_]THF and [D_6_]acetone.

## Results and Discussion

**Scheme 3:**
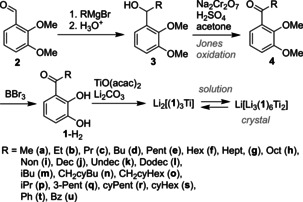
Preparation of the ligands discussed in this study.

The required ligands **1**‐H_2_ were prepared by Grignard addition of alkyl Grignard reagents to dimethoxybenzaldehyde **2** followed by Jones oxidation of the alcohols **3** and final ether cleavage of the protecting groups at **4**. The obtained ligands **1**‐H_2_ (3 equiv) were coordinated to titanoylbis(acetylacetonate) in the presence of lithium carbonate to obtain the hierarchical helicates Li[Li_3_(**1**)_6_Ti_2_] which in solution are in equilibrium with the monomeric species Li_2_[(**1**)_3_Ti] (Scheme 3).^5^

The catechol ligands bear ketone substituents of the n‐alkane series from methyl to dodecyl (**1 a**–**l**‐H_2_), β‐branched substituents (**1 m–o‐**H_2_), secondary substituents (**1 p–s**‐H_2_), and substituents with phenyl groups (**1 t,u**‐H_2_).^7^

**Figure 2:**
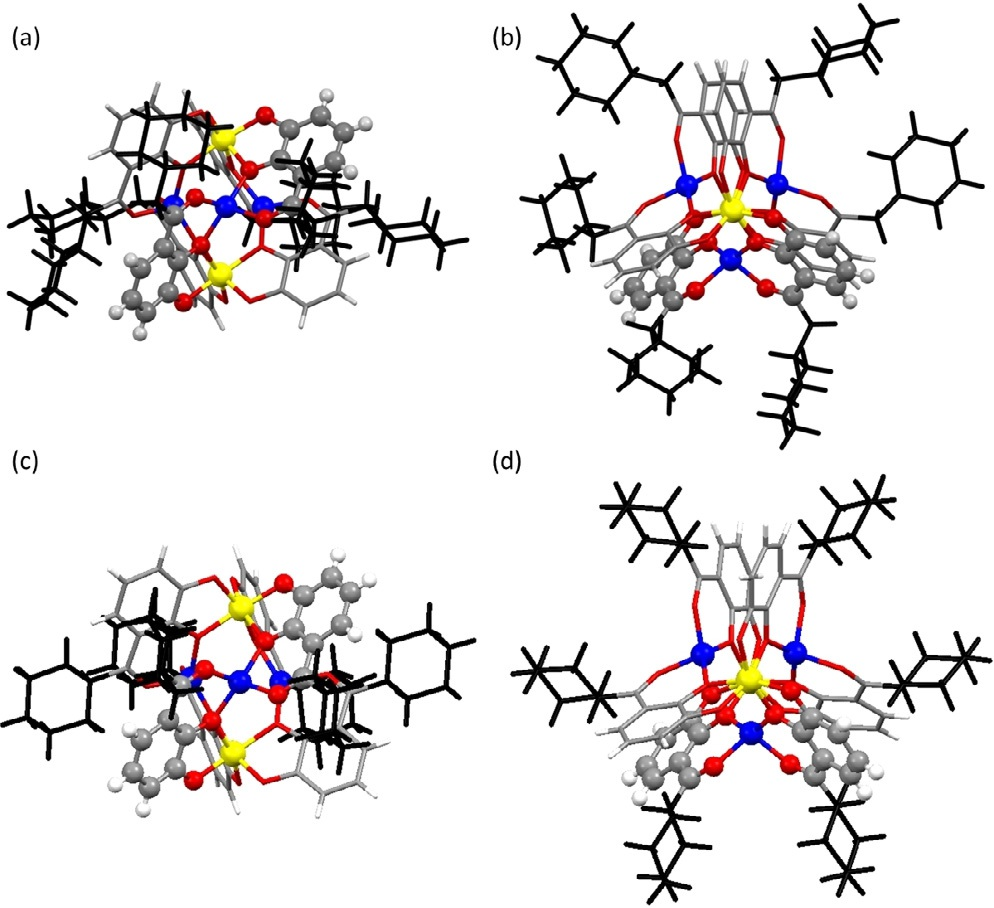
Structure of the anion [Li_3_(**1 o**)_6_Ti_2_]^−^ as observed in the crystal of K[Li_3_(**1 o**)_6_Ti_2_] (side (a) and top view (b)) and of [Li_3_(**1 s**)_6_Ti_2_]^−^ (side (c) and top view (d)). Grey: C, white: H, red: O, blue: Li, yellow: Ti, the cyclohexylmethyl substituents are shown in black.

The crystal structure of the ethyl ketone Li[Li_3_(**1 b**)_6_Ti_2_] has been described earlier.5a In addition, the structure of the more sterically demanding cyclohexyl methyl K[Li_3_(**1 o**)_6_Ti_2_] and cyclohexyl‐substituted complex Li[Li_3_(**1 s**)_6_Ti_2_] have been obtained now. Figure 2 a shows the side view of the anion [Li_3_(**1 o**)_6_Ti_2_]^−^ revealing the connecting bis‐titanium tris‐lithium center while the top view (Figure 2 b) shows the relative orientation of the cyclohexylmethyl substituents. Hereby the cyclohexyl rings adopt roughly an alternating position with the “plane” of the six‐membered ring orientated parallel or orthogonal to the Ti⋅⋅⋅Ti axis. This allows a close packing with H⋅⋅⋅H distances of 2.29–2.98 Å between neighboring T‐shaped cyclohexyl rings. All cyclohexyl moieties adopt the chair conformation with an equatorial position of the methylene unit.^8^

Li[Li_3_(**1 s**)_6_Ti_2_] is the sterically most crowded hierarchically formed dimer of this kind which has been structurally characterized so far. Due to the limited space around the central core the cyclohexyl planes have to orientate parallel to the Ti⋅⋅⋅Ti axis. In addition, the dimer has to “stretch” resulting in a somewhat longer Ti⋅⋅⋅Ti distance of 5.562(1) Å in [Li_3_(**1 s**)_6_Ti_2_]^−^ compared to 5.444(1) Å in [Li_3_(**1 o**)_6_Ti_2_]^−^.

Dimerization constants of the complexes Li[Li_3_(**1 a**–**u**)_6_Ti_2_] were determined by proton NMR at 295 K in [D_6_]DMSO, [D_4_]MeOH, [D_3_]acetonitrile, [D_8_]THF and [D_6_]acetone (Table 1).

**Table 1:** Solvent dependent dimerization constants *K*_dim_ for the equilibrium between two monomers Li_2_[(**1**)_3_Ti] and one dimer Li[Li_3_(**1**)_6_Ti_2_] as obtained at 295 K by proton NMR integration at a concentration of 2×10^−3^ mol L^−1^.

Ligand (R)	[D_6_]DMSO	CD_3_OD	[D_3_]MeCN	[D_8_]THF	(D_3_C)_2_C=O
**1 a** (Me)	monomer	3890±505	715±84	1430±177	1240±152
**1 b** (Et)	monomer	785^[a]^	3260±420	1960±247	28 590±3913
**1 c** (Pr)	35±3	1110^[a]^	7460±990	3170±408	54 150±7979
**1 d** (Bu)	55±5	1500^[a]^	5400±710	4000±521	30 780±4219
**1 e** (Pent)	25±2	1015^[a]^	5520±727	6215±822	36 980±5082
**1 f** (Hex)	180±19	965^[a]^	3120±402	6075±802	16 560±2245
**1 g** (Hept)	90±9	725^[a]^	3640±472	3260±420	18 030±2448
**1 h** (Oct)	115±12	1425^[a]^	3890±505	4960±651	12 320±1659
**1 i** (Non)	110±11	1125±137	5515±677	2570±328	19 250±2618
**1 j** (Dec)	90±9	665^[a]^	5250±70	5480±721	15 900±2884
**1 k** (Undec)	85±8	740±88	5825±83	2780±356	12 350±1665
**1 l** (Dodec)	80±8	1200^[a]^	5155±677	5270±692	8560±1142
**1 m** (iBu)	175±18	175±18	600±70	7570±1007	1090±132
**1 n** (CH_2_cyBu)	135±14	100±10	700±83	3150±406	2950±378
**1 o** (CH_2_cyHex)	95±9	55±5	540±63	2180±276	6690±887
**1 p** (iPr)	25±2	7±1	1730±216	9720±1301	1040±126
**1 q** (3‐Pent)	monomer	4±1	505±58	6390±845	2000±252
**1 r** (cyPent)	160±17	40±4	375±42	7970±1061	4700±615
**1 s** (cyHex)	monomer	30±3	965±116	6390±845	2560±326
**1 t** (Ph)	160±17	70±7	130±13	950±114	455±52
**1 u** (Bz)	50±5	146±15	290±32	675±79	985±119

[a] From reference [5a].

**Figure 3:**
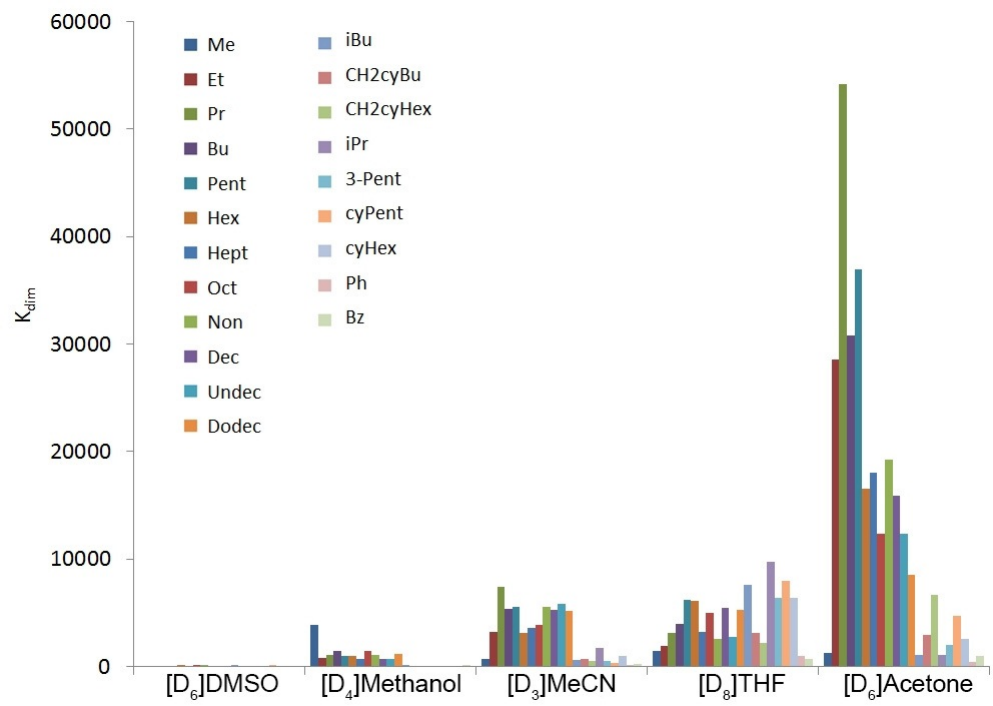
Dimerization constants *K*_dim_ (at 295 K) of the complexes Li[Li_3_(**1 a**–**u**)_6_Ti_2_ in [D_6_]DMSO, [D_4_]MeOH, [D_3_]MeCN, [D_8_]THF and [D_6_]acetone.

**Figure 4:**
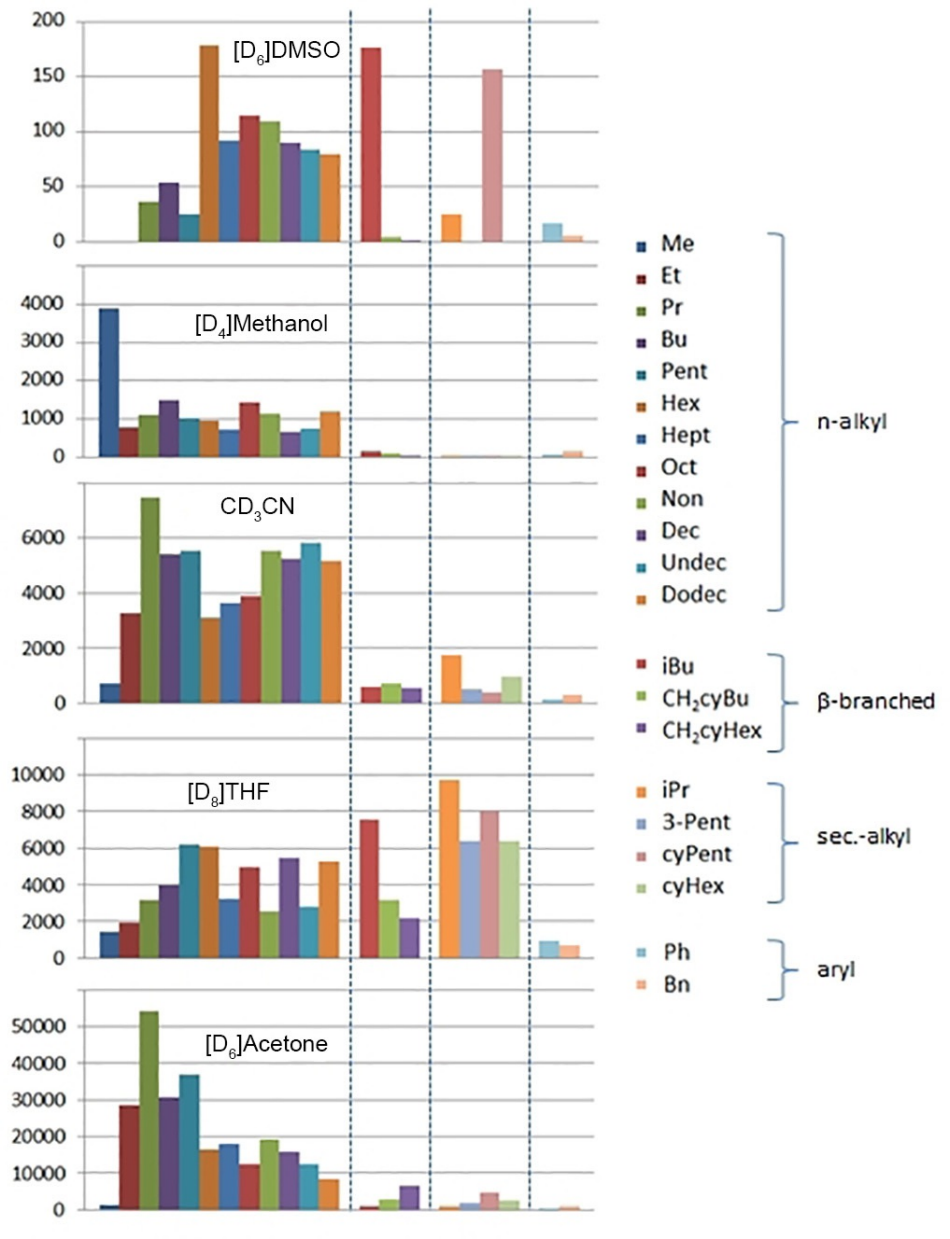
*K*_dim_ (at 295 K) of the complexes Li[Li_3_(**1 a**–**u**)_6_Ti_2_ as observed in different solvents.

Table 1 and Figure 3, and Figure 4 summarize the obtained dimer stabilities. It is obvious that the dimerization constants rise in the order [D_6_]DMSO<[D_4_]MeOH<[D_3_]acetonitrile≤[D_8_]THF<[D_6_]acetone as it is roughly summarized in the highlighted box in Figure 1. The observed trend in dimerization constants can neither be correlated with the polarity of the solvent (Reichardt polarity parameters: ET=45.1 (DMSO), 55.4 (MeOH), 45.6 (MeCN), 37.4 (THF) and 42.2 (acetone))^9^ nor with the ability to dissolve lithium cations.^10^ However, the dimerization tendency roughly follows the lipophobicity/philicity of the respective solvent. Alkanes are not soluble in DMSO, they show low solubility in methanol and acetonitrile and they are more or less miscible with THF and acetone. Thus, more hydrophilic solvents stabilize the highly charged monomer with two “free” lithium counter cations in which oxygen atoms are exposed to the surface of the complex while lipophilic solvents prefer the less charged dimer with the oxygen atoms buried within the complex.

**Figure 5:**
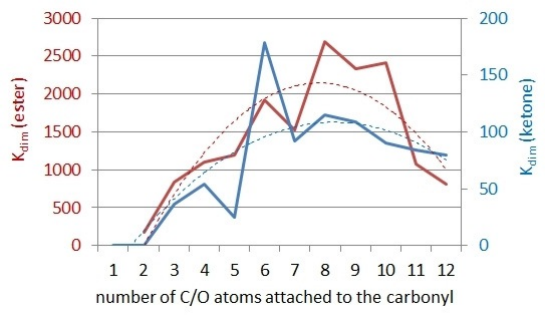
*K*_dim_ of the n‐alkyl (blue) and n‐alkoxy (red) substituted complexes plotted against the chain length (number of C or C+O atoms) of the respective side chain. The dotted lines represent the respective trend lines.

Different trends can be observed for the dimerization constants based on the different solvents (Figure 4): **[D_6_]DMSO**: The *K*_dim_ values in [D_6_]DMSO follow trends as observed earlier for the corresponding esters.^6^ For the methyl‐ as well as ethyl‐ketones only monomers are observed while with gradually increasing numbers of carbon atoms of the n‐alkyl substituent *K*_dim_ increases reaching a maximum for the hexyl compound. With longer n‐alkyls *K*_dim_ stepwise decreases again. Figure 5 shows a comparison of the dimerization constants of the n‐alkyl‐substituted ketone (blue) and ester derivatives (red) revealing similar shapes of the trend lines (dotted lines).

The branched compounds show in most cases much lower dimerization tendencies than the linear ones with the exception of the isobutyl and cyclopentyl substituted complexes. The complexes with aromatic side chains prefer the formation of the monomer due to the high solvophilicity of aromatics in DMSO. The exceptionally high *K*_dims_ of the isobutyl and cyclopentyl ketones may be due to some interactions between the side chains in the dimeric helicates (e.g. dispersion^11^) in addition to solvophobic effects. The isobutyl complex hereby may be compared to the corresponding isopropyl ester in which stabilizing dispersive interactions have been verified.^6^

**[D_4_]Methanol**:5a In deuterated methanol there seems to be virtually no difference in the electronic influence of different substituents. The observed dimerization constants lead to the impression that here only sterics are controlling the dimer stability. The methyl ketone as the sterically least demanding group results in the highest *K*_dim_. The longer n‐alkyl derivatives show very similar dimer stabilities. However, it is reduced in the complexes with sterically more demanding β‐ and even more with α‐branched side chains.

**[D_3_]MeCN**: In deuterated acetonitrile the dimerization constant gradually increases from the methyl to the n‐propyl ketone and after this reaches a plateau starting with butyl. A drop in *K*_dim_ is found for the hexyl to octyl substituted derivatives. The initial increase of *K*_dim_ can be attributed to the increasing donor ability of the substituents while later on mainly steric effects are important. This results in low dimerization constants of the complexes with branched side chains.

**[D_8_]THF**: Starting with the methyl ketone the dimerization constant gradually increases until it reaches the pentyl derivative. The hexyl and higher n‐alkyl substituted complexes show a strong even–odd behavior with the even alkyl groups resulting in higher and the odd in lower dimerization constants. This even–odd behavior is an indication for a direct interaction between the alkyl chains.^12^ The *K*_dim_ values of the β‐branched derivatives are related to the dimerization constants of the n‐alkyls while the bulky secondary ketones result in unusually high ones (even higher than the n‐alkyls).

**[D_6_]Acetone**: The dimerization behavior of the n‐alkanes in [D_6_]acetone indicates a strong influence of the electron donating alkyl groups from methyl to ethyl to n‐propyl leading to increasing *K*_dim_ values. With longer alkyl chains the dimerization constants gradually decrease showing some even/odd alternating behavior. Due to higher steric demands the β‐branched systems possess somewhat lower and the α‐branched very low dimerization constants.

Our investigations show that there is a strong solvent dependence of the monomer dimer equilibrium of Li[Li_3_(**1 a**–**u**)_6_Ti_2_] based on different effects in different solvents resulting in very different stability patterns of the set of compounds in the investigated solvents.^13^ [D_6_]DMSO, [D_3_]MeCN and [D_6_]acetone show more or less easy to explain patterns of *K*_dim_: initially *K*_dim_ increases with growing chain length while it decreases with longer chains. This may be due to an entropy effect as observed for the corresponding n‐alkyl esters.

The solvents [D_4_]MeOH and [D_8_]THF behave in an unexpected way: In case of [D_4_]MeOH only steric effects seem to be influential, leading to lower *K*_dim_ values with bulkier side chains. In [D_8_]THF higher dimer stability is observed with more bulky groups. This observation may be due to a direct attractive interaction between the side chains in this solvent. Bulkier groups are able to have direct contact to each other while less bulky groups have to adopt their conformation appropriately.^14^ This interpretation is supported by the observation of an even odd behavior of the dimerization constants in case of the long n‐alkyl chains.

## Conclusions

The monomer dimer equilibrium of ketocatechol based hierarchical helicates Li_2_[(**1**)_3_Ti]/Li[Li_3_(**1**)_6_Ti_2_] is an ideal tool to investigate weak interactions of different side chains in different solvents.^15^ Thus it represents an interesting alternative to Wilcox molecular balance.^16^ Variation of the solvent leads to dramatically different patterns of the stability constants revealing the influence of effects such as sterics, electronics and side‐chain—side‐chain interactions. Often the concerted influence of all the effects is clear. However, in [D_4_]MeOH only sterics seem to be responsible for the dimer stability. Some observed dimerization constants are exceptionally high (Li[Li_3_(**1 m,r**)_6_Ti_2_] in [D_6_]DMSO, Li[Li_3_(**1 p**)_6_Ti_2_] in [D_3_]acetonitrile and Li[Li_3_(**1 m,** ***p*** **‐s**)_6_Ti_2_] in [D_8_]THF). In those cases, some attractive side chain‐side chain interactions between the bulky groups seem to become important, which may be based on London dispersions.^17^

## Conflict of interest

The authors declare no conflict of interest.

## Supporting information

As a service to our authors and readers, this journal provides supporting information supplied by the authors. Such materials are peer reviewed and may be re‐organized for online delivery, but are not copy‐edited or typeset. Technical support issues arising from supporting information (other than missing files) should be addressed to the authors.

Supplementary
