# Supplementary material for: Solvent Dependence of the Monomer–Dimer Equilibrium of Ketone‐Substituted Triscatecholate Titanium(IV) Complexes
Source: Chemistry. 2020 Jul 20;26(46):10550–4. doi: 10.1002/chem.202001053 (PMC7496165; doi:10.1002/chem.202001053)
Supplement: Supplementary file 1 — Supplementary [file CHEM-26-10550-s001.pdf]

Chemistry–A European Journal

Supporting Information

Solvent Dependence of the Monomer–Dimer Equilibrium of Ketone-Substituted Triscatecholate Titanium(IV) Complexes

A. Carel N. Kwamen,[a] Judith Jenniches,[b] Iris M. Oppel,[b] and Markus Albrecht*[a]

Experimental Section

General Remarks: NMR spectra were obtained with Mercury 300, Varian VNMRS 400 and 600 NMR spectrometers. The compounds were analytically characterized on a LTQ Orbitrap XL for ESI-MS. IR measurements were performed using a Perkin-Elmer 100 spectrometer and Elemental analysis measured with a Heraeus CHN-O-Rapid. Melting points were determined with a BÜCHI B-540 melting point instrument and are reported uncorrected. 2,3-dimethoxybenzaldehyde and boron tribromide solution were purchased from Sigma Aldrich, while the bromoalkane and bromocycloalkane were obtained from Alfa Aesar, Sigma Aldrich and Acros Organics. All deuterated solvents were purchased from Cambridge Isotope Laboratories or euriso-top. All reactions with dry solvents were carried out under a nitrogen atmosphere in oven-dried glassware.

Dimerization constants: Dimerization constants have been obtained as described recently.[1]

General procedure for the preparation of the ligands: The corresponding Bromoalkane or Bromocycloalkane (1 eq.) was added dropwise to a solution of grounded magnesium powder (1.2 eq.) in dry diethyl ether. After 2 hrs stirring of the resulting reaction mixture at RT, 2,3-Dimethoxybenzaldehyde (2 eq.) was added dropwise and the resulting mixture stirred for 2 hrs before being quenched with aqueous HCl. The organic phase was separated, and the aqueous phase extracted twice with diethyl ether. The combined organic phase was reduced and purified by column chromatography to afford an alcohol which was oxidized by an aqueous mixture of sodium dichromate and H_2SO_4 in acetone. After 1 hr reaction, the organic phase was separated, the aqueous one extracted and the combined organic phase reduced and purified by column chromatography. The obtained ketone intermediate was then demethylated by reaction with BBr_3 in DCM at 0°C for 1 hr. The final product was obtained after quenching the reaction with MeOH, evaporating the solvent, washing with water in DCM, drying over $MgSO_4$, removing the solvent and purifying via column chromatography.

Ligands 1(a-h)-H_2 and 1j-H_2, 1l-H_2, 1p-H_2, 1u-H_2 have already been described.[2]

(2,3-dihydroxyphenyl) isobutyl ketone (1m-H_2): The ligand is prepared from 1-Bromo-2-methylpropane (165 mg, 1.21 mmol) according to the general procedure. Column chromatography (DCM, R_f = 0.15) results in the product as a brownish oil (32 %, 75 mg, 0.33 mmol). **^{1}H-NMR** (600 MHz, $CDCl_3$): δ = 12.70 (s, 1H, OH), 7.32 (dd, J = 8.1, 1.5 Hz, 1H, $H_{arom.}$), 7.12 (dd, J = 8.1, 1.5 Hz, 1H, $H_{arom.}$), 6.83 (t, J = 8.1 Hz, 1H, $H_{arom.}$), 5.76 (s, 1H, OH), 2.84 (d, J = 6.9 Hz, 2H, CH_2), 2.35-2.31 (m, 1H, CH), 0.98 (d, J = 7.0 Hz, 6H, 2×CH_3) ppm. **^{13}C-NMR** (151 MHz, $CDCl_3$): δ = 207.00 (COCH), 145.51 (C_{arom}), 120.88 (C_{arom}), 120.03 (C_{arom}), 119.44 (C_{arom}), 118.79 (C_{arom}), 47.22 (CH_2), 25.58 (CH), 22.72 (2×CH_3) ppm. **MS** (negative and positive ESI-MS, MeOH, acidified): m/z (%) = 193.0862 (100, [M-H^+], $C_{11}H_{13}O_3^-$, calcd. 193.0865); 217.0844 (10, [M+Na^+], $C_{11}H_{14}O_3Na^+$, calcd. 217.0841). **IR** (KBr): ũ (cm^{-1}) = 3457, 2917, 2849, 2644, 2530, 2324, 2169, 2085, 1990, 1935, 1736, 1636, 1452, 1369, 1315, 1264, 1168, 1132, 1093, 1051, 1022, 958, 898, 834, 773, 731, 665. **Elemental Analysis**: $C_{11}H_{14}O_3$ · 5 DCM: calcd. C= 31.05 %, H= 3.91 %; found C= 31.49 %, H= 3.87 %.

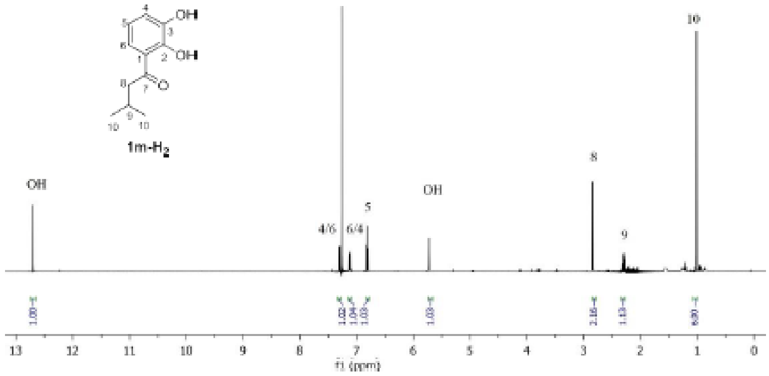

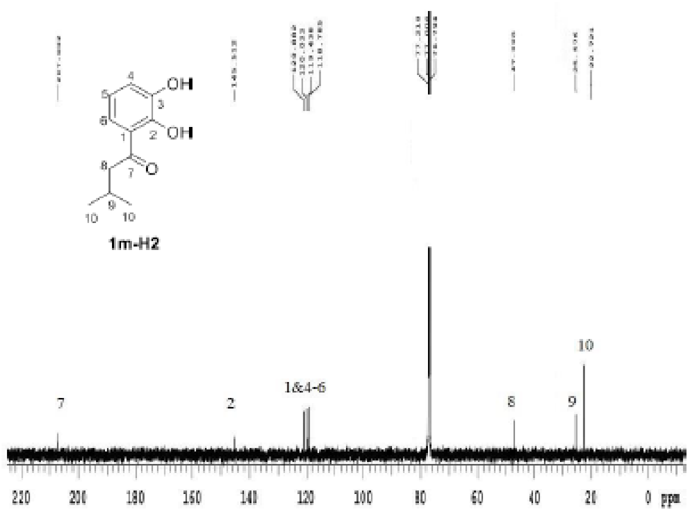

(2,3-dihydroxyphenyl) methyl cyclobutyl ketone (1n-H_2): The ligand is prepared from (Bromomethyl)cyclobutane (101 mg, 1.21 mmol) according to the general procedure. Column chromatography (DCM, R_f = 0.21) results in the product as abrown solid (36 %, 89 mg, 0.43 mmol). **M.p.:** 74ºC -75.5ºC (last solvent: DCM). **^{1}H-NMR** (600 MHz, $CDCl_3$): δ = 12.59 (s, 1H, OH), 7.30 (dd, J = 8.1, 1.4 Hz, 1H, $H_{arom.}$), 7.13 (dd, J = 8.1, 1.4 Hz, 1H, $H_{arom.}$), 6.81 (t, J = 8.0 Hz, 1H, $H_{arom.}$), 5.73 (s, 1H, OH), 3.10 (d, J = 7.3 Hz, 2H, CH_2), 3.09-2.97 (m, 1H, CH), 2.02-1.85 (m, 6H, 3×CH_2) ppm. **^{13}C-NMR** (151 MHz, $CDCl_3$): δ = 206.43 (COCH), 149.67 (C_{arom}), 145.56 (C_{arom}), 120.84 (C_{arom}), 120.21 (C_{arom}), 119.28 (C_{arom}), 118.99 (C_{arom}), 52.42 (C-8), 45.33 (CH), 28.55 (2×CH_2), 22.44 (CH_2) ppm. **MS** (negative and positive ESI-MS, MeOH, acidified): *m/z* (%) = 205.0857 (50, [M-H^+], $C_{12}H_{13}O_3^-$, calcd. 205.0865); 207.1030 (20, [M+H^+], $C_{12}H_{15}O_3^+$, calcd. 207.1021), 229.0852 (40, [M+Na^+], $C_{12}H_{14}O_3Na^+$, calcd. 229.0841). **IR** (KBr): $\tilde{\upsilon}$ (cm^{-1}) = 3874, 3455, 3053, 2955, 2868, 2709, 2507, 2324, 2096, 1999, 1907, 1633, 1448, 1373, 1323, 1264, 1109, 1052, 957, 897, 830, 736, 670. **Elemental Analysis**: $C_{12}H_{14}O_3$ · 2/3 DCM: calcd. C = 57.97 %, H = 5.89 %; found C = 57.73, H = 5.93 %.

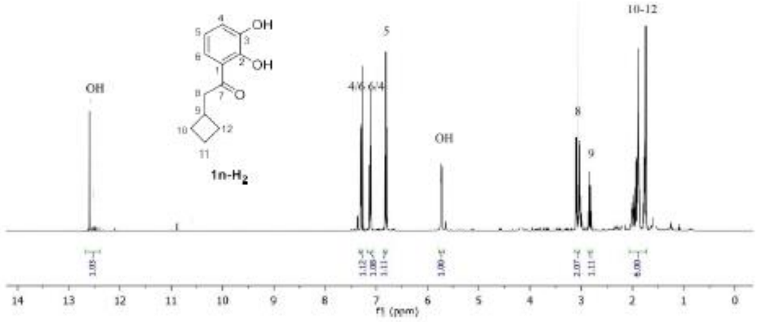

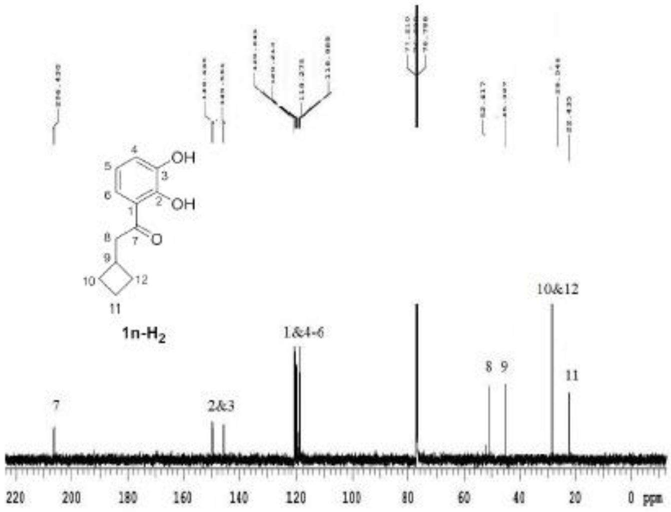

(2,3-dihydroxyphenyl) methylCyclohexyl ketone (1o-H_2): The ligand is prepared from (Bromomethyl)cyclohexane (213 mg, 1.21 mmol) according to the general procedure. Column chromatography (DCM, R_f = 0.23) results in the product as a brown solid (62 %, 175 mg, 0.75 mmol). **M.p.:** 93°C -94.5°C (last solvent: DCM). **^{1}H-NMR** (600 MHz, $CDCl_3$): δ = 12.80 (s, 1H, OH), 7.32 (dd, J = 8.0, 1.5 Hz, 1H, $H_{arom.}$), 7.10 (dd, J = 8.0, 1.5 Hz, 1H, $H_{arom.}$), 6.81 (t, J = 8.0 Hz, 1H, $H_{arom.}$), 5.73 (s, 1H, OH), 3.30-3.26 (m, 2H, CH_2), 1.90-1.86 (m, 4H, H_{cyhex}), 1.78-1.74 (m, 1H, H_{cyhex}), 1.54-1.49 (m, 2H, H_{cyhex}), 1.43-1.35 (m, 2H, H_{cyhex}), 1.31-1.24 (m, 2H, H_{cyhex}) ppm. **^{13}C-NMR** (151 MHz, $CDCl_3$): δ = 210.62 (COCH), 150.25 (C_{arom}), 145.70 (C_{arom}), 120.53 (C_{arom}), 119.90 (C_{arom}), 118.72 (C_{arom}), 118.08 (C_{arom}), 45.42 (CH_2), 30.94 (CH), 29.53 (2×C_{cyhex}), 25.80 (C_{cyhex}), 25.72 (2×C_{cyhex}) ppm. **MS** (negative and positive ESI-MS, MeOH, acidified): m/z (%) = 233.1171 (100, [M-H^+], $C_{14}H_{17}O_3^-$, calcd. 233.1178); 257.1157 (50, [M+Na^+], $C_{14}H_{18}O_3Na^+$, calcd. 257.1154). **IR** (KBr): $\tilde{\upsilon}$ (cm^{-1}) = 3460 , 3040, 2924, 2850, 2660, 2322, 2187, 2111, 1973, 1917, 1785, 1703, 1626, 1488, 1443, 1317, 1272, 1208, 1164, 1141, 1097, 1053, 959, 869, 831, 777, 737, 676. **Elemental Analysis**: $C_{14}H_{18}O_3$: calcd. C = 71.77 %, H = 7.74 %; found C = 71.23 %, H = 7.79 %.

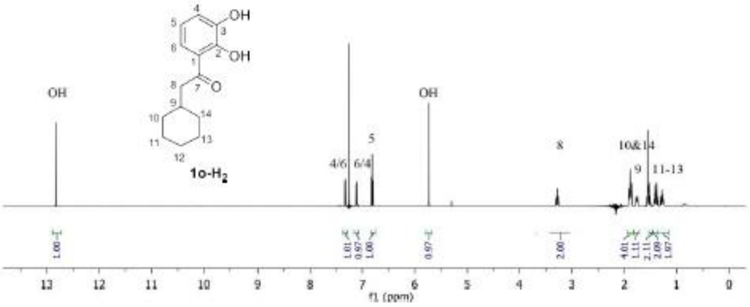

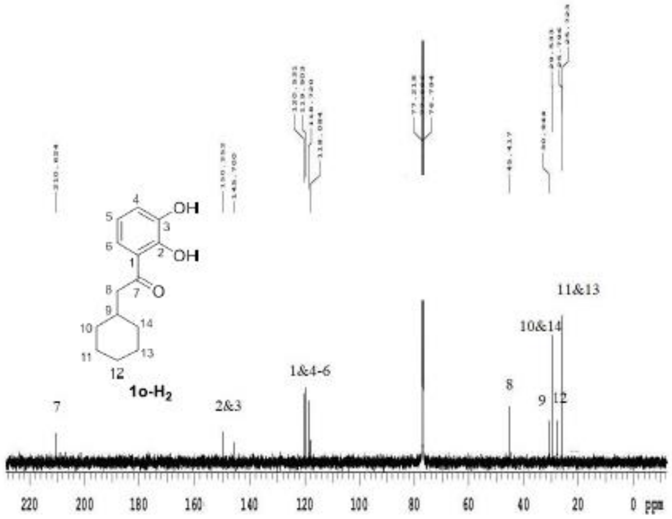

(2,3-dihydroxyphenyl) pentan-3-yl ketone (1q-H_2): The ligand is prepared from 3-Bromopentane (182 mg, 1.21 mmol) according to the general procedure. Column chromatography (DCM, R_f = 0.17) results in the product as a brownish oil (30 %, 74 mg, 0.36 mmol). **^{1}H-NMR** (600 MHz, $CDCl_3$): δ = 12.96 (s, 1H, OH), 7.35 (dd, J = 8.0, 1.5 Hz, 1H, $H_{arom.}$), 7.12 (dd, J = 8.0, 1.5 Hz, 1H, $H_{arom.}$), 6.82 (t, J = 8.0 Hz, 1H, $H_{arom.}$), 5.74 (s, 1H, OH), 3.33-3.30 (m, 1H, CH), 1.83-1.79 (m, 2H, CH_2), 1.62-1.58 (m, 2H, CH_2), 0.90 (t, J = 7.6 Hz, 6H, 2×CH_3) ppm. **^{13}C-NMR** (151 MHz, $CDCl_3$): δ = 211.47 (COCH), 150.09 (C_{arom}), 145.62 (C_{arom}), 120.74 (C_{arom}), 120.04 (C_{arom}), 119.63 (C_{arom}), 118.73 (C_{arom}), 48.71 (CH), 25.10 (2×CH_2), 11.862 (2×CH_3) ppm. **MS** (negative ESI-MS, MeOH, acidified): m/z (%) = 207.1004 (10, [M-H^+], $C_{12}H_{15}O_3^-$, calcd. 207.1021). **IR** (KBr): $\tilde{\nu}$ (cm^{-1}) = 3453, 3057, 2962, 2875, 2664, 2495, 2322, 2094, 2000, 1905, 1630, 1448, 1380, 1321, 1268, 1052, 901, 846, 784, 739. **Elemental Analysis**: $C_{12}H_{16}O_3$ · 175 DCM: calcd. C = 14.90 %, H = 2.45 %; found C =14.91 %, H = 2.51 %.

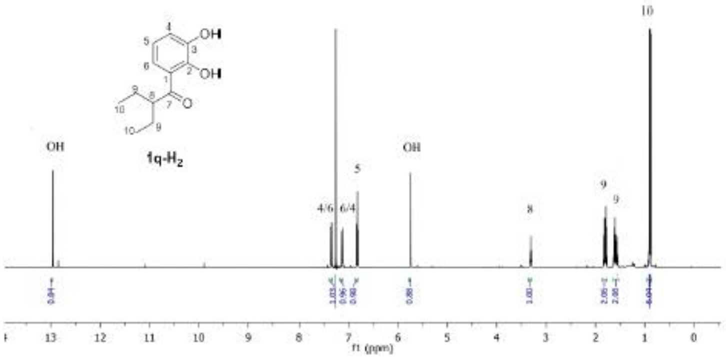

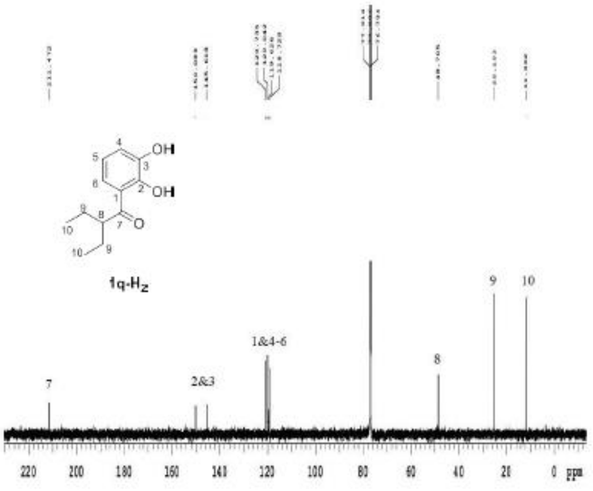

(2,3-dihydroxyphenyl) cyclopentyl ketone (1r-H_2): The ligand is prepared from 1-Bromocyclopentane (180 mg, 1.21 mmol) by modification of the general procedure. Column chromatography (DCM, R_f = 0.18) results in the product as a yellow oil (41 %, 110 mg, 0.49 mmol). **^{1}H-NMR** (600 MHz, $CDCl_3$): δ = 12.76 (s, 1H, OH), 7.34 (dd, J = 8.2, 1.5 Hz, 1H, $H_{arom.}$), 7.11 (dd, J = 8.2, 1.5 Hz, 1H, $H_{arom.}$), 6.81 (t, J = 8.2 Hz, 1H, $H_{arom.}$), 5.73 (s, 1H, OH), 3.76-3.72 (m, 1H, CH), 1.97-1.93 (m, 4H, H_{cypent}), 1.72-1.65 (m, 4H, H_{cypent}) ppm. **^{13}C-NMR** (151 MHz, $CDCl_3$): δ = 209.76 (COCH), 150.02 (C_{arom}), 145.54 (C_{arom}), 120.96 (C_{arom}), 119.79 (C_{arom}), 118.89 (C_{arom}), 118.71 (C_{arom}), 53.42 (CH), 30.90 (2×C_{cypent}), 23.51 (2×C_{cypent}) ppm. **MS** (negative and positive ESI-MS, MeOH, acidified): m/z (%) = 205.0872 (100, [M-H$^+$], $C_{12}H_{13}O_3^-$, calcd. 205.0865); 229.0848 (30, [M+Na$^+$], $C_{12}H_{14}O_3Na^+$, calcd. 229.0841). **IR** (KBr): $\tilde{\upsilon}$ (cm^{-1}) = 3451, 2951, 2868, 2716, 2514, 2325, 2192, 2095, 2011, 1903, 1719, 1632, 1449, 1374, 1319, 1266, 1104, 1049, 931, 853, 744. **Elemental Analysis**: $C_{12}H_{14}O_3 \cdot 1/2\ H_2O$: calcd. C = 66.96 %, H = 7.02 %; found C = 66.87 %, H = 6.73 %.

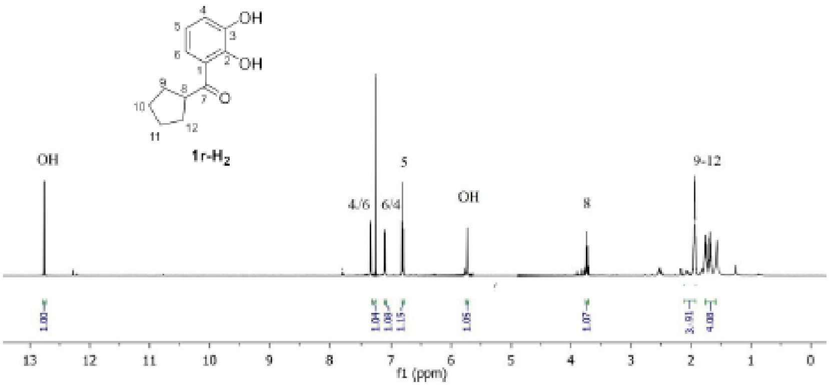

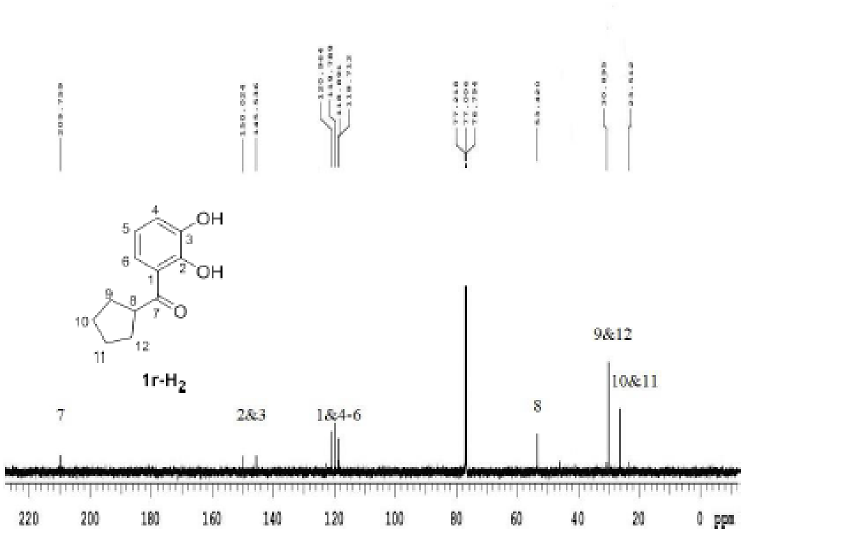

(2,3-dihydroxyphenyl) Cyclohexyl ketone (1s-H_2): The ligand is prepared from 1-Bromocyclohexane (197 mg, 1.21 mmol) according to the general procedure. Column chromatography (DCM, R_f = 0.21) results in the product as a yellow solid (39 %, 105 mg, 0.47 mmol). **M.p.:** 94°C - 95°C (last solvent: DCM). **^{1}H-NMR** (600 MHz, $CDCl_3$): δ = 12.72 (s, 1H, OH), 7.31 (dd, J = 8.1, 1.5 Hz, 1H, $H_{arom.}$), 7.12 (dd, J = 8.1, 1.5 Hz, 1H, $H_{arom.}$), 6.81 (t, J = 8.0 Hz, 1H, $H_{arom.}$), 5.72 (s, 1H, OH), 3.30-3.26 (m, 1H, CH), 1.90-1.86 (m, 3H, H_{cyhex}), 1.78-1.744 (m, 1H, H_{cyhex}), 1.55-1.51 (m, 1H, H_{cyhex}), 1.42-1.38 (m, 2H, H_{cyhex}), 1.29-1.25 (m, 2H, H_{cyhex}), 0.88-0.84 (m, 1H, H_{cyhex}) ppm. **^{13}C-NMR** (151 MHz, $CDCl_3$): δ = 207.28 (COCH), 149.76 (C_{arom}), 145.51 (C_{arom}), 120.97 (C_{arom}), 120.03 (C_{arom}), 119.52 (C_{arom}), 118.78 (C_{arom}), 46.02 (CH), 33.39 (2×C_{cyhex}), 30.94 (C_{cyhex}), 26.15 (2×C_{cyhex}) ppm. **MS** (negative ESI-MS, MeOH, acidified): m/z (%) = 219.1020 (100, [M-H$^+$], $C_{13}H_{15}O_3^-$, calcd. 219.1021). **IR** (KBr): $\tilde{\nu}$ (cm^{-1}) = 3470, 3076, 2922, 2851, 2660, 2295, 2180, 2098, 1973, 1919, 1729, 1630, 1446, 1318, 1275, 1206, 1101, 1046, 968, 898, 826, 779, 732, 675. **Elemental Analysis**: $C_{13}H_{16}O_3$: calcd. C = 70.89 %, H = 7.32 %; found C = 70.92 %, H = 7.51 %.

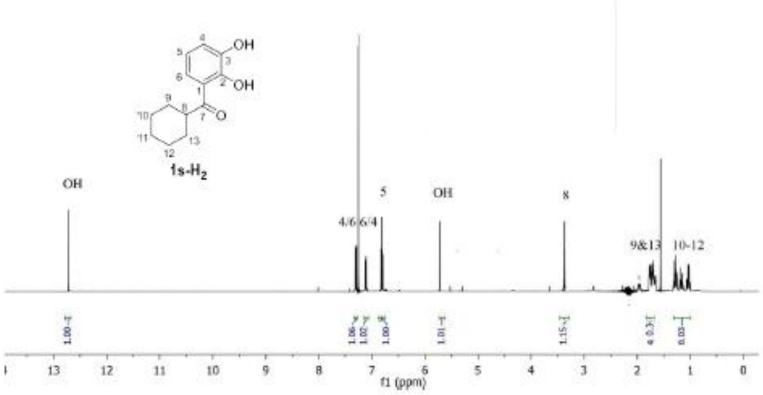

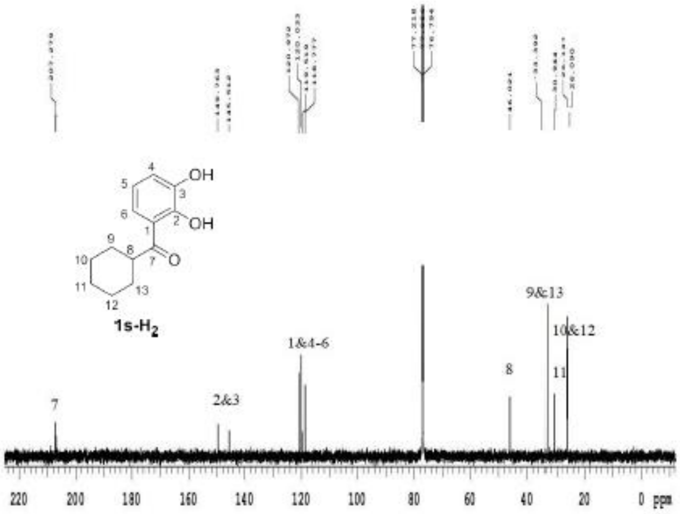

2,3-dihydroxybenzophenone (1t-H_2): The ligand is prepared from Bromobenzene (189 mg, 1.21 mmol) according to the general procedure. Column chromatography (DCM, R_f = 0.12) results in the product as a yellow solid (80 %, 205 mg, 0.96 mmol). **M.p.:** 70°C -72°C (last solvent: DCM). **^{1}H-NMR** (600 MHz, $CDCl_3$): δ = 12.26 (s, 1H, OH), 7.72-7.68 (m, 2H, $H_{arom.}$), 7.61-7.57 (m, 1H, $H_{arom.}$), 7.53-7.49 (m, 2H, $H_{arom.}$), 7.17-7.13 (m, 2H, $H_{arom.}$), 6.83 (t, J = 8.0 Hz, 1H, $H_{arom.}$), 5.78 (s, 1H, OH) ppm. **^{13}C-NMR** (151 MHz, $CDCl_3$): δ = 207.01 (COCH), 150.20 (C_{arom}), 145.55 (C_{arom}), 137.66 (C_{arom}), 132.13 (C_{arom}), 129.22 (2×C_{arom}), 128.34 (2×C_{arom}), 124.48 (C_{arom}), 120.35 (C_{arom}), 119.00 (C_{arom}), 118.72 (C_{arom}) ppm. **MS** (negative and positive ESI-MS, MeOH, acidified): *m/z* (%) = 213.0541 (100, [M-H$^+$], $C_{13}H_9O_3^-$, calcd. 213.0552); 237.0529 (40, [M+Na$^+$], $C_{13}H_{10}O_3Na^+$, calcd. 237.0528). **IR** (KBr): $\tilde{u}$ (cm^{-1}) = 3826, 3326, 3060, 2922, 2657, 2491, 2319, 2211, 2095, 2045, 1985, 1916, 1601, 1441, 1324, 1265, 1224, 1169, 1074, 1011, 930, 862, 811, 742, 694. **Elemental Analysis**: $C_{13}H_{10}O_3$: calcd. C = 72.89 %, H = 4.71 %; found C = 72.77 %, H = 4.96 %.

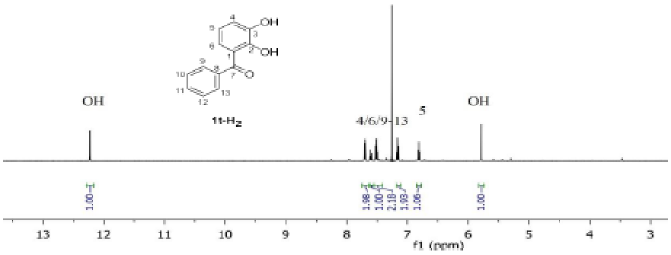

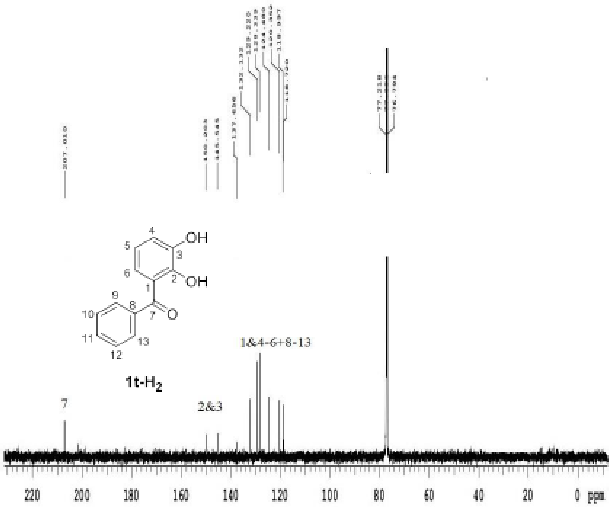

General procedure for the preparation of the complexes: The ligand (3 eq.) is mixed with $TiO(acac)_2$ (1 eq.) and Li_2CO_3 (1 eq.) and dissolved in methanol. The pure complexes are obtained after stirring this solution for one day followed by removal of the solvent.

$Li[Li_3(1a)_3Ti)_2]$: ^{1}H-NMR (400 MHz, Acetone-d_6): Dimer (major component): δ = 7.01 (dd, J = 8.1, 1.6 Hz, 1H, H_{arom}), 6.48 (t, J = 8.1 Hz, 1H, H_{arom}), 6.40 (dd, J = 8.1, 1.6 Hz, 1H, H_{arom}), 1.83 (s, 3H, CH_3) ppm. Monomer (minor component): δ = 7.46 (dd, J = 8.1, 1.6 Hz, 1H, H_{arom}), 7.09 (dd, J = 8.1, 1.6 Hz, 1H, H_{arom}), 6.82 (t, J = 8.1 Hz, 1H, H_{arom}), 1.88 (s, 3H, CH_3) ppm. **^{1}H-NMR** (300 MHz, ACN-d_3): Dimer (major component): δ = 7.08 (dd, J = 8.1, 1.5 Hz, 1H, H_{arom}), 6.59 (t, J = 8.1 Hz, 1H, H_{arom}), 6.54 (dd, J = 8.1, 1.5 Hz, 1H, H_{arom}), 1.87 (s, 3H, CH_3) ppm. Monomer (minor component): δ = 7.42 (dd, J = 8.1, 1.5 Hz, 1H, H_{arom}), 6.82 (t, J = 8.1 Hz, 1H, H_{arom}), 6.78 (dd, J = 8.1, 1.5 Hz, 1H, H_{arom}), 1.91 (s, 3H, CH_3) ppm. **^{1}H-NMR** (600 MHz, MeOH-d_4): Dimer (major component): δ = 7.10 (dd, J = 8.0, 1.5 Hz, 1H, H_{arom}), 6.64-6.55 (m, 2H, H_{arom}), 1.88 (s, 3H, CH_3) ppm. Monomer (minor component): δ = 7.00 (dd, J = 8.0, 1.5 Hz, 1H, H_{arom}), 6.49-6.44 (m, 2H, H_{arom}), 2.61 (s, 3H, CH_3) ppm. **^{1}H-NMR** (600 MHz, DMSO-d_6): Only monomer: δ = 6.73 (dd, J = 8.1, 1.7 Hz, 1H, H_{arom}), 6.25 (t, J = 8.1 Hz, 1H, H_{arom}), 6.13 (dd, J = 8.1, 1.7 Hz, 1H, H_{arom}), 2.49 (s, 3H, CH_3) ppm. **^{1}H-NMR** (600 MHz, THF-d_8): Dimer (major component): δ = 6.95 (dd, J = 7.8, 1.5 Hz, 1H, H_{arom}), 6.47-6.42 (m, 2H, H_{arom}), 1.81 (s, 3H, CH_3) ppm. Monomer (minor component): δ = 7.00 (dd, J = 7.8, 1.5 Hz, 1H, H_{arom}), 6.46 (t, J = 7.8 Hz, 1H, H_{arom}), 6.37 (dd, J = 7.8, 1.5 Hz, 1H, H_{arom}), 2.03 (s, 3H, CH_3) ppm.

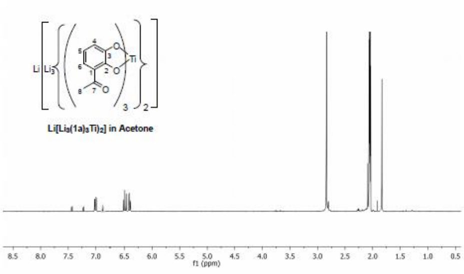

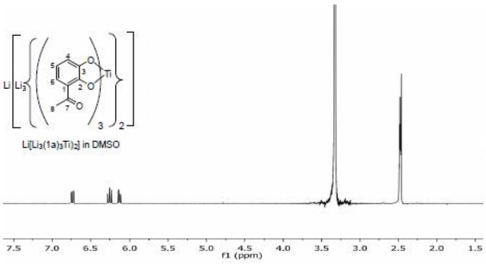

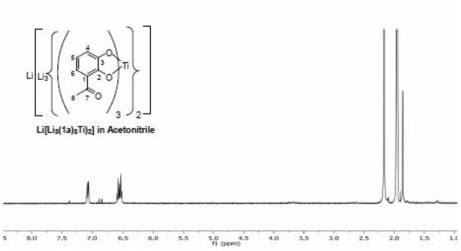

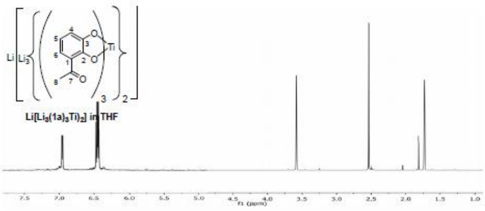

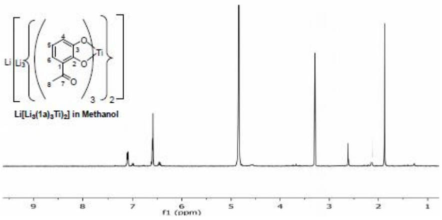

Li[Li$_3$(1b)$_3$Ti)$_2$]: ^{1}H-NMR (400 MHz, Acetone-d$_6$): Dimer (major component): δ = 7.02 (dd, J = 8.2, 1.6 Hz, 1H, H$_{arom}$), 6.47 (t, J = 8.2 Hz, 1H, H$_{arom}$), 6.38 (dd, J = 8.2, 1.6 Hz, 1H, H$_{arom}$), 2.76-2.64 (m, 1H, COCH$_2$), 1.85-1.73 (m, 1H, COCH$_2$), 0.47 (t, J = 6.8 Hz, 3H, CH$_3$) ppm. Monomer (minor component): δ = 7.46 (dd, J = 8.2, 1.6 Hz, 1H, H$_{arom}$), 7.09 (dd, J = 8.2, 1.6 Hz, 1H, H$_{arom}$), 6.82 (t, J = 8.2 Hz, 1H, H$_{arom}$), 3.05 (q, J = 6.8 Hz, 2H, CH$_2$), 0.48 (t, J = 6.8 Hz, 3H, CH$_3$) ppm. **^{1}H-NMR** (600 MHz, ACN-d$_3$): Dimer (major component): δ = 7.10 (dd, J = 8.3, 1.6 Hz, 1H, H$_{arom}$), 6.57 (t, J = 8.3 Hz, 1H, H$_{arom}$), 6.50 (dd, J = 8.3, 1.6 Hz, 1H, H$_{arom}$), 2.80-2.74 (m, 1H, COCH$_2$), 2.14-2.10 (m, 1H, COCH$_2$), 0.49 (t, J = 7.2 Hz, 3H, CH$_3$) ppm. Monomer (minor component): δ = 7.44 (dd, J = 8.3, 1.6 Hz, 1H, H$_{arom}$), 6.84 (t, J = 8.3 Hz, 1H, H$_{arom}$), 6.75 (dd, J = 8.3, 1.6 Hz, 1H, H$_{arom}$), 3.02 (q, J = 7.2 Hz, 2H, CH$_2$), 0.58 (t, J = 7.2 Hz, 3H, CH$_3$) ppm. **^{1}H-NMR** (600 MHz, DMSO-d$_6$): Only monomer: δ = 6.74 (dd, J = 8.1, 1.6 Hz, 1H, H$_{arom}$), 6.25 (t, J = 8.1 Hz, 1H, H$_{arom}$), 6.12 (dd, J = 8.1, 1.6 Hz, 1H, H$_{arom}$), 2.96 (q, J = 7.3 Hz, 2H, CH$_2$), 0.96 (t, J = 7.3 Hz, 3H, CH$_3$) ppm. **^{1}H-NMR** (600 MHz, THF-d$_8$): Dimer (major component): δ = 6.80 (dd, J = 7.9, 1.6 Hz, 1H, H$_{arom}$), 6.28-6.23 (m, 2H, H$_{arom}$), 2.81-2.77 (m, 1H, COCH$_2$), 1.94-1.89 (m, 1H, COCH$_2$), 0.96 (t, J = 7.2 Hz, 3H, CH$_3$) ppm. Monomer (minor component): δ = 6.89 (dd, J = 7.9, 1.6 Hz, 1H, H$_{arom}$), 6.33-6.30 (m, 2H, H$_{arom}$), 3.04 (q, J = 7.2 Hz, 2H, CH$_2$), 1.02 (t, J = 7.2 Hz, 3H, CH$_3$) ppm.

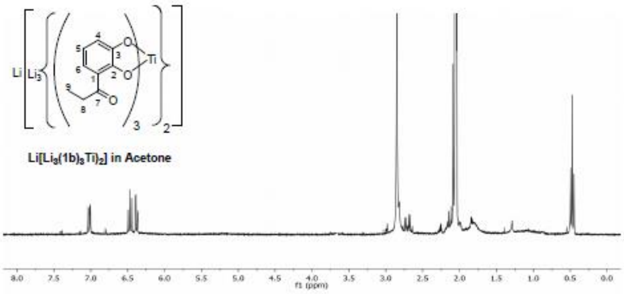

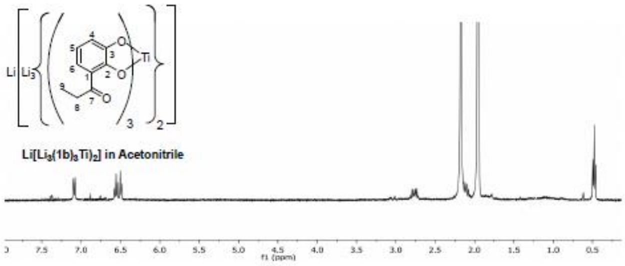

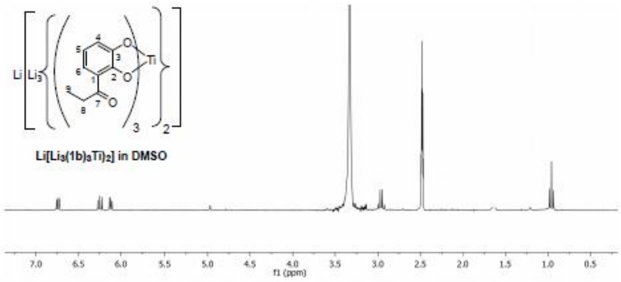

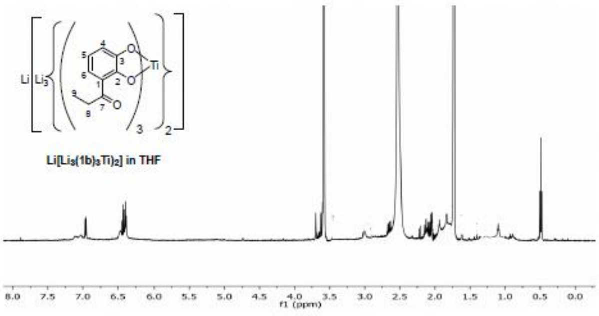

$Li[Li_3(1c)_3Ti)_2]$: **^{1}H-NMR** (300 MHz, Acetone-d_6): Dimer (major component): δ = 7.02 (dd, J = 8.0, 1.5 Hz, 1H, H_{arom}), 6.47 (t, J = 8.0 Hz, 1H, H_{arom}), 6.37 (dd, J = 8.0, 1.5 Hz, 1H, H_{arom}), 2.72-2.64 (m, 1H, $COCH_2$), 1.96-1.88 (m, 1H, $COCH_2$), 0.96-0.84 (m, 2H, CH_2), 0.60 (t, J = 7.4 Hz, 3H, CH_3) ppm. Monomer (minor component): δ = 7.42 (dd, J = 8.0, 1.5 Hz, 1H, H_{arom}), 7.07 (dd, J = 7.8, 1.5 Hz, 1H, H_{arom}), 6.74 (t, J = 8.0 Hz, 1H, H_{arom}), 3.06 (t, J = 6.5 Hz, 2H, CH_2), 1.13-1.04 (m, 2H, CH_2), 0.63 (t, J = 7.4 Hz, 3H, CH_3) ppm. **^{1}H-NMR** (600 MHz, ACN-d_3): Dimer (major component): δ = 7.09 (dd, J = 8.2, 1.5 Hz, 1H, H_{arom}), 6.56 (t, J = 8.2 Hz, 1H, H_{arom}), 6.50 (dd, J = 8.2, 1.5 Hz, 1H, H_{arom}), 2.77-2.69 (m, 1H, $COCH_2$), 1.96-1.90 (m, 1H, $COCH_2$), 0.98-0.86 (m, 2H, CH_2), 0.68 (t, J = 7.4 Hz, 3H, CH_3) ppm. Monomer (minor component): δ = 7.38 (dd, J = 8.2, 1.5 Hz, 1H, H_{arom}), 6.78 (dd, J = 8.2, 1.5 Hz, 1H, H_{arom}), 6.68 (t, J = 8.2 Hz, 1H, H_{arom}), 3.02 (t, J = 6.5 Hz, 2H, CH_2), 1.13-1.07 (m, 2H, CH_2), 0.70 (t, J = 7.4 Hz, 3H, CH_2) ppm. **^{1}H-NMR** (600 MHz, DMSO-d_6): Dimer (minor component): δ = 7.02 (dd, J = 8.2, 1.5 Hz, 1H, H_{arom}), 6.45 (t, J = 8.2 Hz, 1H, H_{arom}), 6.36 (dd, J = 8.2, 1.5 Hz, 1H, H_{arom}), 2.66-2.62 (m, 1H, $COCH_2$), 1.80-1.74 (m, 1H, $COCH_2$), 1.23-1.19 (m, 2H, CH_2), 0.80 (t, J = 7.4 Hz, 3H, CH_3) ppm. Monomer (major component): δ = 6.75 (dd, J = 8.2, 1.5 Hz, 1H, H_{arom}), 6.24 (t, J = 8.2 Hz, 1H, H_{arom}), 6.12 (dd, J = 8.2, 1.5 Hz, 1H, H_{arom}), 2.94 (t, J = 7.2 Hz, 2H, CH_2), 1.54-1.48 (m, 2H, CH_2), 0.82 (t, J = 7.4 Hz, 3H, CH_3) ppm. **^{1}H-NMR** (600 MHz, THF-d_8): Dimer (major component): δ = 6.83 (dd, J = 7.8, 1.5 Hz, 1H, H_{arom}), 6.31-6.25 (m, 2H, H_{arom}), 2.52-2.46 (m, 1H, $COCH_2$), 1.80-1.75 (m, 1H, $COCH_2$), 0.83-0.77 (m, 2H, CH_2), 0.50 (t, J = 7.4 Hz, 3H, CH_3) ppm. Monomer (minor component): δ = 6.88 (dd, J = 7.8, 1.5 Hz, 1H, H_{arom}), 6.31(t, J = 7.8 Hz, 1H, H_{arom}), 6.22 (dd, J = 7.8, 1.5 Hz, 1H, H_{arom}), 2.76 (t, J = 6.5 Hz, 2H, CH_2), 1.01-0.95 (m, 2H, CH_2), 0.85 (t, J = 7.4 Hz, 3H, CH_3) ppm.

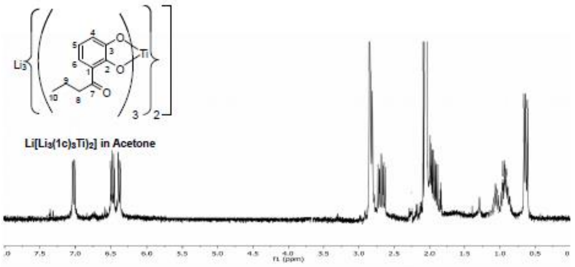

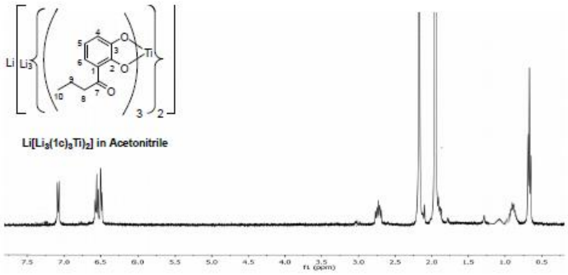

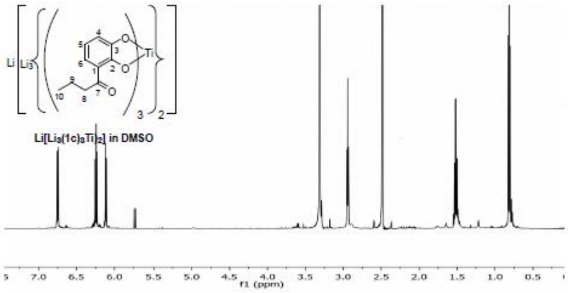

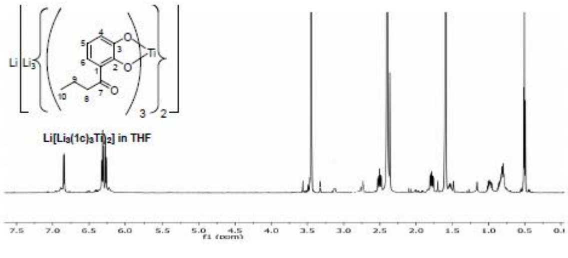

Li[Li$_3$(1d)$_3$Ti)$_2$]: ^{1}H-NMR (300 MHz, Acetone-d$_6$): Dimer (major component): δ = 7.03 (dd, J = 7.8, 1.5 Hz, 1H, H$_{arom}$), 6.47 (t, J = 7.8 Hz, 1H, H$_{arom}$), 6.39 (dd, J = 7.8, 1.5 Hz, 1H, H$_{arom}$), 2.77-2.66 (m, 1H, COCH$_2$), 1.99-1.89 (m, 1H, COCH$_2$), 1.12-1.03 (m, 2H, CH$_2$), 0.95-0.83 (m, 2H, CH$_2$), 0.71 (t, J = 7.1 Hz, 3H, CH$_3$) ppm. Monomer (minor component): δ = 7.45 (dd, J = 7.8, 1.5 Hz, 1H, H$_{arom}$), 7.09 (dd, J = 7.8, 1.5 Hz, 1H, H$_{arom}$), 6.82 (t, J = 7.8 Hz, 1H, H$_{arom}$), 3.09 (t, J = 6.8 Hz, 2H, CH$_2$), 1.75-1.68 (m, 2H, CH$_2$), 1.46-1.39 (m, 2H, CH$_2$), 0.75 (t, J = 7.1 Hz, 3H, CH$_3$) ppm. **^{1}H-NMR** (600 MHz, ACN-d$_3$): Dimer (major component): δ = 7.09 (dd, J = 8.1, 1.5 Hz, 1H, H$_{arom}$), 6.56 (t, J = 8.1 Hz, 1H, H$_{arom}$), 6.51 (dd, J = 8.1, 1.5 Hz, 1H, H$_{arom}$), 2.79-2.75 (m, 1H, COCH$_2$), 1.94-1.90 (m, 1H, COCH$_2$), 1.14-1.05 (m, 4H, 2× CH$_2$), 0.77 (t, J = 7.1 Hz, 3H, CH$_3$) ppm. Monomer (minor component): δ = 7.46 (dd, J = 8.1, 1.5 Hz, 1H, H$_{arom}$), 6.84 (t, J = 8.1 Hz, 1H, H$_{arom}$), 6.68 (dd, J = 8.1, 1.5 Hz, 1H, H$_{arom}$), 3.07 (t, J = 6.8 Hz, 2H, CH$_2$), 1.71-1.69 (m, 2H, CH$_2$), 1.44-1.39 (m, 2H, CH$_2$), 0.96 (t, J = 7.1 Hz, 3H, CH$_3$) ppm. **^{1}H-NMR** (600 MHz, DMSO-d$_6$): Dimer (minor component): δ = 7.02 (dd, J = 8.0, 1.6 Hz, 1H, H$_{arom}$), 6.47 (t, J = 8.0 Hz, 1H, H$_{arom}$), 6.37 (dd, J = 8.0, 1.6 Hz, 1H, H$_{arom}$), 2.70-2.66 (m, 1H, COCH$_2$), 1.83-1.75 (m, 1H, COCH$_2$), 0.85-0.78 (m, 4H, 2× CH$_2$), 0.63 (t, J = 7.4 Hz, 3H, CH$_3$) ppm. Monomer (major component): δ = 6.74 (dd, J = 8.0, 1.6 Hz, 1H, H$_{arom}$), 6.23 (t, J = 8.0 Hz, 1H, H$_{arom}$), 6.10 (dd, J = 8.0, 1.6 Hz, 1H, H$_{arom}$), 2.96 (t, J = 7.3 Hz, 2H, CH$_2$), 1.48-1.42 (m, 2H, CH$_2$), 1.24-1.19 (m, 2H, CH$_2$), 0.75 (t, J = 7.4 Hz, 3H, CH$_3$) ppm. **^{1}H-NMR** (600 MHz, THF-d$_8$): Dimer (major component): δ = 6.84 (dd, J = 7.8, 1.5 Hz, 1H, H$_{arom}$), 6.31-6.26 (m, 2H, H$_{arom}$), 2.57-2.51 (m, 1H, COCH$_2$), 1.79-1.74 (m, 1H, COCH$_2$), 0.97-0.92 (m, 2H, CH$_2$), 0.79-0.74 (m, 2H, CH$_2$), 0.58 (t, J = 7.2 Hz, 3H, CH$_3$) ppm. Monomer (minor component): δ = 6.87 (dd, J = 7.8, 1.5 Hz, 1H, H$_{arom}$), 6.34 (t, J = 7.8 Hz, 1H, H$_{arom}$), 6.21 (dd, J = 7.8, 1.5 Hz, 1H, H$_{arom}$), 2.89 (t, J = 6.5 Hz, 2H, CH$_2$), 1.30-1.25 (m, 2H, CH$_2$), 1.22-1.19 (m, 2H, CH$_2$), 0.82 (t, J = 7.2 Hz, 3H, CH$_3$) ppm.

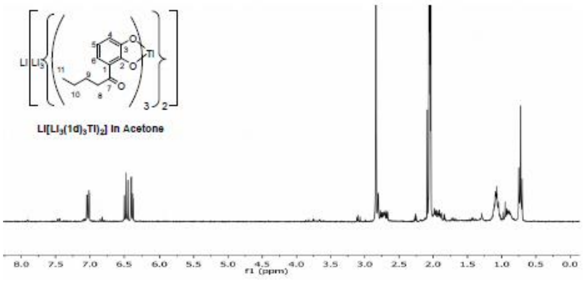

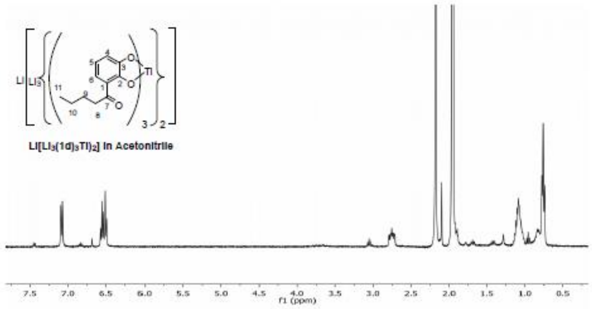

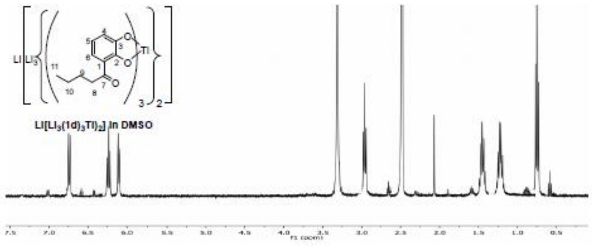

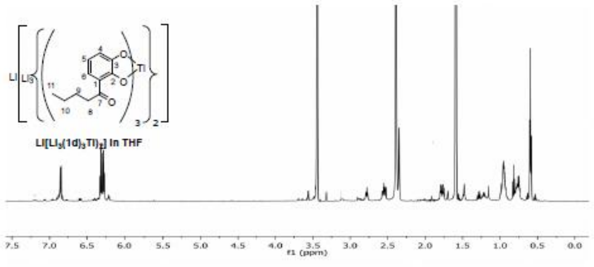

Li[Li$_3$(1e)$_3$Ti)$_2$]: **^{1}H-NMR** (600 MHz, Acetone-d$_6$): Dimer (major component): δ = 7.03 (dd, J = 7.9, 1.5 Hz, 1H, H_{arom}), 6.47 (t, J = 7.9 Hz, 1H, H_{arom}), 6.38 (dd, J = 7.9, 1.5 Hz, 1H, H_{arom}), 2.72-2.65 (m, 1H, $COCH_2$), 1.99-1.91 (m, 1H, $COCH_2$), 1.16-1.01 (m, 6H), 0.77 (t, J = 7.0 Hz, 3H, CH_3) ppm. Monomer (minor component): δ = 7.40 (dd, J = 7.9, 1.5 Hz, 1H, H_{arom}), 7.09 (dd, J = 7.9, 1.5 Hz, 1H, H_{arom}), 6.78 (t, J = 7.9 Hz, 1H, H_{arom}), 3.05 (t, J = 6.8 Hz, 2H, CH_2), 1.31-1.28 (m, 6H), 0.91 (t, J = 7.0 Hz, 3H, CH_3) ppm. **^{1}H-NMR** (600 MHz, ACN-d$_3$): Dimer (major component): δ = 7.08 (dd, J = 8.3, 1.5 Hz, 1H, H_{arom}), 6.55 (t, J = 8.3 Hz, 1H, H_{arom}), 6.50 (dd, J = 8.3, 1.5 Hz, 1H, H_{arom}), 2.78-2.70 (m, 1H, CH_2), 1.97-1.91 (m, 1H, CH_2), 1.11-1.04 (m, 4H, 2× CH_2), 0.90-0.84 (m, 2H, CH_2), 0.80 (t, J = 7.3 Hz, 3H, CH_3) ppm. Monomer (minor component): δ = 7.44 (dd, J = 8.3, 1.5 Hz, 1H, H_{arom}), 6.84 (t, J = 8.3 Hz, 1H, H_{arom}), 6.70 (dd, J = 8.3, 1.5 Hz, 1H, H_{arom}), 3.05 (t, J = 6.8 Hz, 2H, CH_2), 1.21-1.14 (m, 6H), 0.84 (t, J = 7.3 Hz, 3H, CH_3) ppm. **^{1}H-NMR** (600 MHz, DMSO-d$_6$): Dimer (minor component): δ = 7.09 (dd, J = 8.1, 1.6 Hz, 1H, H_{arom}), 6.46 (t, J = 8.1 Hz, 1H, H_{arom}), 6.35 (dd, J = 8.1, 1.6 Hz, 1H, H_{arom}), 2.70-2.62 (m, 1H, $COCH_2$), 1.81-1.72 (m, 1H, $COCH_2$), 1.10-1.04 (m, 2H, CH_2), 0.86-0.83 (m, 4H, 2× CH_2), 0.65 (t, J = 7.4 Hz, 3H, CH_3) ppm. Monomer (major component): δ = 6.74 (dd, J = 8.1, 1.6 Hz, 1H, H_{arom}), 6.23 (t, J = 8.1 Hz, 1H, H_{arom}), 6.10 (dd, J = 8.1, 1.6 Hz, 1H, H_{arom}), 2.96 (t, J = 7.4 Hz, 2H, CH_2), 1.50-1.44 (m, 2H, CH_2), 1.20-1.13 (m, 4H, 2× CH_2), 0.74 (t, J = 7.4 Hz, 3H, CH_3) ppm. **^{1}H-NMR** (600 MHz, THF-d$_8$): Dimer (major component): δ = 6.97 (dd, J = 7.8, 1.5 Hz, 1H, H_{arom}), 6.44-6.39 (m, 2H, H_{arom}), 2.68-2.63 (m, 1H, $COCH_2$), 1.94-1.88 (m, 1H, $COCH_2$), 0.96-0.90 (m, 2H, CH_2), 0.77 (t, J = 7.3 Hz, 3H. CH_3) ppm. Monomer (minor component): δ = 7.01 (dd, J = 7.8, 1.5 Hz, 1H, H_{arom}), 6.36 (dd, J = 7.8, 1.5 Hz, 1H, H_{arom}), 2.91 (t, J = 6.5 Hz, 2H, CH_2), 1.33-1.29 (m, 2H, CH_2), 0.87 (t, J = 7.3 Hz, 3H, CH_3) ppm. Signals not listed are overlapping and cannot be assigned.

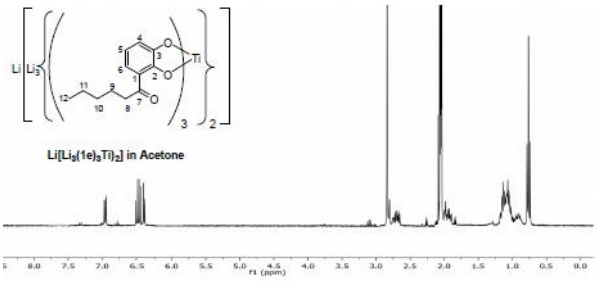

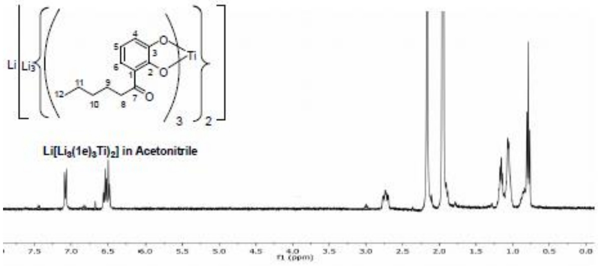

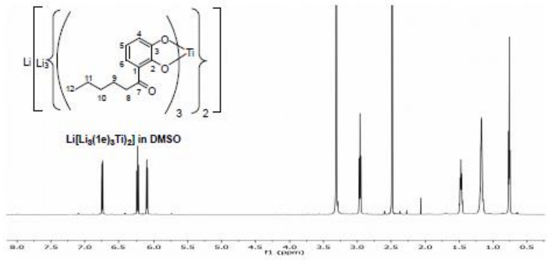

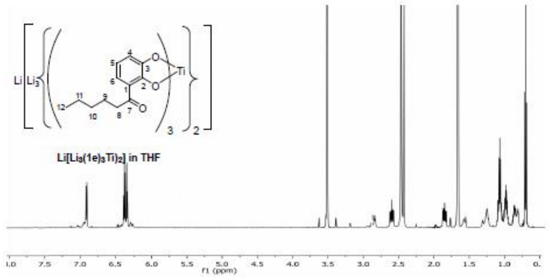

$Li[Li_3(1f)_3Ti)_2]$: ^{1}H-NMR (600 MHz, Acetone-d_6): Dimer (major component): δ = 7.02 (dd, J = 8.0, 1.5 Hz, 1H, H_{arom}), 6.47 (t, J = 8.0 Hz, 1H, H_{arom}), 6.39 (dd, J = 8.0, 1.5 Hz, 1H, H_{arom}), 2.76-2.67 (m, 1H, $COCH_2$), 2.00-1.90 (m, 1H, $COCH_2$), 1.21-1.06 (m, 8H), 0.78 (t, J = 7.4 Hz, 3H, CH_3) ppm. Monomer (minor component): δ = 7.42 (dd, J = 8.0, 1.5 Hz, 1H, H_{arom}), 7.06 (dd, J = 8.0, 1.5 Hz, 1H, H_{arom}), 6.77 (t, J = 8.0 Hz, 1H, H_{arom}), 3.08 (t, J = 7.0 Hz, 2H, CH_2), 1.34-1.28 (m, 8H), 0.90 (t, J = 7.4 Hz, 3H, CH_3) ppm. **^{1}H-NMR** (600 MHz, ACN-d_3): Dimer (major component): δ = 7.08 (dd, J = 7.8, 1.5 Hz, 1H, H_{arom}), 6.55 (t, J = 7.8 Hz, 1H, H_{arom}), 6.50 (dd, J = 7.8, 1.5 Hz, 1H, H_{arom}), 2.77-2.71 (m, 1H, $COCH_2$), 1.97-1.88 (m, 1H, $COCH_2$), 1.21-1.18 (m, 2H; CH_2), 1.16-1.07 (m, 6H), 0.83 (t, J = 7.2 Hz, 3H, CH_3) ppm. Monomer (minor component): δ = 7.44 (dd, J = 7.8, 1.5 Hz, 1H, H_{arom}), 6.83 (t, J = 7.8 Hz, 1H, H_{arom}), 6.75 (dd, J = 7.8, 1.5 Hz, 1H, H_{arom}), 3.05 (t, J = 7.0 Hz, 2H, CH_2), 1.37-1.29 (m, 6H), 0.94-0.90 (m, 2H, CH_2), 0.85 (t, J = 7.2 Hz, 3H, CH_3) ppm. **^{1}H-NMR** (600 MHz, DMSO-d_6): Dimer (minor component): δ = 7.01 (dd, J = 7.9, 1.6 Hz, 1H, H_{arom}), 6.47 (t, J = 7.9 Hz, 1H, H_{arom}), 6.37 (dd, J = 7.9, 1.6 Hz, 1H, H_{arom}), 2.71-2.60 (m, 1H, $COCH_2$), 1.80-1.69 (m, 1H, $COCH_2$) ppm. Monomer (major component): δ = 6.73 (dd, J = 7.9, 1.6 Hz, 1H, H_{arom}), 6.22 (t, J = 7.9 Hz, 1H, H_{arom}), 6.09 (dd, J = 7.9, 1.6 Hz, 1H, H_{arom}), 2.95 (t, J = 7.3 Hz, 2H, CH_2) ppm. Signals not listed are overlapping and cannot be assigned. **^{1}H-NMR** (600 MHz, THF-d_8): Dimer (major component): δ = 6.97 (dd, J = 7.8, 1.6 Hz, 1H, H_{arom}), 6.443-6.39 (m, 2H, H_{arom}), 2.69-2.63 (m, 1H, $COCH_2$), 1.94-1.89 (m, 1H, $COCH_2$), 0.80 (t, J = 7.2 Hz, 3H, CH_3) ppm. Monomer (minor component): δ = 7.03 (dd, J = 7.8, 1.6 Hz, 1H, H_{arom}), 6.39 (dd, J = 7.8, 1.6 Hz, 1H, H_{arom}), 2.91 (t, J = 6.7 Hz, 2H, CH_2) ppm. Signals not listed are overlapping and cannot be assigned.

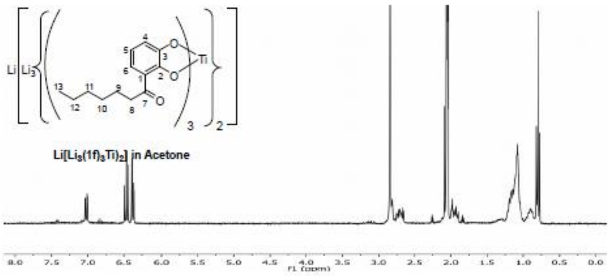

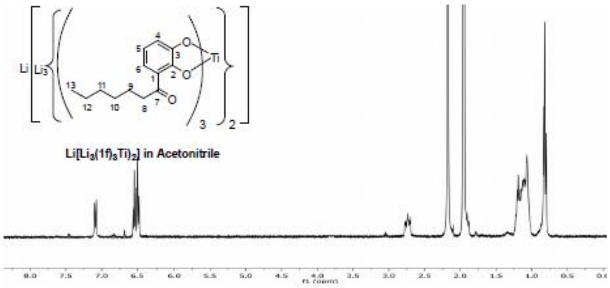

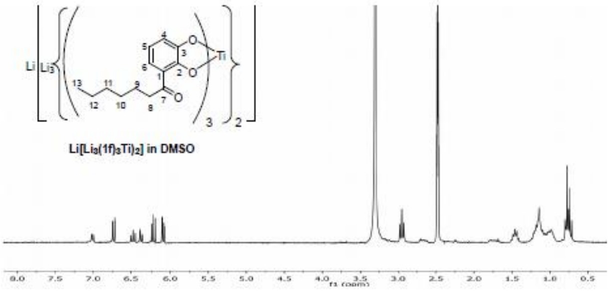

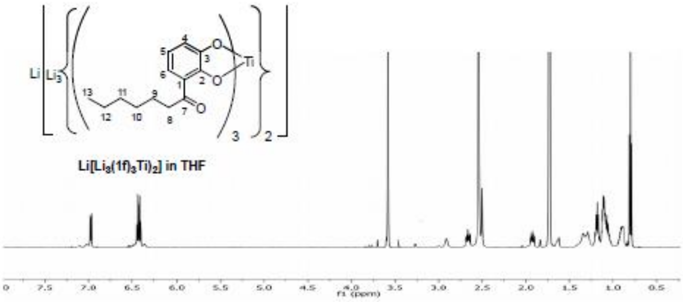

Li[Li$_3$(1g)$_3$Ti)$_2$]: ^{1}H-NMR (600 MHz, Acetone-d$_6$): Dimer (major component): δ = 7.02 (dd, J = 8.2, 1.6 Hz, 1H, H_{arom}), 6.46 (t, J = 8.2 Hz, 1H, H_{arom}), 6.38 (dd, J = 8.2, 1.6 Hz, 1H, H_{arom}), 2.75-2.64 (m, 1H, $COCH_2$), 2.01-1.89 (m, 1H, $COCH_2$), 1.24-1.02 (m, 10H), 0.82 (t, J = 6.8 Hz, 3H, CH_3) ppm. Monomer (minor component): δ = 7.46 (dd, J = 8.2, 1.6 Hz, 1H, H_{arom}), 7.09 (dd, J = 8.2, 1.6 Hz, 1H, H_{arom}), 6.82 (t, J = 8.2 Hz, 1H, H_{arom}), 3.08 (t, J = 6.5 Hz, 2H, CH_2), 1.43-1.25 (m, 10H), 0.89 (t, J = 6.8 Hz, 3H, CH_3) ppm. **^{1}H-NMR** (600 MHz, ACN-d$_3$): Dimer (major component): δ = 7.08 (dd, J = 7.8, 1.6 Hz, 1H, H_{arom}), 6.55 (t, J = 7.8 Hz, 1H, H_{arom}), 6.50 (dd, J = 7.8, 1.6 Hz, 1H, H_{arom}), 2.76-2.71 (m, 1H, $COCH_2$), 1.96-1.89 (m, 1H, $COCH_2$), 1.26-1.21 (m, 2H, CH_2), 1.18-1.13 (m, 6H), 1.11-1.04 (m, 2H, CH_2), 0.86 (t, J = 7.3 Hz, 3H, CH_3) ppm. Monomer (minor component): δ = 7.44 (dd, J = 7.8, 1.6 Hz, 1H, H_{arom}), 6.84 (t, J = 7.8 Hz, 1H, H_{arom}), 6.69 (dd, J = 7.8, 1.6 Hz, 1H, H_{arom}), 3.05 (t, J = 6.5 Hz, 2H, CH_2), 1.71-1.69 (m, 2H), 1.41-1.35 (m, 6H), 1.32-1.29 (m, 2H, CH_2), 0.91 (t, J = 7.3 Hz, 3H, CH_3) ppm. **^{1}H-NMR** (600 MHz, DMSO-d$_6$): Dimer (minor component): δ = 7.01 (dd, J = 7.9, 1.5 Hz, 1H, H_{arom}), 6.48 (t, J = 7.9 Hz, 1H, H_{arom}), 6.38 (dd, J = 7.9, 1.5 Hz, 1H, H_{arom}), 2.72-2.60 (m, 1H, $COCH_2$), 1.79-1.70 (m, 1H, $COCH_2$) ppm. Monomer (major component): δ = 6.73 (dd, J = 7.9, 1.5 Hz, 1H, H_{arom}), 6.23 (t, J = 7.9 Hz, 1H, H_{arom}), 6.09 (dd, J = 7.9, 1.5 Hz, 1H, H_{arom}), 2.96 (t, J = 7.3 Hz, 2H, CH_2) ppm. Signals not listed are overlapping and cannot be assigned. **^{1}H-NMR** (600 MHz, THF-d$_8$): Dimer (major component): δ = 6.96 (dd, J = 7.9, 1.5 Hz, 1H, H_{arom}), 6.44-6.39 (m, 2H, H_{arom}), 2.69-2.63 (m, 1H, $COCH_2$), 1.94-1.89 (m, 1H, $COCH_2$), 0.83 (t, J = 7.2 Hz, 3H, CH_3) ppm. Monomer (minor component): δ = 7.01 (dd, J = 7.9, 1.5 Hz, 1H, H_{arom}), 6.35 (dd, J = 7.9, 1.5 Hz, 1H, H_{arom}), 2.91 (t, J = 6.8 Hz, 2H, CH_2) ppm. Signals not listed are overlapping and cannot be assigned.

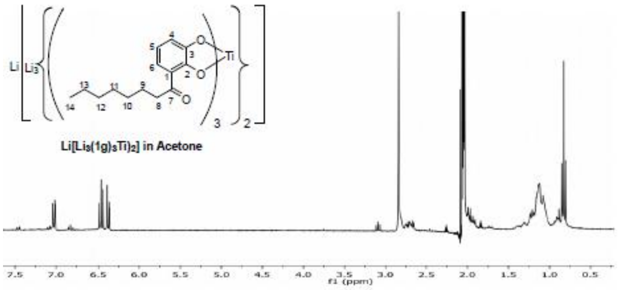

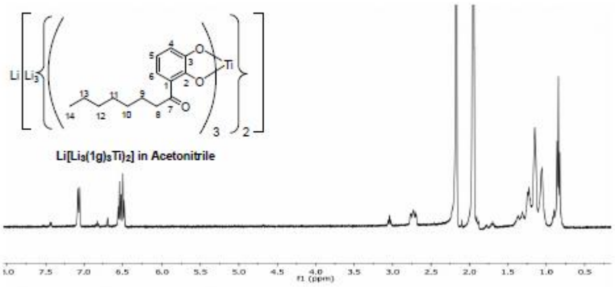

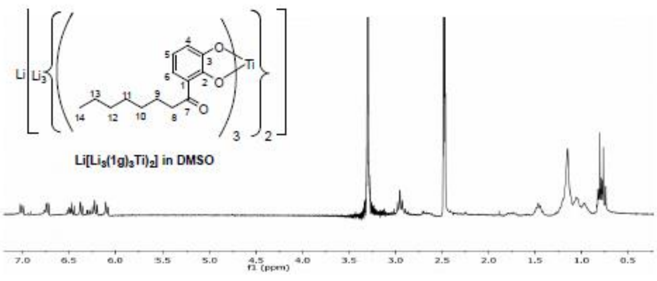

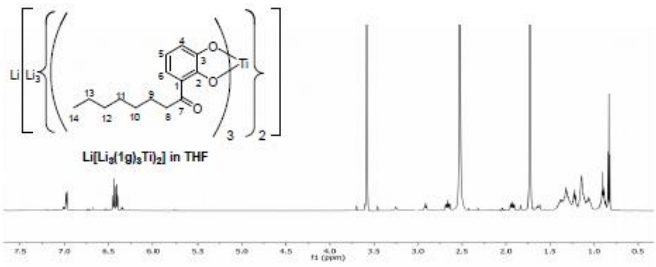

$Li[Li_3(1h)_3Ti)_2]$: ^{1}**H-NMR** (600 MHz, Acetone-d_6): Dimer (major component): δ = 7.02 (dd, J = 8.0, 1.5 Hz, 1H, H_{arom}), 6.46 (t, J = 8.0 Hz, 1H, H_{arom}), 6.38 (dd, J = 8.0, 1.5 Hz, 1H, H_{arom}), 2.76-2.65 (m, 1H, $COCH_2$), 2.02-1.88 (m, 1H, $COCH_2$), 1.24-1.02 (m, 12H), 0.83 (t, J = 6.9 Hz, 3H, CH_3) ppm. Monomer (minor component): δ = 7.46 (dd, J = 8.0, 1.5 Hz, 1H, H_{arom}), 7.06 (dd, J = 8.0, 1.5 Hz, 1H, H_{arom}), 6.78 (t, J = 8.0 Hz, 1H, H_{arom}), 3.04 (t, J = 6.5 Hz, 2H, CH_2), 1.32-1.26 (m, 12H), 0.93 (t, J = 6.9 Hz, 3H, CH_3) ppm. ^{1}**H-NMR** (600 MHz, ACN-d_3): Dimer (major component): δ = 7.08 (dd, J = 7.9, 1.5 Hz, 1H, H_{arom}), 6.55 (t, J = 7.9 Hz, 1H, H_{arom}), 6.49 (dd, J = 7.9, 1.5 Hz, 1H, H_{arom}), 2.75-2.70 (m, 1H, $COCH_2$), 1.95-1.88 (m, 1H, $COCH_2$), 1.30-1.26 (m, 2H, CH_2), 1.23-1.13 (m, 6H), 1.11-1.05 (m, 4H), 0.87 (t, J = 7.2 Hz, 3H, CH_3) ppm. Monomer (minor component): δ = 7.40 (dd, J = 7.9, 1.5 Hz, 1H, H_{arom}), 6.82 (t, J = 7.9 Hz, 1H, H_{arom}), 6.78 (dd, J = 7.9, 1.5 Hz, 1H, H_{arom}), 3.02 (t, J = 6.8 Hz, 2H, CH_2), 0.91 (t, J = 7.2 Hz, 3H, CH_3) ppm. Signals not listed are overlapping and cannot be assigned. ^{1}**H-NMR** (600 MHz, DMSO-d_6): Dimer (minor component): δ = 7.00 (dd, J = 8.0, 1.5 Hz, 1H, H_{arom}), 6.47 (t, J = 8.0 Hz, 1H, H_{arom}), 6.36 (dd, J = 8.0, 1.5 Hz, 1H, H_{arom}), 2.71-2.58 (m, 1H, $COCH_2$), 1.78-1.70 (m, 1H, $COCH_2$) ppm. Monomer (major component): δ = 6.73 (dd, J = 8.0, 1.5 Hz, 1H, H_{arom}), 6.22 (t, J = 8.0 Hz, 1H, H_{arom}), 6.09 (dd, J = 8.0, 1.5 Hz, 1H, H_{arom}), 2.95 (t, J = 7.3 Hz, 2H, CH_2) ppm. Signals not listed are overlapping and cannot be assigned. ^{1}**H-NMR** (600 MHz, THF-d_8): Dimer (major component): δ = 6.97 (dd, J = 7.8, 1.5 Hz, 1H, H_{arom}), 6.44-6.39 (m, 2H, H_{arom}), 2.69-2.63 (m, 1H, $COCH_2$), 1.94-1.88 (m, 1H, $COCH_2$), 0.85 (t, J = 7.3 Hz, 3H, CH_3) ppm. Monomer (minor component): δ = 7.02 (dd, J = 7.8, 1.5 Hz, 1H, H_{arom}), 6.36 (dd, J = 7.8, 1.5 Hz, 1H, H_{arom}), 2.91 (t, J = 6.5 Hz, 2H, CH_2), 0.88 (t, J = 7.3 Hz, 3H, CH_3) ppm. Signals not listed are overlapping and cannot be assigned.

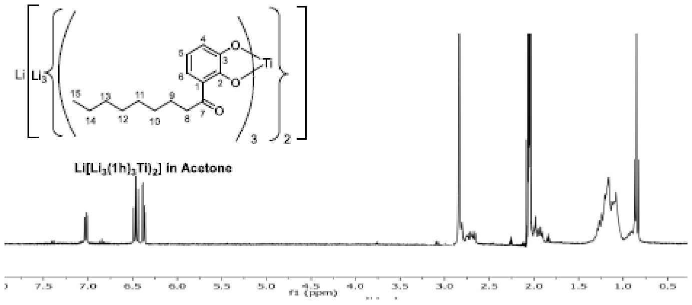

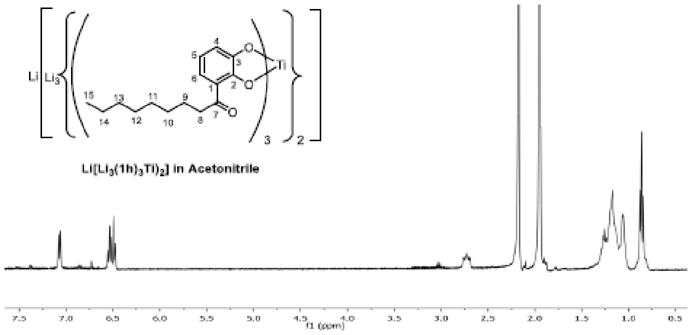

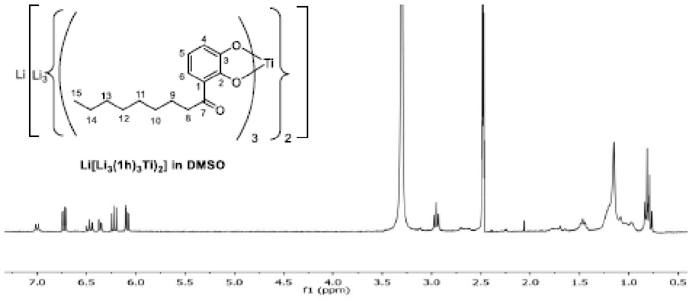

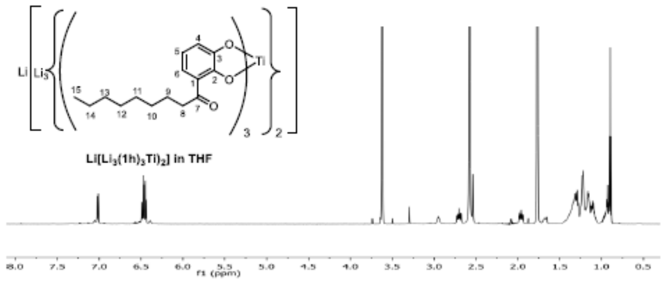

$Li[Li_3(1i)_3Ti)_2]$: ^{1}H-NMR (600 MHz, Acetone-d_6): Dimer (major component): δ = 7.02 (dd, J = 8.0, 1.6 Hz, 1H, H_{arom}), 6.47 (t, J = 8.0 Hz, 1H, H_{arom}), 6.38 (dd, J = 8.0, 1.6 Hz, 1H, H_{arom}), 2.75-2.64 (m, 1H, $COCH_2$), 1.97-1.88 (m, 1H, $COCH_2$), 1.26-1.03 (m, 14H), 0.87 (t, J = 6.8 Hz, 3H, CH_3) ppm. Monomer (minor component): δ = 7.47 (dd, J = 8.0, 1.6 Hz, 1H, H_{arom}), 7.07 (dd, J = 8.0, 1.6 Hz, 1H, H_{arom}), 6.77 (t, J = 8.0 Hz, 1H, H_{arom}), 3.06 (t, J = 6.7 Hz, 2H, CH_2), 1.37-1.28 (m, 14H), 0.93 (t, J = 6.8 Hz, 3H, CH_3) ppm. **^{1}H-NMR** (600 MHz, ACN-d_3): Dimer (major component): δ = 7.07 (dd, J = 7.8, 1.6 Hz, 1H, H_{arom}), 6.54 (t, J = 7.8 Hz, 1H, H_{arom}), 6.49 (dd, J = 7.8, 1.6 Hz, 1H, H_{arom}), 2.77-2.70 (m, 1H, $COCH_2$), 1.95-1.89 (m, 1H, $COCH_2$), 1.32-1.27 (m, 4H) ppm. Monomer (minor component): δ = 7.42 (dd, J = 7.8, 1.6 Hz, 1H, H_{arom}), 6.84 (t, J = 7.8 Hz, 1H, H_{arom}), 6.76 (dd, J = 7.8, 1.6 Hz, 1H, H_{arom}), 3.02 (t, J = 6.8 Hz, 2H, CH_2) ppm. Signals not listed are overlapping and cannot be assigned. **^{1}H-NMR** (600 MHz, MeOH-d_4): Dimer (major component): δ = 7.10 (dd, J = 7.9, 1.5 Hz, 1H, H_{arom}), 6.59-6.56 (m, 2H, H_{arom}), 2.73-2.67 (m, 1H, $COCH_2$), 2.00-1.94 (m, 1H, $COCH_2$) ppm. Monomer (minor component): δ = 6.97 (dd, J = 7.9, 1.5 Hz, 1H, H_{arom}), 6.47-6.39 (m, 2H, H_{arom}), 3.07 (t, J = 6.8 Hz, 2H, CH_2) ppm. Signals not listed are overlapping and cannot be assigned. **^{1}H-NMR** (600 MHz, DMSO-d_6): Dimer (minor component): δ = 7.00 (dd, J = 8.1, 1.6 Hz, 1H, H_{arom}), 6.48 (t, J = 8.1 Hz, 1H, H_{arom}), 6.36 (dd, J = 8.1, 1.6 Hz, 1H, H_{arom}), 2.72-2.60 (m, 1H, $COCH_2$), 1.78-1.69 (m, 1H, $COCH_2$) ppm. Monomer (major component): δ = 6.73 (dd, J = 8.1, 1.6 Hz, 1H, H_{arom}), 6.22 (t, J = 8.1 Hz, 1H, H_{arom}), 6.09 (dd, J = 8.1, 1.6 Hz, 1H, H_{arom}), 2.95 (t, J = 7.3 Hz, 2H, CH_2) ppm. Signals not listed are overlapping and cannot be assigned. **^{1}H-NMR** (600 MHz, THF-d_8): Dimer (major component): δ = 6.96 (dd, J = 7.9, 1.5 Hz, 1H, H_{arom}), 6.44-6.38 (m, 2H, H_{arom}), 2.69-2.63 (m, 1H, $COCH_2$), 1.95-1.88 (m, 1H, $COCH_2$) ppm. Monomer (minor component): δ = 7.01 (dd, J = 7.9, 1.5 Hz, 1H, H_{arom}), 6.36 (dd, J = 7.9, 1.5 Hz, 1H, H_{arom}), 2.91 (t, J = 6.8 Hz, 2H, CH_2) ppm. Signals not listed are overlapping and cannot be assigned.

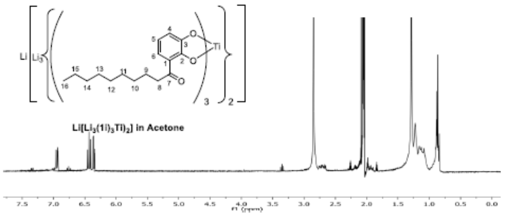

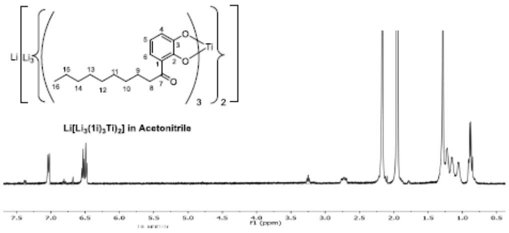

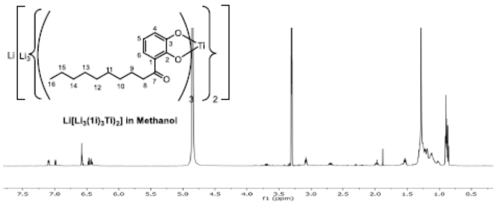

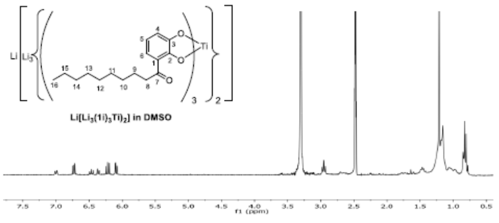

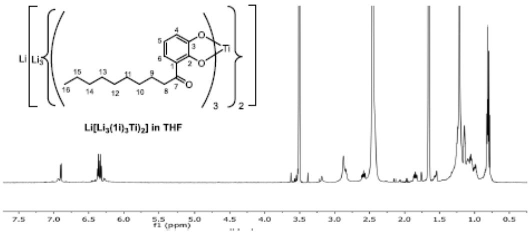

$Li[Li_3(1j)_3Ti)_2]$: **^{1}H-NMR** (600 MHz, Acetone-d_6): Dimer (major component): δ = 7.02 (dd, J = 8.2, 1.6 Hz, 1H, H_{arom}), 6.46 (t, J = 8.2 Hz, 1H, H_{arom}), 6.38 (dd, J = 8.2, 1.6 Hz, 1H, H_{arom}), 2.77-2.66 (m, 1H, $COCH_2$), 1.98-1.91 (m, 1H, $COCH_2$), 1.28-1.05 (m, 16H), 0.87 (t, J = 6.8 Hz, 3H, CH_3) ppm. Monomer (minor component): δ = 7.49 (dd, J = 8.1, 1.6 Hz, 1H, H_{arom}), 7.09 (dd, J = 8.1, 1.6 Hz, 1H, H_{arom}), 6.76 (t, J = 8.1 Hz, 1H, H_{arom}), 3.08 (t, J = 6.5 Hz, 2H, CH_2), 1.33-1.29 (m, 16H), 0.95 (t, J = 6.8 Hz, 3H, CH_3) ppm. **^{1}H-NMR** (600 MHz, ACN-d_3): Dimer (major component): δ = 7.07 (dd, J = 7.8, 1.6 Hz, 1H, H_{arom}), 6.54 (t, J = 7.8 Hz, 1H, H_{arom}), 6.49 (dd, J = 7.8, 1.6 Hz, 1H, H_{arom}), 2.77-2.70 (m,1H, $COCH_2$), 1.09-1.04 (m, 2H, CH_2) ppm. Monomer (minor component): δ = 7.49 (dd, J = 7.8, 1.6 Hz, 1H, H_{arom}), 6.89 (t, J = 7.8 Hz, 1H, H_{arom}), 6.77 (dd, J = 7.8, 1.6 Hz, 1H, H_{arom}), 3.04 (t, J = 6.8 Hz, 2H, CH_2) ppm. Signals not listed are overlapping and cannot be assigned. **^{1}H-NMR** (600 MHz, DMSO-d_6): Dimer (minor component): δ = 7.00 (dd, J = 8.1, 1.6 Hz, 1H, H_{arom}), 6.46 (t, J = 8.1 Hz, 1H, H_{arom}), 6.36 (dd, J = 8.1, 1.6 Hz, 1H, H_{arom}), 2.71-2.58 (m,1H, $COCH_2$), 1.80-1.70 (m,1H, $COCH_2$) ppm. Monomer (major component): δ = 6.73 (dd, J = 8.1, 1.6 Hz, 1H, H_{arom}), 6.22 (t, J = 8.1 Hz, 1H, H_{arom}), 6.09 (dd, J = 8.1, 1.6 Hz, 1H, H_{arom}), 2.96 (t, J = 7.3 Hz, 2H, CH_2) ppm. Signals not listed are overlapping and cannot be assigned. **^{1}H-NMR** (600 MHz, THF-d_8): Dimer (major component): δ = 6.82 (dd, J = 7.9, 1.5 Hz, 1H, H_{arom}), 6.30-6.25 (m, 2H, H_{arom}), 2.55-2.49 (m,1H, $COCH_2$), 1.80-1.75 (m, 1H, $COCH_2$) ppm. Monomer (minor component): δ = 6.87 (dd, J = 7.9, 1.5 Hz, 1H, H_{arom}), 6.21 (dd, J = 7.9, 1.5 Hz, 1H, H_{arom}), 2.77 (t, J = 6.5 Hz, 2H, CH_2) ppm. Signals not listed are overlapping and cannot be assigned.

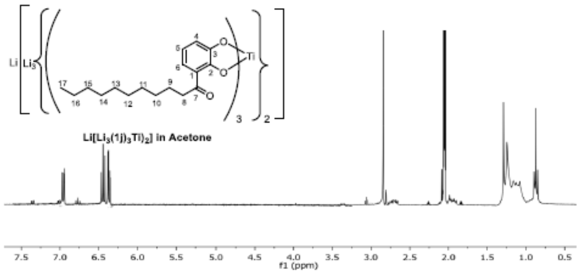

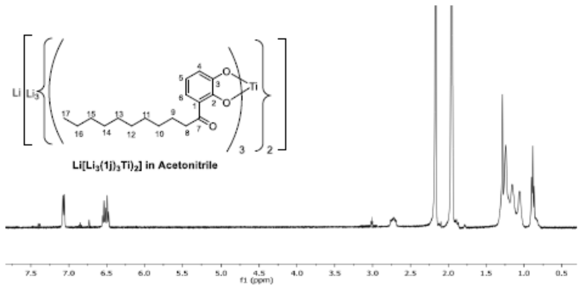

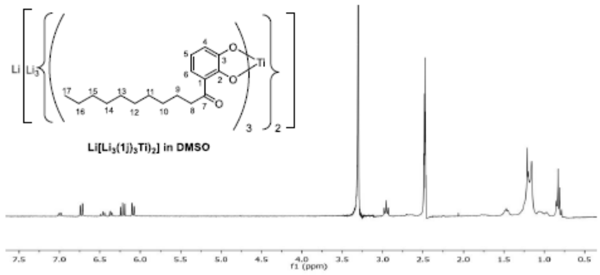

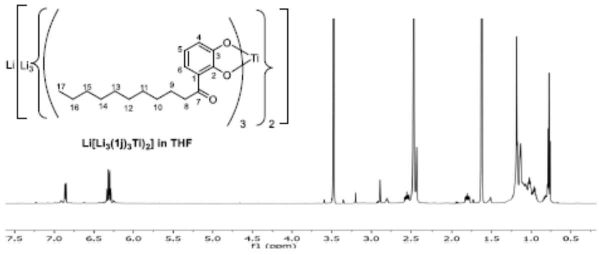

Li[Li$_3$(1k)$_3$Ti)$_2$]: **^{1}H-NMR** (600 MHz, Acetone-d$_6$): Dimer (major component): *δ* = 7.02 (dd, *J* = 8.1, 1.6 Hz, 1H, H_{arom}), 6.45 (t, *J* = 8.1 Hz, 1H, H_{arom}), 6.39 (dd, *J* = 8.1, 1.6 Hz, 1H, H_{arom}), 2.76-2.63 (m,1H, $COCH_2$), 2.00-1.89 (m, 1H, $COCH_2$), 1.30-1.06 (m, 18H), 0.88 (t, *J* = 6.9 Hz, 3H, CH_3) ppm. Monomer (minor component): *δ* = 7.53 (dd, *J* = 8.1, 1.6 Hz, 1H, H_{arom}), 7.19 (dd, *J* = 8.1, 1.6 Hz, 1H, H_{arom}), 6.86 (t, *J* = 8.1 Hz, 1H, H_{arom}), 3.08 (t, *J* = 6.8 Hz, 2H, CH_2), 1.36-1.32 (m, 18H), 0.94 (t, *J* = 6.9 Hz, 3H, CH_3) ppm. **^{1}H-NMR** (600 MHz, ACN-d$_3$): Dimer (major component): *δ* = 7.06 (dd, *J* = 7.8, 1.6 Hz, 1H, H_{arom}), 6.53 (t, *J* = 7.8 Hz, 1H, H_{arom}), 6.49 (dd, *J* = 7.8, 1.6 Hz, 1H, H_{arom}), 2.76-2.68 (m,1H, $COCH_2$), 1.18-1.10 (m, 2H, CH_2), 1.08-1.02 (m, 2H, CH_2) ppm. Monomer (minor component): *δ* = 7.49 (dd, *J* = 7.8, 1.6 Hz, 1H, H_{arom}), 6.89 (t, *J* = 7.8 Hz, 1H, H_{arom}), 6.77 (dd, *J* = 7.8, 1.6 Hz, 1H, H_{arom}), 3.04 (t, *J* = 6.8 Hz, 2H, CH_2), 1.72-1.66 (m, 2H, CH_2), 1.59-1.53 (m, 2H, CH_2) ppm. Signals not listed are overlapping and cannot be assigned. **^{1}H-NMR** (600 MHz, MeOH-d$_4$): Dimer (major component): *δ* = 7.09 (dd, *J* = 7.8, 1.5 Hz, 1H, H_{arom}), 6.59-6.56 (m, 2H, H_{arom}), 2.72-2.66 (m,1H, $COCH_2$), 2.00-1.94 (m, 1H, $COCH_2$) ppm. Monomer (minor component): *δ* = 7.00 (dd, *J* = 7.8, 1.5 Hz, 1H, H_{arom}), 6.48-6.42 (m, 2H, H_{arom}), 2.96 (t, *J* = 7.2 Hz, 2H, CH_2) ppm. Signals not listed are overlapping and cannot be assigned. **^{1}H-NMR** (600 MHz, DMSO-d$_6$): Dimer (minor component): *δ* = 6.84 (dd, *J* = 8.2, 1.5 Hz, 1H, H_{arom}), 6.45 (t, *J* = 8.2 Hz, 1H, H_{arom}), 6.36 (dd, *J* = 8.2, 1.5 Hz, 1H, H_{arom}), 2.73-2.69 (m,1H, $COCH_2$) ppm. Monomer (major component): *δ* = 6.73 (dd, *J* = 8.2, 1.5 Hz, 1H, H_{arom}), 6.35 (t, *J* = 8.2 Hz, 1H, H_{arom}), 6.09 (dd, *J* = 8.2, 1.5 Hz, 1H, H_{arom}), 2.95 (t, *J* = 7.3 Hz, 2H, CH_2) ppm. Signals not listed are overlapping and cannot be assigned. **^{1}H-NMR** (600 MHz, THF-d$_8$): Dimer (major component): *δ* = 6.96 (dd, *J* = 7.8, 1.5 Hz, 1H, H_{arom}), 6.44-6.38 (m, 2H, H_{arom}), 2.69-2.63 (m,1H, $COCH_2$), 1.99-1.89 (m, 1H, $COCH_2$) ppm. Monomer (minor component): *δ* = 7.00 (dd, *J* = 7.8, 1.5 Hz, 1H, H_{arom}), 6.35 (dd, *J* = 7.8, 1.5 Hz, 1H, H_{arom}), 2.91 (t, *J* = 6.8 Hz, 2H, CH_2) ppm. Signals not listed are overlapping and cannot be assigned.

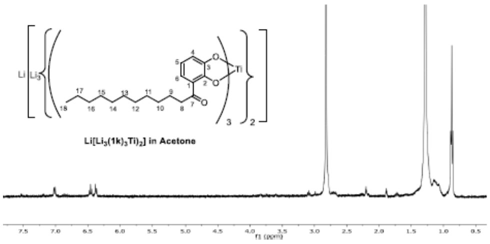

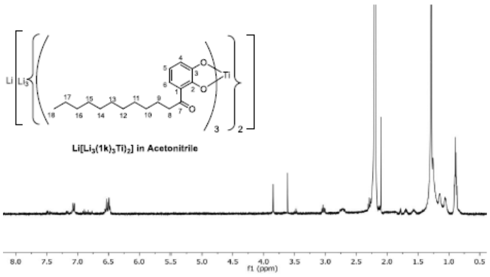

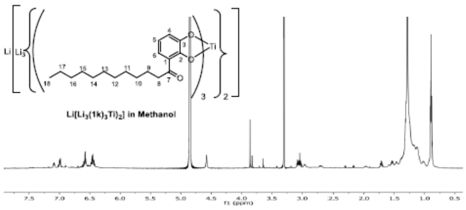

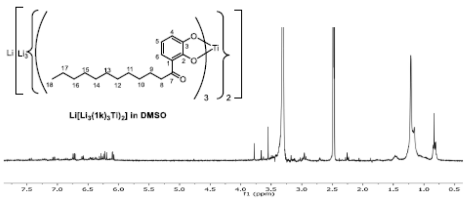

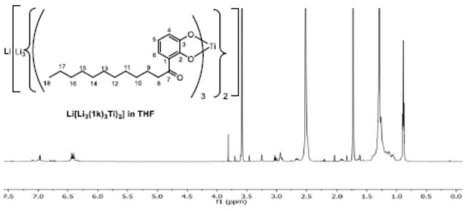

Li[Li$_3$(1l)$_3$Ti)$_2$]: **^{1}H-NMR** (600 MHz, Acetone-d$_6$): Dimer (major component): δ = 7.02 (dd, J = 8.0, 1.5 Hz, 1H, H$_{arom}$), 6.47 (t, J = 8.0 Hz, 1H, H$_{arom}$), 6.38 (dd, J = 8.0, 1.5 Hz, 1H, H$_{arom}$), 2.72-2.68 (m, 1H, COCH$_2$), 1.98-1.87 (m, 1H, COCH$_2$), 1.29-1.05 (m, 20H), 0.89 (t, J = 7.2 Hz, 3H, CH$_3$) ppm. Monomer (minor component): δ = 7.46 (dd, J = 8.0, 1.5 Hz, 1H, H$_{arom}$), 7.09 (dd, J = 8.0, 1.5 Hz, 1H, H$_{arom}$), 6.76 (t, J = 8.0 Hz, 1H, H$_{arom}$), 3.07 (t, J = 6.5 Hz, 2H, CH$_2$), 1.34-1.30 (m, 20H), 0.94 (t, J = 7.2 Hz, 3H, CH$_3$) ppm. **^{1}H-NMR** (600 MHz, ACN-d$_3$): Dimer (major component): δ = 7.07 (dd, J = 7.9, 1.6 Hz, 1H, H$_{arom}$), 6.54 (t, J = 7.9 Hz, 1H, H$_{arom}$), 6.49 (dd, J = 7.9, 1.6 Hz, 1H, H$_{arom}$), 2.76-2.71 (m,1H, COCH$_2$), 1.19-1.13 (m, 4H), 1.09-1.04 (m, 4H, 2× CH$_2$) ppm. Monomer (minor component): δ = 7.44 (dd, J = 7.9, 1.6 Hz, 1H, H$_{arom}$), 6.84 (t, J = 7.9 Hz, 1H, H$_{arom}$), 6.69 (dd, J = 7.9, 1.6 Hz, 1H, H$_{arom}$), 3.04 (t, J = 6.8 Hz, 2H, COCH$_2$), 1.71-1.70 (m, 2H, CH$_2$) ppm. Signals not listed are overlapping and cannot be assigned. **^{1}H-NMR** (600 MHz, DMSO-d$_6$): Dimer (minor component): δ = 6.99 (dd, J = 8.1, 1.6 Hz, 1H, H$_{arom}$), 6.45 (t, J = 8.1 Hz, 1H, H$_{arom}$), 6.36 (dd, J = 8.1, 1.6 Hz, 1H, H$_{arom}$), 2.67-2.60 (m,1H, COCH$_2$), 1.79-1.66 (m, 1H, COCH$_2$) ppm. Monomer (major component): δ = 6.73 (dd, J = 8.1, 1.6 Hz, 1H, H$_{arom}$), 6.22 (t, J = 8.1 Hz, 1H, H$_{arom}$), 6.08 (dd, J = 8.1, 1.6 Hz, 1H, H$_{arom}$), 2.96 (t, J = 7.2 Hz, 2H, COCH$_2$) ppm. Signals not listed are overlapping and cannot be assigned. **^{1}H-NMR** (600 MHz, THF-d$_8$): Dimer (major component): δ = 6.97 (dd, J = 7.8, 1.6 Hz, 1H, H$_{arom}$), 6.44-6.39 (m, 2H, H$_{arom}$), 2.69-2.63 (m,1H, COCH$_2$), 1.94-1.89 (m, 1H, COCH$_2$) ppm. Monomer (minor component): δ = 7.02 (dd, J = 7.8, 1.6 Hz, 1H, H$_{arom}$), 6.36 (dd, J = 7.8, 1.6 Hz, 1H, H$_{arom}$), 2.91 (t, J = 6.5 Hz, 2H, COCH$_2$) ppm. Signals not listed are overlapping and cannot be assigned.

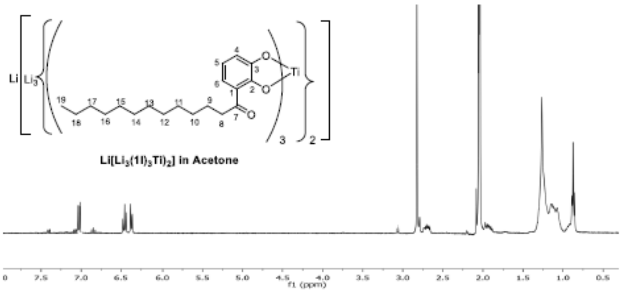

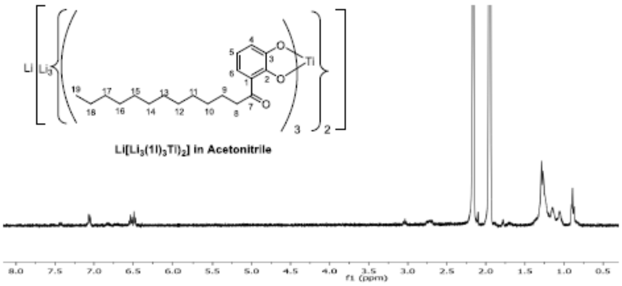

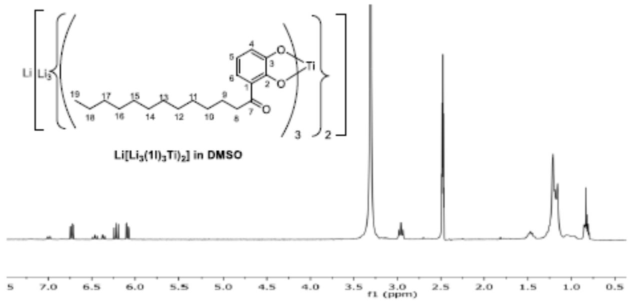

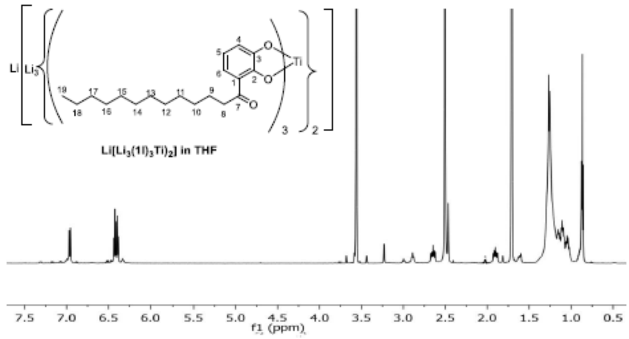

Li[Li$_3$(1m)$_3$Ti)$_2$]: **^{1}H-NMR** (400 MHz, Acetone-d$_6$): Dimer (major component): δ = 7.03 (dd, J = 7.9, 1.5 Hz, 1H, H_{arom}), 6.48 (t, J = 7.9 Hz, 1H, H_{arom}), 6.38 (dd, J = 7.9, 1.5 Hz, 1H, H_{arom}), 2.60 (dd, J = 15.8, 8.8 Hz, 1H, $COCH_2$), 1.68 (dd, J = 15.8, 4.7 Hz, 1H, $COCH_2$), 1.61-1.53 (m, 1H, CH), 0.65 (d, J = 6.7 Hz, 3H, CH_3), 0.58 (d, J = 6.7 Hz, 3H, CH_3) ppm. Monomer (minor component): δ = 7.43 (dd, J = 7.9, 1.5 Hz, 1H, H_{arom}), 7.14 (dd, J = 7.9, 1.5 Hz, 1H, H_{arom}), 6.86 (t, J = 7.9 Hz, 1H, H_{arom}), 3.73 (d, J = 6.5 Hz, 2H, $COCH_2$), 1.89-1.86 (m, 1H, CH), 1.27 (d, J = 6.7 Hz, 6H, 2×CH_3) ppm. **^{1}H-NMR** (600 MHz, ACN-d$_3$): Dimer (major component): δ = 7.09 (dd, J = 7.8, 1.5 Hz, 1H, H_{arom}), 6.57 (t, J = 7.8 Hz, 1H, H_{arom}), 6.51 (dd, J = 7.8, 1.5 Hz, 1H, H_{arom}), 2.64 (dd, J = 15.8, 5.0 Hz, 1H, $COCH_2$), 1.62 (dd, J = 15.8, 5.0 Hz, 1H, $COCH_2$), 1.53-1.46 (m, 1H, CH), 0.68 (d, J = 6.5 Hz, 3H, CH_3), 0.62 (d, J = 6.5 Hz, 3H, CH_3) ppm. Monomer (minor component): δ = 7.44 (dd, J = 7.8, 1.5 Hz, 1H, H_{arom}), 6.85 (t, J = 7.8 Hz, 1H, H_{arom}), 6.70 (dd, J = 7.8, 1.5 Hz, 1H, H_{arom}), 3.32-3.29 (m, 2H, $COCH_2$), 1.86-1.84 (m, 1H, CH), 1.01 (d, J = 6.5 Hz, 6H, 2×CH_3) ppm. **^{1}H-NMR** (400 MHz, MeOH-d$_4$): Dimer (minor component): δ = 7.10 (dd, J = 7.4, 2.3 Hz, 1H, H_{arom}), 6.60-6.58 (m, 2H, H_{arom}), 2.56 (dd, J = 16.1, 8.8 Hz, 1H, $COCH_2$), 1.70 (dd, J = 16.1, 8.8 Hz, 1H, $COCH_2$), 1.52-1.44 (m, 1H, CH), 0.65 (d, J = 6.8 Hz, 3H, CH_3), 0.55 (d, J = 6.8 Hz, 3H, CH_3) ppm. Monomer (major component): δ = 6.96 (dd, J = 7.4, 2.3 Hz, 1H, H_{arom}), 6.47-6.40 (m, 2H, H_{arom}), 2.90-2.88 (d, 2H, $COCH_2$), 2.12-2.05 (m, 1H, CH), 0.75 (d, J = 6.8 Hz, 6H, 2×CH_3) ppm. **^{1}H-NMR** (600 MHz, DMSO-d$_6$): Dimer (minor component): δ = 7.02 (dd, J = 7.9, 1.5 Hz, 1H, H_{arom}), 6.50 (t, J = 7.9 Hz, 1H, H_{arom}), 6.39 (dd, J = 7.9, 1.5 Hz, 1H, H_{arom}), 2.82 (dd, J = 12.8, 8.9 Hz, 1H, $COCH_2$), 2.61 (dd, J = 12.8, 8.7 Hz, 1H, $COCH_2$), 1.44-1.38 (m, 1H, CH), 0.59 (d, J = 6.7 Hz, 3H, CH_3), 0.53 (d, J = 6.7 Hz, 3H, CH_3) ppm. Monomer (major component): δ = 6.73 (dd, J = 7.9, 1.5 Hz, 1H, H_{arom}), 6.21 (t, J = 7.9 Hz, 1H, H_{arom}), 6.08 (dd, J = 7.9, 1.5 Hz, 1H, H_{arom}), 3.77 (d, J = 6.5 Hz, 2H, $COCH_2$), 1.49-1.45 (m, 1H, CH), 0.76 (d, J = 6.7 Hz, 6H, 2×CH_3) ppm. **^{1}H-NMR** (600 MHz, THF-d$_8$): Dimer (major component): δ = 7.00 (dd, J = 8.2, 1.6 Hz, 1H, H_{arom}), 6.46 (t, J = 8.2 Hz, 1H, H_{arom}), 6.42 (dd, J = 8.2, 1.6 Hz, 1H, H_{arom}), 2.58 (dd, J = 15.4, 8.5 Hz, 1H, $COCH_2$),1.66 (dd, J = 15.4, 8.5 Hz, 1H, $COCH_2$), 1.62-1.57 (m, 1H , CH), 0.66 (d, J = 6.4 Hz, 3H, CH_3), 0.59 (d, J = 6.4 Hz, 3H, CH_3) ppm. Monomer (minor component): δ = 7.06 (dd, J = 8.2, 1.6 Hz, 1H, H_{arom}), 6.44 (t, J = 8.2 Hz, 1H, H_{arom}), 6.34 (dd, J = 8.2, 1.6 Hz, 1H, H_{arom}), 2.76 (d, J = 6.6 Hz, 2H, $COCH_2$), 2.19-2.23 (m, 1H, CH), 0.91 (d, J = 6.4 Hz, 6H, 2×CH_3) ppm. **MS** (negative ESI-MS, MeOH): *m/z* (%) = 1269.4163 (100, [M_D-Li^+], $C_{66}H_{72}O_{18}Li_3Ti_2^-$, calcd. 1269.4158). **IR (KBr)**: $\tilde{\nu}$ (cm^{-1}) = 3428, 3064, 2955, 2869, 2433, 2289, 2179, 2114, 2080, 2015, 1965, 1934, 1897, 1725, 1647, 1592, 1547, 1426, 1360, 1301, 1254, 1211, 1170, 1100, 1054, 959, 907, 846, 758, 732, 670. **Elemental analysis:** $C_{66}H_{72}O_{18}Li_4Ti_2 \cdot 8\ H_2O$: calcd. C = 55.79 %, H = 6.24 %; found C = 55.81 %, H = 6.05 %.

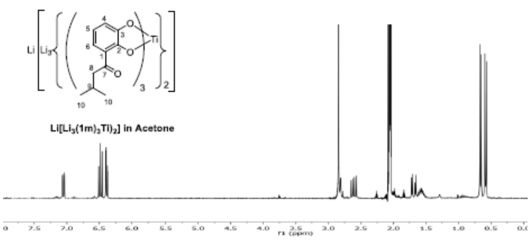

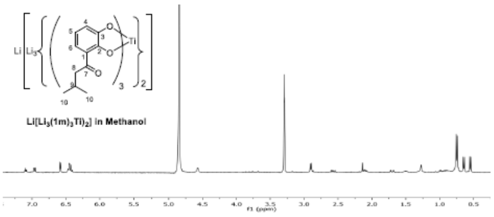

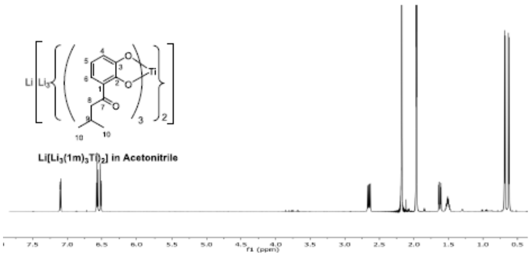

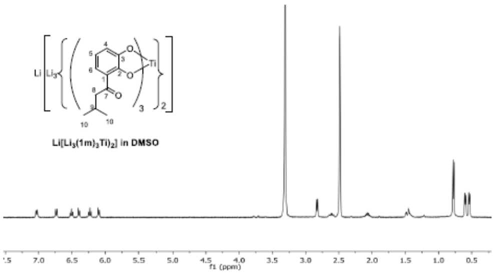

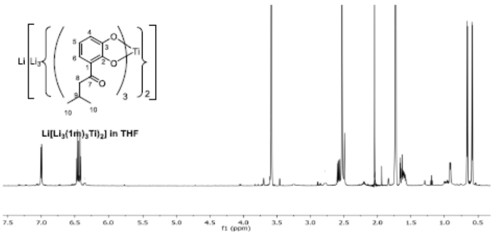

$Li[Li_3(1n)_3Ti)_2]$: **^{1}H-NMR** (400 MHz, Acetone-d$_6$): Dimer (major component): δ = 7.02 (dd, J = 8.0, 1.5 Hz, 1H, H_{arom}), 6.47 (t, J = 8.0 Hz, 1H, H_{arom}), 6.41 (dd, J = 8.0, 1.5 Hz, 1H, H_{arom}), 4.04 (dd, J = 13.8, 7.5 Hz, 1H, $COCH_2$), 3.67 (dd, J = 13.8, 7.5 Hz, 1H, $COCH_2$) ppm. Monomer: Signals are low in intensity and overlap with those of the dimers. **^{1}H-NMR** (600 MHz, ACN-d$_3$): Dimer (major component): δ = 7.05 (dd, J = 7.9, 1.5 Hz, 1H, H_{arom}), 6.59-6.53 (m, 2H, H_{arom}), 4.04 (dd, J = 15.7, 4.6 Hz, 1H, $COCH_2$), 3.62 (dd, J = 15.7, 4.6 Hz, 1H, $COCH_2$) ppm. Monomer (minor component): δ = 6.89 (t, J = 7.9 Hz, 1H, H_{arom}), 6.70 (dd, J = 7.9, 1.5 Hz, 1H, H_{arom}), 4.10 (dd, J = 7.7, 12.6 Hz, 2H, $COCH_2$) ppm. Signals not listed are overlapping and cannot be assigned. **^{1}H-NMR** (600 MHz, MeOH-d$_4$): Dimer (minor component): δ = 7.10 (dd, J = 7.8, 1.5 Hz, 1H, H_{arom}), 6.61-6.54 (m, 2H, H_{arom}), 3.19 (dd, J = 12.5, 6.4 Hz, 1H, $COCH_2$), 2.76 (dd, J = 12.5, 6.4 Hz, 1H, $COCH_2$) ppm. Monomer (major component): δ = 6.97 (dd, J = 7.8, 1.5 Hz, 1H, H_{arom}), 6.47-6.42 (m, 2H, H_{arom}), 3.92 (d, J = 7.4 Hz, 2H, $COCH_2$) ppm. Signals not listed are overlapping and cannot be assigned. **^{1}H-NMR** (600 MHz, DMSO-d$_6$): Dimer (minor component): δ = 7.01 (dd, J = 7.8, 1.8 Hz, 1H, H_{arom}), 6.49 (t, J = 7.8 Hz, 1H, H_{arom}), 6.40 (dd, J = 7.8, 1.8 Hz, 1H, H_{arom}), 2.98-2.95 (dd, J = 12.5, 6.4 Hz, 1H, $COCH_2$) ppm. Monomer (major component): 6.74 (dd, J = 7.8, 1.8 Hz, 1H, H_{arom}), 6.24 (t, J = 7.8 Hz, 1H, H_{arom}), 6.11 (dd, J = 7.8, 1.8 Hz, 1H, H_{arom}), 3.10 (d, J = 7.0 Hz, 2H, $COCH_2$) ppm. Signals not listed are overlapping and cannot be assigned. **^{1}H-NMR** (600 MHz, THF-d$_8$): Dimer (major component): δ = 6.85 (dd, J = 7.5, 1.6 Hz, 1H, H_{arom}), 6.35-6.27 (m, 2H, H_{arom}), 3.81 (dd, J = 7.2, 12.6 Hz, 1H, $COCH_2$) ppm. Monomer (minor component): δ = 7.01 (dd, J = 7.5, 1.6 Hz, 1H, H_{arom}), 6.24-6.20 (m, 2H, H_{arom}), 4.09 (d, J = 7.0 Hz, 2H, $COCH_2$) ppm. Signals not listed are overlapping and cannot be assigned. **MS** (negative and positive ESI-MS, MeOH): *m/z* (%) = 1341.4044 (100, $[M_D\text{-}Li^+]$, $C_{72}H_{72}O_{18}Li_3Ti_2^-$, calcd. 1341.4158), 667.1920 (98, $[M_M\text{-}Li^+]$, $C_{36}H_{36}O_9LiTi^-$, calcd. 667.1999); 1355.4500 (20, $[M_D{+}Li^+]$, $C_{72}H_{72}O_{18}Li_5Ti_2^+$, calcd. 1355.4478). **IR (KBr)**:$\tilde{\nu}$ (cm^{-1}) = 3841, 3392, 2932, 2862, 2656, 2460, 2323, 2234, 2206, 2162, 2114, 2010, 1968, 1934, 1891, 1742, 1647, 1591, 1547, 1431, 1366, 1310, 1255, 1215, 1101, 1058, 909, 846, 793, 734, 671.

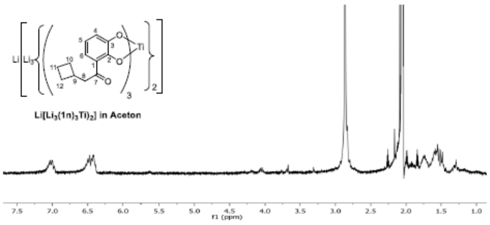

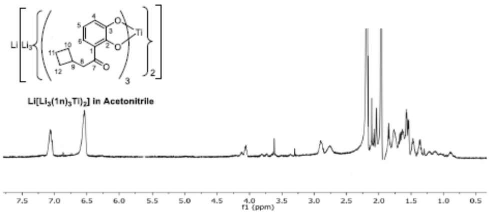

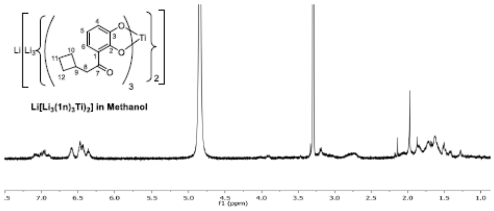

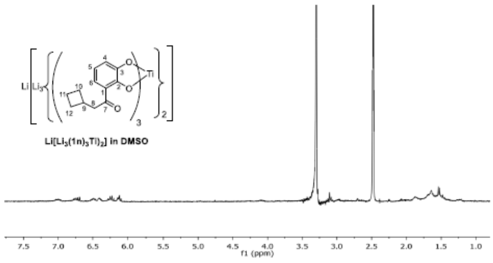

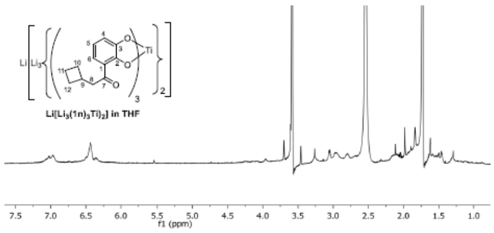

Li[Li_3(1o)$_3$Ti)$_2$]: **^{1}H-NMR** (400 MHz, Acetone-d_6): Dimer (major component): δ = 7.01 (dd, *J* = 7.8, 1.5 Hz, 1H, H_{arom}), 6.46 (t, *J* = 7.8 Hz, 1H, H_{arom}), 6.38 (dd, *J* = 7.8, 1.5 Hz, 1H, H_{arom}), 2.64 (dd, *J* = 14.9, 7.6 Hz, 1H, $COCH_2$),1.53-1.42 (m, 5H, H_{cycl}) ppm. Monomer (minor component): δ = 7.47 (dd, *J* = 7.8, 1.5 Hz, 1H, H_{arom}), 7.11 (dd, *J* = 7.8, 1.5 Hz, 1H, H_{arom}), 6.83 (t, *J* = 7.8 Hz, 1H, H_{arom}), 3.76-3.72 (m, 2H, $COCH_2$) ppm. Signals not listed are overlapping and cannot be assigned. **^{1}H-NMR** (600 MHz, ACN-d_3): Dimer (major component): δ = 7.07 (dd, *J* = 8.0, 1.5 Hz, 1H, H_{arom}), 6.55 (t, *J* = 8.0 Hz, 1H, H_{arom}), 6.52 (dd, *J* = 8.0, 1.5 Hz, 1H, H_{arom}), 2.67 (dd, *J* = 14.9, 7.6 Hz, 1H, $COCH_2$) ppm. Monomer (minor component): δ = 7.46 (dd, *J* = 8.0, 1.5 Hz, 1H, H_{arom}), 7.12 (dd, *J* = 8.0, 1.5 Hz, 1H, H_{arom}), 6.84 (t, *J* = 8.0 Hz, 1H, H_{arom}), 3.30 (d, *J* = 7.5 Hz, 2H, $COCH_2$) ppm. Signals not listed are overlapping and cannot be assigned. **^{1}H-NMR** (400 MHz, MeOH-d_4): Dimer (minor component): δ = 7.06 (dd, *J* = 8.2, 1.5 Hz, 1H, H_{arom}), 6.61-6.56 (m, 2H, H_{arom}), 2.61 (dd, *J* = 11.5, 6.8 Hz, 1H, $COCH_2$) ppm. Monomer (major component): δ = 6.98 (dd, *J* = 7.9, 1.5 Hz, 1H, H_{arom}), 6.47-6.41 (m, 2H, H_{arom}), 2.94 (d, *J* = 6.8 Hz, 2H, $COCH_2$) ppm. Signals not listed are overlapping and cannot be assigned. **^{1}H-NMR** (600 MHz, DMSO-d_6): Dimer (minor component): δ = 7.01 (dd, *J* = 8.2, 1.5 Hz, 1H, H_{arom}), 6.50 (t, *J* = 8.2 Hz, 1H, H_{arom}), 6.39 (dd, *J* = 8.2, 1.5 Hz, 1H, H_{arom}), 2.64 (dd, *J* = 12.7, 6.6 Hz, 1H, $COCH_2$), 1.71 (dd, *J* = 12.7, 6.6 Hz, 1H, $COCH_2$), 1.28-1.22 (m, 5H, H_{cycl}), 0.84-0.78 (m, 5H, H_{cycl}), 0.70-0.65 (m, 1H, H_{cycl}) ppm. Monomer (major component): δ = 6.73 (dd, *J* = 8.2, 1.5 Hz, 1H, H_{arom}), 6.21 (t, *J* = 8.2 Hz, 1H, H_{arom}), 6.09 (dd, *J* = 8.2, 1.5 Hz, 1H, H_{arom}), 2.84 (d, *J* = 6.6 Hz, 2H, $COCH_2$), 1.60-1.57 (m, 1H, H_{cycl}), 1.43-1.40 (m, 5H, H_{cycl}), 0.97-0.91 (m, 5H, H_{cycl}) ppm. **^{1}H-NMR** (600 MHz, THF-d_8): Dimer (major component): δ = 6.84 (dd, *J* = 7.8, 1.6 Hz, 1H, H_{arom}), 6.31-6.27 (m, 2H, H_{arom}), 2.48 (dd, *J* = 14.6, 7.6 Hz, 1H, $COCH_2$) ppm. Monomer (minor component): δ = 6.86 (dd, *J* = 7.8, 1.6 Hz, 1H, H_{arom}), 6.22-6.20 (m, 2H, H_{arom}), 2.63 (d, *J* = 6.8 Hz, 2H, $COCH_2$) ppm. Signals not listed are overlapping and cannot be assigned. **MS** (negative and positive ESI-MS, MeOH): *m/z* (%) = 1509.5965 (80, [M_D-Li^+], $C_{84}H_{96}O_{18}Li_3Ti_2^-$, calcd. 1509.6036), 751.2896 (100, [M_M-Li^+], $C_{42}H_{48}O_9LiTi^-$, calcd. 751.2938); 1523.6375 (50, [M_D+Li^+], $C_{84}H_{96}O_{18}Li_5Ti_2^+$, calcd. 1523.6356), 765.3265 (100, [M_M+Li^+], $C_{42}H_{48}O_9Li_3Ti^+$, calcd. 765.3258). **IR (KBr)**: $\tilde{\nu}$ (cm^{-1}) = 3893, 3374, 2920, 2848, 2656, 2323, 2234, 2208, 2159, 2103, 2047, 2028, 1992, 1968, 1934, 1886, 1743, 1647, 1591, 1548, 1426, 1351, 1297, 1254, 1213, 1106, 1054, 903, 844, 805, 779, 732, 673. **Elemental analysis** $C_{84}H_{96}O_{18}Li_4Ti_2 \cdot 10\ H_2O$: calcd. C = 59.44 %, H = 6.89 %; found C = 59.72 %, H = 6.78 %.

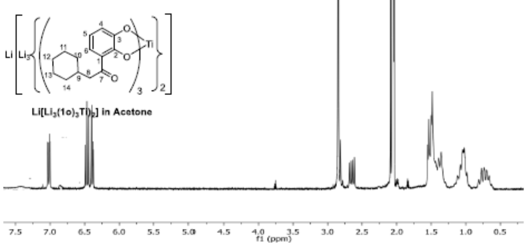

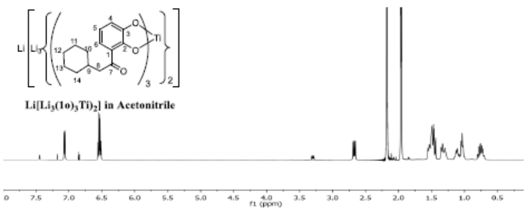

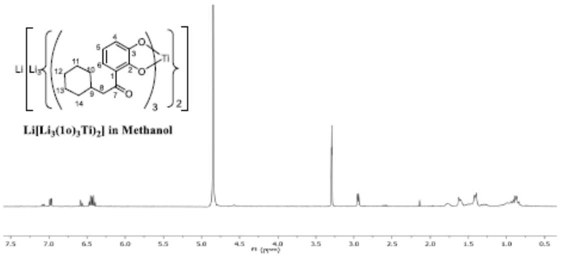

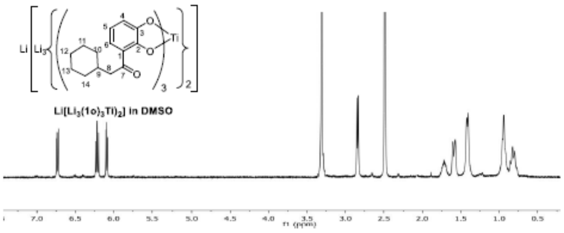

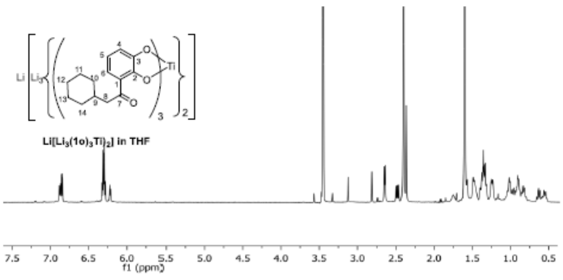

Li[Li_3(1p)$_3$Ti)$_2$]: **^{1}H-NMR** (400 MHz, Acetone-d_6): Dimer (major component): δ = 7.00 (dd, J = 7.8, 1.5 Hz, 1H, H_{arom}), 6.45 (t, J = 7.8 Hz, 1H, H_{arom}), 6.36 (dd, J = 7.8, 1.5 Hz, 1H, H_{arom}), 3.14-3.05 (m, 1H, COCH), 0.80 (d, J = 6.5 Hz, 3H, CH_3), 0.40 (d, J = 6.5 Hz, 3H, CH_3) ppm. Monomer (minor component): δ = 7.47 (dd, J = 7.8, 1.5 Hz, 1H, H_{arom}), 7.10 (dd, J = 7.8, 1.5 Hz, 1H, H_{arom}), 6.82 (t, J = 7.8 Hz, 1H, H_{arom}), 3.75-3.66 (m, 1H, COCH), 1.05 (d, J = 6.5 Hz, 6H, 2×CH_3) ppm. **^{1}H-NMR** (600 MHz, ACN-d_3): Dimer (major component): δ = 7.08 (dd, J = 7.9, 1.6 Hz, 1H, H_{arom}), 6.55 (t, J = 7.9 Hz, 1H, H_{arom}), 6.49 (dd, J = 7.9, 1.6 Hz, 1H, H_{arom}), 3.17-3.13 (m,1H, COCH), 0.82 (d, J = 6.9 Hz, 3H, CH_3), 0.40 (d, J = 6.9 Hz, 3H, CH_3) ppm. Monomer (minor component): δ = 7.47 (dd, J = 7.9, 1.6 Hz, 1H, H_{arom}), 6.86 (t, J = 7.9 Hz, 1H, H_{arom}), 6.71 (dd, J = 7.9, 1.6 Hz, 1H, H_{arom}), 3.73-3.69 (m, 1H, COCH), 1.21 (d, J = 6.9 Hz, 6H, 2×CH_3) ppm. **^{1}H-NMR** (400 MHz, CD_3OD-d_4): Dimer (minor component): δ = 7.37 (dd, J = 7.4, 2.3 Hz, 1H, H_{arom}), 6.78 (t, J = 7.4 Hz, 1H, H_{arom}), 6.54 (dd, J = 7.4, 2.3 Hz, 1H, H_{arom}), 3.71-3.68 (m, 1H, COCH), 0.77 (d, J = 6.5 Hz, 3H, CH_3), 0.38 (d, J = 6.5 Hz, 3H, CH_3) ppm. Monomer (major component): δ = 6.97 (dd, J = 7.4, 2.3 Hz, 1H, H_{arom}), 6.49-6.42 (m, 2H, H_{arom}), 4.05-3.99 (m,1H, COCH), 1.03 (d, J = 6.5 Hz, 6H, 2×CH_3) ppm. **^{1}H-NMR** (600 MHz, DMSO-d_6): Dimer (minor component): δ = 7.00 (dd, J = 8.1, 1.6 Hz, 1H, H_{arom}), 6.49 (t, J = 8.1 Hz, 1H, H_{arom}), 6.36 (dd, J = 8.1, 1.6 Hz, 1H, H_{arom}), 3.12-3.08 (m, 1H, COCH), 0.70 (d, J = 6.8 Hz, 3H, CH_3), 0.29 (d, J = 6.8 Hz, 3H, CH_3) ppm. Monomer (major component): δ = 6.75 (dd, J = 8.1, 1.6 Hz, 1H, H_{arom}), 6.24 (t, J = 8.1 Hz, 1H, H_{arom}), 6.11 (dd, J = 8.1, 1.6 Hz, 1H, H_{arom}), 4.03-3.94 (m, 1H, COCH), 0.96 (d, J = 6.8 Hz, 6H, 2×CH_3) ppm. **^{1}H-NMR** (600 MHz, THF-d_8): Dimer (major component): δ = 7.35 (dd, J = 7.8, 1.6 Hz, 1H, H_{arom}), 6.99 (dd, J = 7.8, 1.6 Hz, 1H, H_{arom}), 6.74 (t, J = 7.8 Hz, 1H, H_{arom}), 3.09-3.05 (m, 1H, COCH), 0.80 (d, J = 6.8 Hz, 3H, CH_3), 0.40 (d, J = 6.8 Hz, 3H, CH_3) ppm. Monomer (minor component): δ = 7.01 (dd, J = 7.8, 1.6 Hz, 1H, H_{arom}), 6.45 (t, J = 7.8 Hz, 1H, H_{arom}), 6.36 (dd, J = 7.8, 1.6 Hz, 1H, H_{arom}), 3.64-3.60 (m, 1H, COCH), 1.10 (d, J = 6.8 Hz, 6H, 2×CH_3) ppm.

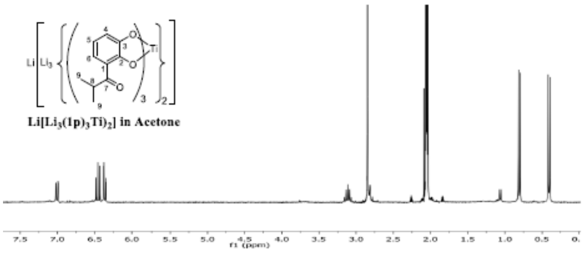

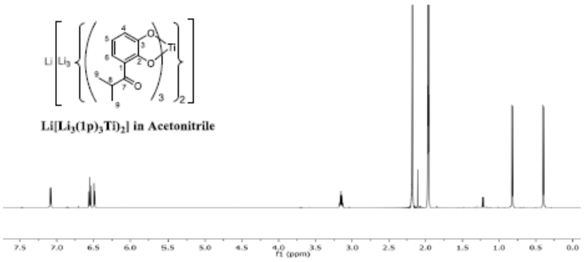

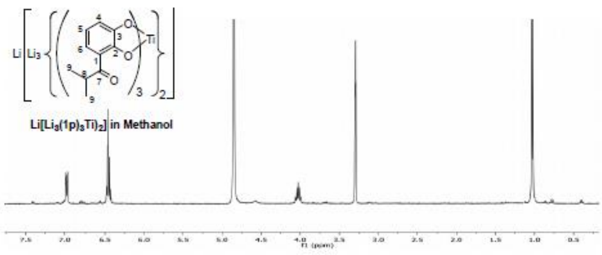

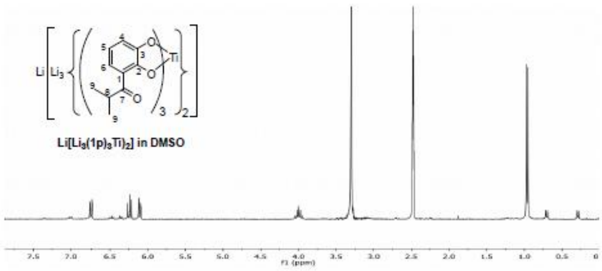

Li[$Li_3(1q)_3Ti)_2$]: **^{1}H-NMR** (400 MHz, Acetone-d_6): Dimer (major component): δ = 7.12 (dd, J = 8.0, 1.5 Hz, 1H, H_{arom}), 6.51 (t, J = 8.0 Hz, 1H, H_{arom}), 6.38 (dd, J = 8.0, 1.5 Hz, 1H, H_{arom}), 3.47-3.43 (m, 1H, COCH), 1.46-1.39 (m, 4H, 2×CH_2), 0.78 (t, J = 6.7 Hz, 6H, 2×CH_3) ppm. Monomer (minor component): δ = 3.75-3.73 (m, 1H, COCH), 1.69-1.60 (q, J = 6.7 Hz, 4H, 2×CH_2), 0.86 (t, J = 6.7 Hz, 6H, 2×CH_3) ppm. Other signals of the monomer are very low in intensity. **^{1}H-NMR** (600 MHz, ACN-d_3): Dimer (major component): δ = 7.10 (dd, J = 8.0, 1.5 Hz, 1H, H_{arom}), 6.57 (t, J = 8.0 Hz, 1H, H_{arom}), 6.50 (dd, J = 8.0, 1.5 Hz, 1H, H_{arom}), 2.80-2.78 (m, 1H, COCH), 1.36-1.29 (m, 2H, CH_2), 0.90-0.83 (m, 2H, CH_2), 0.63 (t, J = 6.7 Hz, 6H, 2×CH_3) ppm. Monomer (minor component): δ = 7.49 (dd, J = 8.0, 1.5 Hz, 1H, H_{arom}), 6.96 (t, J = 8.0 Hz, 1H, H_{arom}), 6.84 (dd, J = 8.0, 1.5 Hz, 1H, H_{arom}), 3.44-3.40 (m, 1H, COCH), 1.78-1.69 (m, 2H, CH_2), 1.66-1.63 (m, 2H, CH_2), 0.79 (t, J = 6.7 Hz, 6H, 2×CH_3) ppm. **^{1}H-NMR** (400 MHz, MeOH-d_4): Dimer (minor component): δ = 3.49-3.42 (m, 1H, COCH), 0.62 (t, J = 6.7 Hz, 6H, 2×CH_3) ppm. Other signals of the dimer are very low in intensity. Monomer (major component): δ = 6.99 (dd, J = 7.6, 2.1 Hz, 1H, H_{arom}), 6.51 – 6.40 (m, 2H, H_{arom}), 3.88 – 3.79 (m, 1H, COCH), 1.67-1.59 (m, 2H, CH_2), 1.40-1.32 (q, J = 6.7 Hz, 2H, CH_2), 0.77 (t, J = 6.7 Hz, 6H, 2×CH_3) ppm. **^{1}H-NMR** (600 MHz, DMSO-d_6): Only monomer: δ = 6.75 (dd, J = 8.1, 1.5 Hz, 1H, H_{arom}), 6.21 (t, J = 8.1 Hz, 1H, H_{arom}), 6.08 (dd, J = 8.1, 1.5 Hz, 1H, H_{arom}), 3.96-3.84 (m, 1H, H-8), 1.63-1.50 (m, 2H, CH_2), 1.35-1.22 (m, 2H, CH_2), 0.69 (t, J = 7.4 Hz, 6H, 2×CH_3) ppm. **^{1}H-NMR** (600 MHz, THF-d_8): Dimer (major component): δ = 6.90 (dd, J = 8.0, 1.6 Hz, 1H, H_{arom}), 6.31 (t, J = 8.0 Hz, 1H, H_{arom}), 6.23 (dd, J = 8.0, 1.6 Hz, 1H, H_{arom}), 3.21-3.19 (m, 1H, COCH), 1.35-1.29 (m, 4H, 2×CH_2), 0.70 (t, J = 6.5 Hz, 6H, 2×CH_3) ppm. Monomer (minor component): δ = 6.96 (dd, J = 8.0, 1.6 Hz, 1H, H_{arom}), 6.36 (dd, J = 8.0, 1.6 Hz, 1H, H_{arom}), 0.72 (d, J = 6.5 Hz, 6H, 2×CH_3) ppm. Signals not listed are overlapping and cannot be assigned. **MS** (positive ESI-MS, MeOH): m/z (%) = 1367.5460 (100, [M_D+Li^+], $C_{72}H_{84}O_{18}Li_5Ti_2^+$, calcd. 1367.5416), 687.28003 (50, [M_M+Li^+], $C_{36}H_{42}O_9Li_3Ti^+$, calcd. 687.2788). **IR (KBr)**: $\tilde{\nu}$ (cm^{-1}) = 3308, 3065, 2961, 2929, 2871, 2679, 2505, 2289, 2221, 2174, 2079, 2006, 1891, 1650, 1590, 1554, 1433, 1378, 1254, 1213, 1100, 1052, 915, 863, 804, 730, 663. **Elemental analysis:** $C_{72}H_{84}O_{18}Li_4Ti_2 \cdot 3\,H_2O$: calcd. C = 61.12 %, H = 6.41 %; found C = 61.19 %, H = 6.35 %.

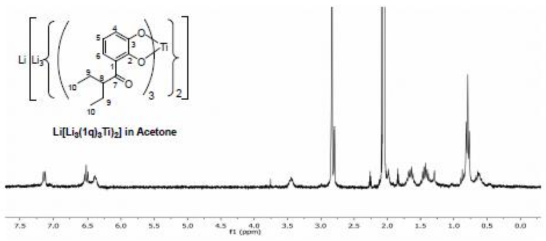

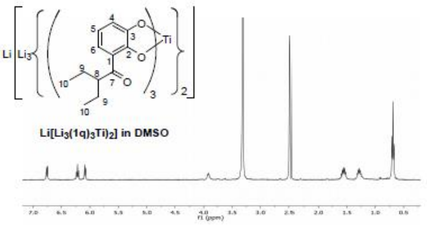

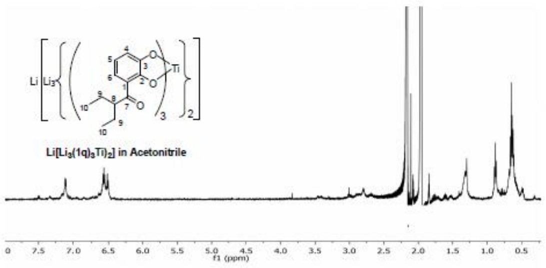

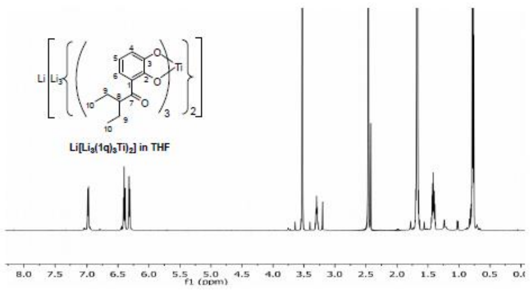

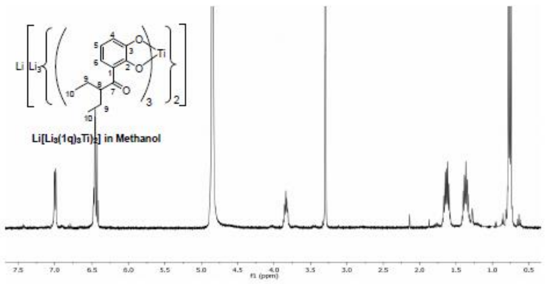

Li[Li$_3$(1r)$_3$Ti)$_2$]: **^{1}H-NMR** (400 MHz, Acetone-d$_6$): Dimer (major component): ***δ*** = 7.00 (dd, *J* = 7.9, 1.6 Hz, 1H, H_{arom}), 6.44 (t, *J* = 7.9 Hz, 1H, H_{arom}), 6.39 (dd, *J* = 7.9, 1.6 Hz, 1H, H_{arom}), 3.26-3.19 (m, 1H, $COCH_{cycl}$), 1.32-1.18 (m, 6H, H_{cycl}), 1.08-1.01 (m, 2H, H_{cycl}) ppm. Monomer (minor component): ***δ*** = 3.49-3.46 (m, 1H, $COCH_{cycl}$), 1.81-1.76 (m, 6H, H_{cycl}), 1.67-1.60 (m, 2H, H_{cycl}) ppm. Signals not listed are of very low intensity and cannot be assigned. **^{1}H-NMR** (600 MHz, ACN-d$_3$): Dimer (major component): ***δ*** = 7.07 (dd, *J* = 7.9, 1.6 Hz, 1H, H_{arom}), 6.54 (t, *J* = 7.9 Hz, 1H, H_{arom}), 6.47 (dd, *J* = 7.9, 1.6 Hz, 1H, H_{arom}), 3.27-3.22 (m, 1H, $COCH_{cycl}$), 1.85-1.80 (m, 2H, H_{cycl}), 1.08-1.01 (m, 2H, H_{cycl}) ppm. Monomer (minor component): ***δ*** = 3.49-3.46 (m, 1H, $COCH_{cycl}$), 1.81-1.76 (m, 6H, H_{cycl}), 1.67-1.60 (m, 2H, H_{cycl}) ppm. Signals not listed are of very low intensity and cannot be assigned. **^{1}H-NMR** (400 MHz, MeOH-d$_4$): Dimer (minor component): ***δ*** = 7.09 (dd, *J* = 8.2, 1.6 Hz, 1H, H_{arom}), 6.57-6.55 (m, 2H, H_{arom}), 3.23-3.21 (m, 1H, $COCH_{cycl}$) ppm. Monomer (major component): ***δ*** = 6.98 (dd, *J* = 8.2, 1.6 Hz, 1H, H_{arom}), 6.49-6.38 (m, 2H, H_{arom}), 4.21-4.13 (m, 1H, $COCH_{cycl}$) ppm. Signals not listed are overlapping and cannot be assigned. **^{1}H-NMR** (600 MHz, DMSO-d$_6$): Dimer (minor component): ***δ*** = 6.97 (dd, *J* = 8.2, 1.6 Hz, 1H, H_{arom}), 6.47 (t, *J* = 8.2 Hz, 1H, H_{arom}), 6.34 (dd, *J* = 8.2, 1.6 Hz, 1H, H_{arom}), 3.17-3.09 (m, 1H, $COCH_{cycl}$) ppm. Monomer (major component): ***δ*** = 6.75 (dd, *J* = 8.2, 1.6 Hz, 1H, H_{arom}), 6.23 (t, *J* = 8.2 Hz, 1H, H_{arom}), 6.09 (dd, *J* = 8.2, 1.6 Hz, 1H, H_{arom}), 4.28-4.23 (m, 1H, $COCH_{cycl}$) ppm. Signals not listed are overlapping and cannot be assigned. **^{1}H-NMR** (600 MHz, THF-d$_8$): Dimer (major component): ***δ*** = 7.04 (dd, *J* = 7.9, 1.6 Hz, 1H, H_{arom}), 6.44 (t, *J* = 7.9 Hz, 1H, H_{arom}), 6.36 (dd, *J* = 7.9, 1.6 Hz, 1H, H_{arom}), 3.78-3.73 (m, 1H, $COCH_{cycl}$) ppm. Monomer (minor component): ***δ*** = 7.36 (dd, *J* = 7.9, 1.6 Hz, 1H, H_{arom}), 6.31 (dd, *J* = 7.9, 1.6 Hz, 1H, H_{arom}), 3.82-3.79 (m, 1H, $COCH_{cycl}$) ppm. Signals not listed are overlapping and cannot be assigned. **MS** (negative and positive ESI-MS, MeOH): *m/z* (%) = 1341.4123 (100, [M_D-Li^+], $C_{72}H_{72}O_{18}Li_3Ti_2^-$, calcd. 1341.4158); 1355.4497 (100, [M_D+Li^+], $C_{72}H_{72}O_{18}Li_5Ti_2^+$, calcd. 1355.4478), 681.2318 (20, [M_M+Li^+], $C_{36}H_{36}O_9Li_3Ti^+$, calcd. 681.2319). **IR (KBr)**: $\tilde{\nu}$ (cm^{-1}) = 3854, 3392, 3064, 2948, 2867, 2654, 2322, 2159, 2113, 2010, 1975, 1935, 1708, 1648, 1591, 1548, 1427, 1355, 1253, 1212, 1118, 1054, 939, 864, 790, 733, 668.

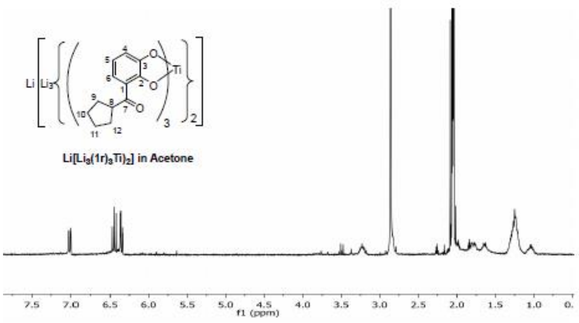

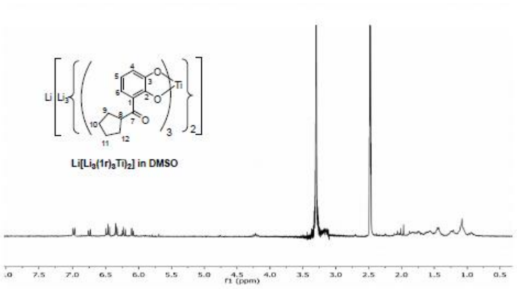

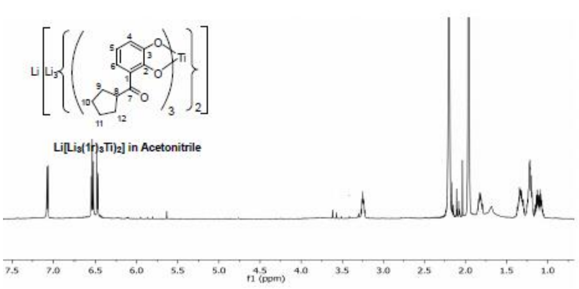

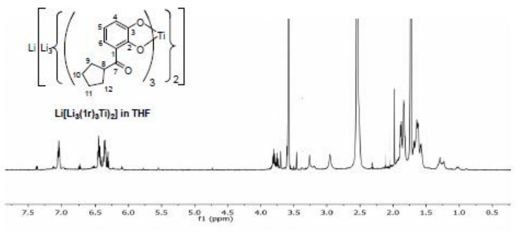

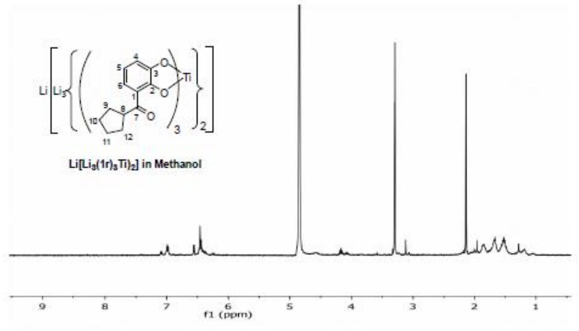

$Li[Li_3(1s)_3Ti)_2]$: ^{1}H-NMR (400 MHz, Acetone-d_6): Dimer (major component): δ = 6.97 (dd, J = 7.8, 1.5 Hz, 1H, H_{arom}), 6.44 (t, J = 7.8 Hz, 1H, H_{arom}), 6.34 (dd, J = 7.8, 1.5 Hz, 1H, H_{arom}), 2.80-2.75 (m, 1H, $COCH_{cycl}$) ppm. Monomer (minor component): δ = 7.47 (dd, J = 7.8, 1.5 Hz, 1H, H_{arom}), 7.11 (dd, J = 7.8, 1.5 Hz, 1H, H_{arom}), 6.83 (t, J = 7.8 Hz, 1H, H_{arom}), 3.45-3.38 (m, 1H, $COCH_{cycl}$) ppm. Signals not listed are overlapping and cannot be assigned. **^{1}H-NMR** (600 MHz, ACN-d_3): Dimer (major component): δ = 7.05 (dd, J = 7.9, 1.6 Hz, 1H, H_{arom}), 6.54 (t, J = 7.9 Hz, 1H, H_{arom}), 6.46 (dd, J = 7.9, 1.6 Hz, 1H, H_{arom}), 2.86-2.80 (m,1H, $COCH_{cycl}$) ppm. Monomer (minor component): δ = 7.48 (dd, J = 7.9, 1.6 Hz, 1H, H_{arom}), 6.84 (t, J = 7.9 Hz, 1H, H_{arom}), 6.71 (dd, J = 7.9, 1.6 Hz, 1H, H_{arom}), 3.44-3.41 (m, 1H, $COCH_{cycl}$) ppm. Signals not listed are overlapping and cannot be assigned. **^{1}H-NMR** (400 MHz, DMSO-d_6): Dimer (minor component): δ = 7.37 (dd, J = 7.9, 1.6 Hz, 1H, H_{arom}), 7.01 (dd, J = 7.9, 1.6 Hz, 1H, H_{arom}), 6.77 (t, J = 7.9 Hz, 1H, H_{arom}), 3.49-3.45 (m, 1H, $COCH_{cycl}$) ppm. Monomer (major component): δ = 6.95 (dd, J = 7.9, 1.6 Hz, 1H, H_{arom}), 6.47-6.39 (m, 2H, H_{arom}), 3.82-3.79 (m, 1H, $COCH_{cycl}$) ppm. Signals not listed are overlapping and cannot be assigned. **^{1}H-NMR** (600 MHz, DMSO-d_6): Only monomer: δ = 6.74 (dd, J = 8.2, 1.6 Hz, 1H, H_{arom}), 6.21 (t, J = 8.2 Hz, 1H, H_{arom}), 6.09 (dd, J = 8.2, 1.5 Hz, 1H, H_{arom}), 3.78-3.73 (m, 1H, $COCH_{cycl}$), 1.76-1.73 (m, 2H, H_{cycl}), 1.47-1.42 (m, 3H, H_{cycl}), 1.28-1.23 (m, 2H, H_{cycl}), 1.07-0.99 (m, 3H, H_{cycl}) ppm. **^{1}H-NMR** (600 MHz, THF-d_8): Dimer (major component): δ = 7.35 (dd, J = 7.8, 1.6 Hz, 1H, H_{arom}), 6.99 (dd, J = 7.8, 1.6 Hz, 1H, H_{arom}), 6.73 (t, J = 7.8 Hz, 1H, H_{arom}), 2.80-2.75 (m, 1H, H-8) ppm. Monomer (minor component): δ = 7.01 (dd, J = 7.8, 1.6 Hz, 1H, H_{arom}), 6.44 (t, J = 7.8 Hz, 1H, H_{arom}), 6.35 (dd, J = 7.8, 1.6 Hz, 1H, H_{arom}), 3.36-3.31 (m, 1H, $COCH_{cycl}$) ppm. Signals not listed are overlapping and cannot be assigned. **MS** (negative and positive ESI-MS, MeOH): *m/z* (%) = 1425.5087 (100, $[M_D\text{-}Li^+]$, $C_{78}H_{84}O_{18}Li_3Ti_2^-$, calcd. 1425.5096), 703.2375 (98, $[M_M\text{-}Li_2\text{+}H^+]$, $C_{39}H_{43}O_9Ti^-$, calcd. 703.2387); 1439.5420 (70, $[M_D\text{+}Li^+]$, $C_{78}H_{84}O_{18}Li_5Ti_2^+$, calcd. 1439.5416). **IR (KBr)**: $\tilde{\nu}$ (cm^{-1}) = 3856, 3367, 3064, 2924, 2851, 2662, 2534, 2445, 2324, 2248, 2169, 2109, 2022, 1941, 1649, 1591, 1547, 1425, 1342, 1252, 1212, 1169, 1111, 1055, 1006, 915, 889, 857, 767, 733, 672. **Elemental analysis:** $C_{78}H_{84}O_{18}Li_4Ti_2 \cdot 10\ H_2O$: calcd. C = 58.08 %, H = 6.50 %; found C = 58.29 %, H = 6.57 %.

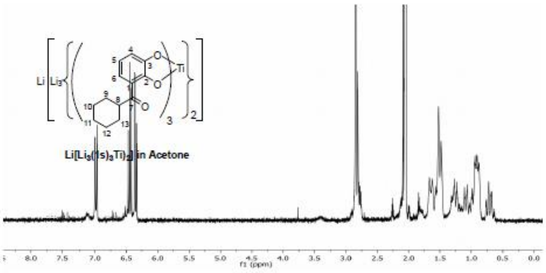

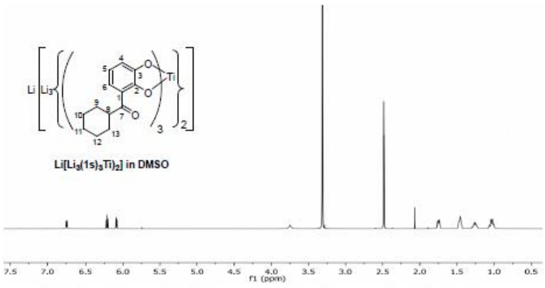

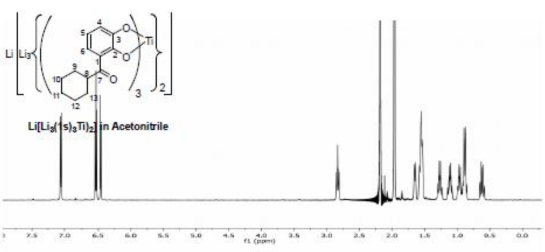

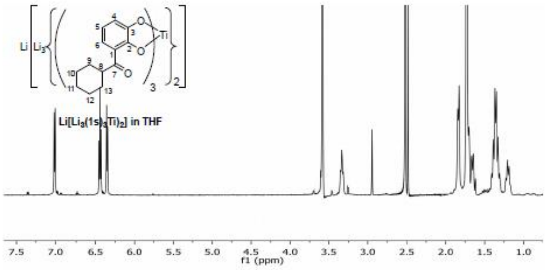

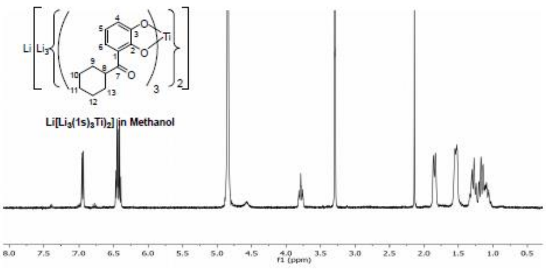

Li[$Li_3(1t)_3Ti)_2$]: **^{1}H-NMR** (400 MHz, Acetone-d_6): Dimer (major component): δ = 7.73 (dd, J = 8.0, 1.5 Hz, 1H, H_{arom}), 7.57 (t, J = 8.0 Hz, 1H, H_{arom}), 7.14 (t, J = 8.0 Hz, 2H, H_{arom}) ppm. Monomer (minor component): δ = 8.13 (s, 1H, H_{arom}), 7.67 (t, J = 8.0 Hz, 1H, H_{arom}), 7.09 (dd, J = 8.0, 1.5 Hz, 1H, H_{arom}) ppm. Signals not listed are overlapping and cannot be assigned. **^{1}H-NMR** (600 MHz, ACN-d_3): Dimer (minor component): δ = 7.57 (dd, J = 8.0, 1.5 Hz, 1H, H_{arom}), 7.21 (t, J = 8.0 Hz, 2H, H_{arom}), 6.89 (d, J = 8.0 Hz, 2H, H_{arom}), 6.70 (dd, J = 8.2, 1.5 Hz, 1H, H_{arom}), 6.57 (dd, J = 8.2, 1.5 Hz, 1H, H_{arom}), 6.43 (t, J = 8.0 Hz, 2H, H_{arom}) ppm. Monomer (major component): δ = 7.72 (d, J = 8.0 Hz, 2H, H_{arom}), 7.67 (t, J = 8.0 Hz, 1H, H_{arom}), 7.37 (t, J = 8.0 Hz, 2H, H_{arom}), 7.15 (dd, J = 8.2, 1.5 Hz, 1H, H_{arom}), 7.13 (dd, J = 8.2, 1.5 Hz, 1H, H_{arom}), 6.84 (t, J = 8.0 Hz, 1H, H_{arom}) ppm. **^{1}H-NMR** (400 MHz, MeOH-d_4): Dimer (minor component): δ = 7.61 (t, J = 8.0 Hz, 1H, H_{arom}), 7.43-7.35 (m, 1H, H_{arom}), 6.70-6.66 (m, 2H, H_{arom}) ppm. Monomer (major component): δ = 7.75-7.65 (m, 2H, H_{arom}), 7.51 (t, J = 8.0 Hz, 1H, H_{arom}), 7.24-7.19 (m, 2H, H_{arom}) ppm. Signals not listed are overlapping and cannot be assigned. **^{1}H-NMR** (600 MHz, DMSO-d_6): Dimer (minor component): δ = 7.53-7.46 (m, 1H, H_{arom}), 7.31-7.21 (m, 1H, H_{arom}) ppm. Monomer (major component): δ = 7.03-6.96 (m, 1H, H_{arom}), 6.42 (dd, J = 8.0, 1.5 Hz, 1H, H_{arom}), 6.33 (t, J = 8.0 Hz, 1H, H_{arom}) ppm. Signals not listed are overlapping and cannot be assigned. **^{1}H-NMR** (600 MHz, THF-d_8): Dimer (major component): δ = 7.56 (d, J = 7.5 Hz, 2H, H_{arom}),7.36 (t, t, J = 7.7 Hz, 2H, H_{arom}), 6.59 (t, J = 8.0 Hz, 1H, H_{arom}) ppm. Monomer (minor component): δ = 7.44 (t, J = 7.7 Hz, 2H, H_{arom}), 6.90 (t, J = 8.0 Hz, 2H, H_{arom}) ppm. Signals not listed are overlapping and cannot be assigned. **MS** (negative and positive ESI-MS, MeOH): m/z (%) = 1389.2211 (100, [M_D-Li^+], $C_{78}H_{48}O_{18}Li_3Ti_2^-$, calcd. 1389.2280); 1397.2528 (100, [M_D+H^+], $C_{78}H_{49}O_{18}Li_4Ti_2^+$, calcd. 1397.2510); 1403.2645 (70, [M_D+Li^+], $C_{78}H_{48}O_{18}Li_5Ti_2^+$, calcd. 1403.2600), 699.1304 (40, [M_M+H^+], $C_{39}H_{25}O_9Li_2Ti^+$, calcd. 699.1290). **IR (KBr)**: $\tilde{\nu}$ (cm^{-1}) = 3880, 3371, 3060, 2924, 2855, 2499, 2325, 2201, 2161, 2112, 2027, 1961, 1908, 1745, 1626, 1593, 1544, 1430, 1296, 1253, 1214, 1075, 1020, 932, 872, 825, 779, 704, 656. **Elemental analysis:** $C_{78}H_{48}O_{18}Li_4Ti_2 \cdot 9\ H_2O$: calcd. C = 60.10 %, H = 4.27 %; found C = 60.19 %, H = 4.35 %.

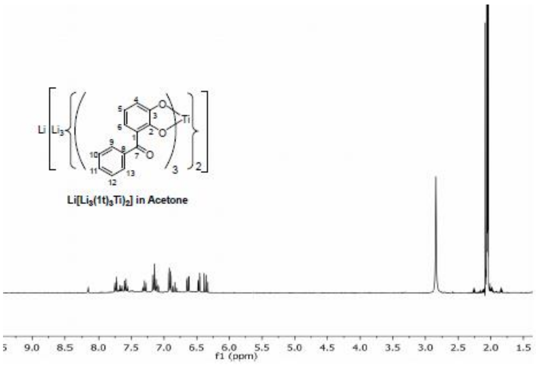

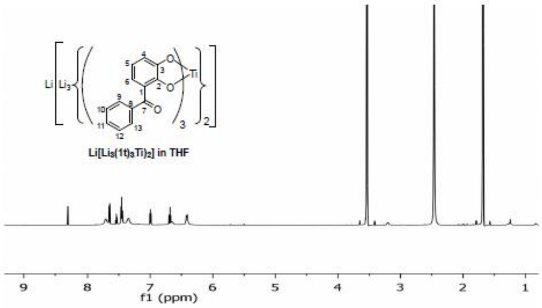

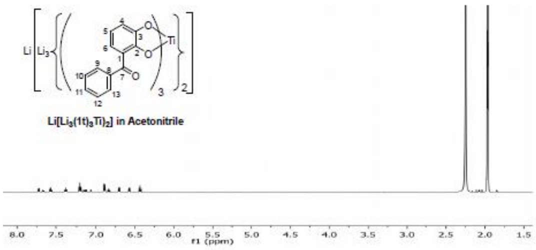

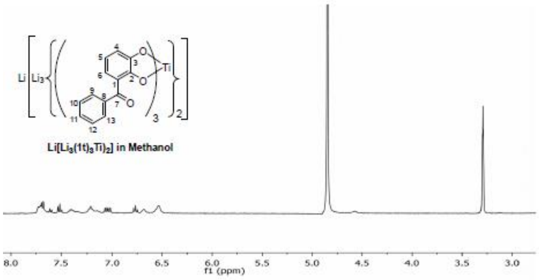

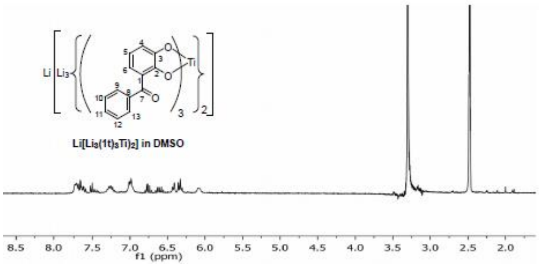

Li[$Li_3(1u)_3Ti)_2$]: **^{1}H-NMR** (300 MHz, Acetone-d_6): Dimer (major component): *δ* = 7.11-7.08 (m, 5H, H_{arom}), 6.50 (t, *J* = 7.5 Hz, 1H, H_{arom}), 6.41 (dd, *J* = 7.5, 1.5 Hz, 1H, H_{arom}), 4.28 (d, *J* = 14.0 Hz, 1H, $COCH_2$) ppm. Monomer (minor component): *δ* = 7.31-7.29 (m, 2H, H_{arom}) ppm. Signals not listed are overlapping and cannot be assigned. **^{1}H-NMR** (600 MHz, ACN-d_3): Dimer (major component): *δ* = 7.24 (t, *J* = 7.5 Hz, 1H, H_{arom}), 7.18-7.14 (m, 5H, H_{arom}), 4.23 (d, *J* = 14.0 Hz, 1H, $COCH_2$), 2.89 (d, *J* = 14.0 Hz, 1H, $COCH_2$) ppm. Monomer (minor component): *δ* = 7.29 (t, *J* = 7.5 Hz, 1H, H_{arom}), 6.96 (dd, *J* = 7.5, 1.5 Hz, 5H, H_{arom}) ppm. Signals not listed are overlapping and cannot be assigned. **^{1}H-NMR** (400 MHz, MeOH-d_4): Dimer (minor component): *δ* = 7.20 (dd, *J* = 7.7, 1.5 Hz, 1H, H_{arom}), 6.52-6.43 (m, 5H, H_{arom}), 2.87 (d, *J* = 8.0 Hz, 1H, $COCH_2$), 2.15 (d, *J* = 8.0 Hz, 1H, $COCH_2$) ppm. Monomer (major component): *δ* = 7.25 (dd, *J* = 7.7, 1.5 Hz, 1H, H_{arom}), 6.90-6.82 (m, 5H, $H_{arom,}$), 4.49 (s, 2H, $COCH_2$) ppm. Signals not listed are overlapping and cannot be assigned. **^{1}H-NMR** (600 MHz, DMSO-d_6): Dimer (minor component): *δ* = 6.54 (t, *J* = 7.3 Hz, 1H, H_{arom}), 6.42 (dd, *J* = 7.3, 1.7 Hz, 1H, H_{arom}), 4.28 (d, *J* = 8.0 Hz, 1H, H-8), 3.11 (d, *J* = 8.0 Hz, 1H, H-8) ppm. Monomer (major component): *δ* = 6.30 (t, *J* = 7.3 Hz, 1H, H_{arom}), 6.19 (dd, *J* = 7.3, 1.7 Hz, 1H, $H_{arom,}$), 4.37 (s, 2H, $COCH_2$) ppm. Signals not listed are overlapping and cannot be assigned. **^{1}H-NMR** (600 MHz, THF-d_8): Dimer (major component): *δ* = 7.09-7.07 (m, 2H, H_{arom}), 2.75 (d, *J* = 8.0 Hz, 1H, H-8), 2.36 (d, *J* = 8.0 Hz, 1H, $COCH_2$) ppm. Monomer (minor component): *δ* = 6.32 (t, *J* = 7.3 Hz, 1H, H_{arom}), 6.21 (dd, *J* = 7.3, 1.7 Hz, 1H, $H_{arom,}$), 4.11 (s, 2H, $COCH_2$) ppm. Signals not listed are overlapping and cannot be assigned.

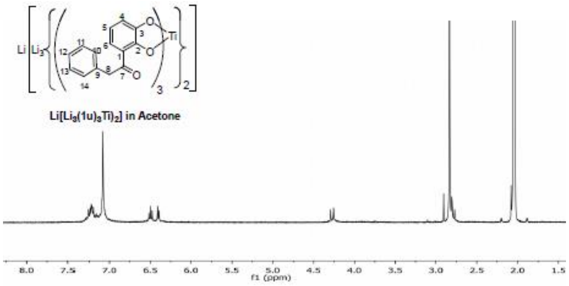

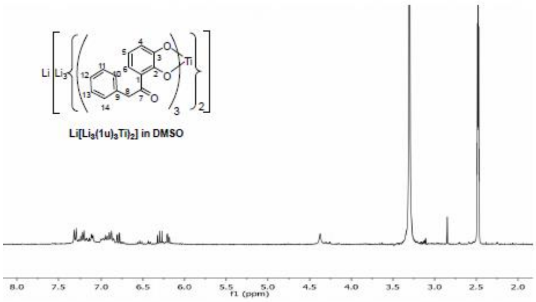

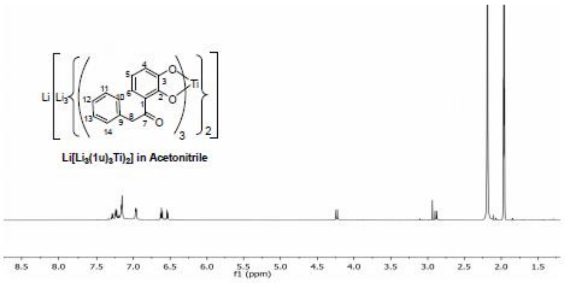

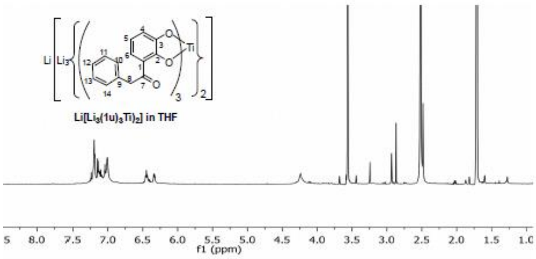

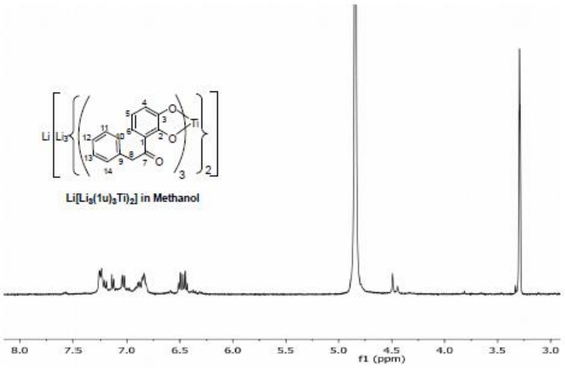

Crystal data and refinement parameters

($(K[Li_3(1o)_6Ti_2])_n$)

Single crystal X-ray diffraction data set was collected at 100(2) K by using ω-scans on a Stoe Stadivari diffractometer with a Eulerian 4 circle geometry, equipped with a CuK_α micro focus source (GeniX 3D HF Cu, λ = 1.54178 Å) and a Pilatus 200 K hybrid pixel detector (Dectris). Data collection and absorption correction of the diffraction intensities were performed with the software package X-Area (2018)[3]. The space group was determined with XPREP (1997)[4]. Primary structure solution was performed by using a dual-space method with SHELXT (2018/2)[5]; structure refinement was done using SHELXL (2018/3)[6] with a least squares procedure against F^2. For non-H atoms anisotropic displacement parameters were assigned. The positional disordered methyl cyclohexyl group and the coordinated solvent moieties (water, methanol, diethyl ether) were left isotropic. No hydrogen atoms were assigned for these solvent molecules but were considered in the sum formula calculation of the density. All other hydrogen atoms were refined using riding models with U_{eq}(H) of 1.2 of U_{eq}(C). Electron densities of co-crystallized solvent were removed by using the SQUEEZE routine in the program PLATON[7]. The solvent accessible volume, for which it is not possible to make reliable assignments, amounts to 459 Å^3 with 69 electrons.

$Fw(C_{89}H_{112}KLi_3O_{21}Ti_2)$ = 1673.50 g mol^{-1}; monoclinic, $P2_1/c$; a = 14.053(3), b = 22.217(4), c = 28.632(6) Å; β = 103.32(3)°; V = 8699(3) Å^3; Z = 4; ρ = 1.278 Mg m^{-3}. Refinement: R_1 = 0.0624 [$I > 2\sigma(I)$], wR_2 = 0.1724 (all data), S = 0.950.

CCDC 1986720 contains the supplementary crystallographic data for this paper. The data can be obtained free of charge from The Cambridge Crystallographic Data Centre via www.ccdc.cam.ac.uk/structures.

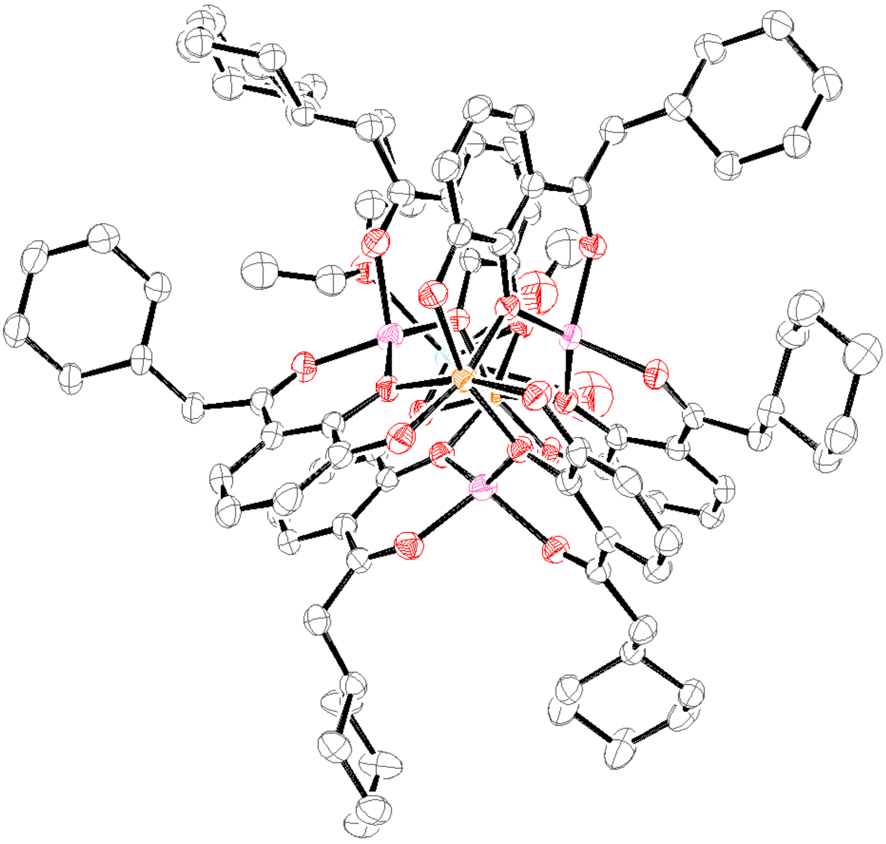

Figure 1: ORTEP-plot[8] of the asymmetric unit in $K[Li_3(\mathbf{1o})_6Ti_2]$ with 50% thermal ellipsoid probability level. C grey, Li pink, O red, Ti orange, K light blue. Hydrogens are omitted for clarity.

($(Li[Li_3(1s)_6Ti_2])_n$)

Single crystal X-ray diffraction data set was collected in the same way as described for the compound above. Also, the structure determination was performed as mentioned. For non-H atoms anisotropic displacement parameters were assigned. The positional disordered Lithium-cation was fixed by restraints and left isotropic. No hydrogen atom was

assigned for the hydroxy group of the coordinated methanol solvent molecule but is considered in the sum formula. All other hydrogen atoms were refined using riding models with U_{eq}(H) of 1.5U_{eq}(C) for terminal methyl groups, and 1.2 of U_{eq}(C) for other groups. Electron densities of co-crystallized solvent were removed by using the SQUEEZE routine in the program PLATON[7]. The solvent accessible volume, for which it is not possible to make reliable assignments, amounts to 650 Å^3 with 185 electrons.

F_w($C_{80}H_{92}Li_4O_{20}Ti_2$) = 1497.09 g mol^{-1}; orthorhombic, *Pbcn;* a = 11.971(2), b = 26.828(5), c = 24.897(5) Å; V = 7996(3) Å^3; Z = 4; ρ = 1.244 Mg m^{-3}. Refinement: R_1 = 0.0827 [$I > 2\sigma(I)$], wR_2 = 0.2403 (all data), S = 1.072.

CCDC 1986851 contains the supplementary crystallographic data for this paper. The data can be obtained free of charge from The Cambridge Crystallographic Data Centre via www.ccdc.cam.ac.uk/structures.

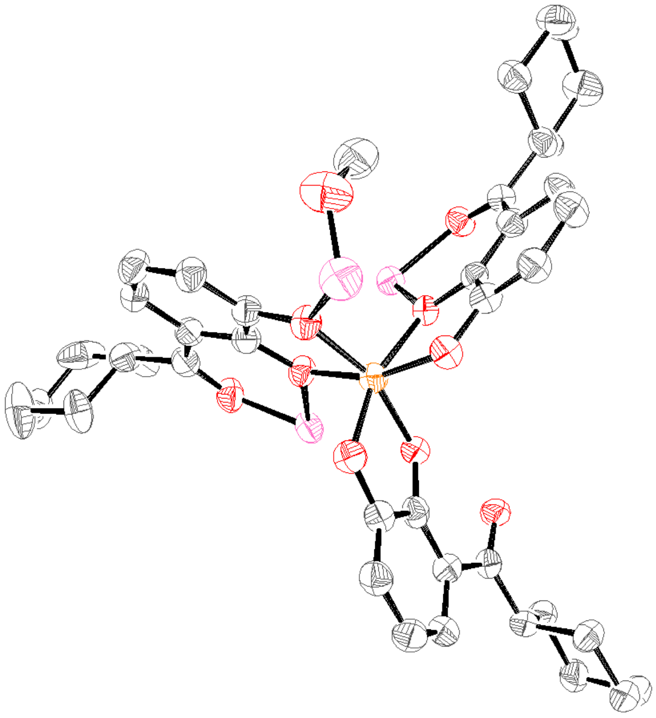

*Figure 2: ORTEP-plot[8] of the asymmetric unit in Li[Li$_3$(**1s**)$_6$Ti$_2$] with 50% thermal ellipsoid probability level. C grey, Li pink, O red, Ti orange. Hydrogens are omitted for clarity*

---

[1] (a) D. Van Craen, W. H. Rath, M. Huth, L. Kemp, C. Räuber, J. Wollschläger, C. Schalley, A. Valkonen, K. Rissanen, M. Albrecht, *J. Am. Chem. Soc.* **2017,** *139*, 16959–16966. (b) A. C. N. Kwamen, M. Schlottmann, D. Van Craen, E. Isaak, J. Baums, L. Shen, A. Massomi, C. Räuber, B. P. Joseph, G. Raabe, C. Göb, I. M. Oppel, R. Puttreddy, J. S. Ward, K. Rissanen, R. Fröhlich, M. Albrecht, *Chem. Eur. J.* **2020**, *26*, 1396-1405.
[2] M. Albrecht, S. Mirtschin, M. de Groot, I. Janser, J. Runsink, G. Raabe, M. Kogej, C. A. Schalley, R. Fröhlich, *J. Am. Chem. Soc.*, **2005**, *127*, 10371-10387.
[3] *X-AREA V 1.82*, Stoe & Cie GmbH **2018**.
[4] *XPREP V 5.1*, Bruker Analytical X-ray Systems **1997**.
[5] G. M. Sheldrick, *Acta Cryst.* **2015**, *A71*, 3-8.
[6] G. M. Sheldrick, *Acta Cryst.* **2015**, *C71*, 3-8.
[7] L. A. Spek, *Acta Cryst.* **2015**, *C71*, 9-18.
[8] L. J. Farrugia, *J. Appl. Cryst.* **2012,** *45*, 849-854.
